# Farming Activities and Risk of Inflammatory Bowel Disease: A French Nationwide Population-based Cohort Study

**DOI:** 10.1093/ecco-jcc/jjae050

**Published:** 2024-04-12

**Authors:** Pascal Petit, Ariane Leroyer, Sylvain Chamot, Mathurin Fumery, Vincent Bonneterre

**Affiliations:** Univ. Grenoble Alpes, AGEIS, Grenoble, France; CHU Grenoble Alpes, Centre Régional de Pathologies Professionnelles et Environnementales, Grenoble, France; Univ. Lille, Inserm, CHU Lille, U1286 – INFINITE – Institute for Translational Research in Inflammation, Lille, France; Regional Center for Occupational and Environmental Diseases of Hauts-de-France, Amiens University Hospital, Amiens, France; Péritox [UMR_I 01]; UPJV/INERIS; University of Picardy Jules Verne, Amiens, France; Péritox [UMR_I 01]; UPJV/INERIS; University of Picardy Jules Verne, Amiens, France; Gastroenterology Department, CHU Amiens-Picardie, Amiens, France; CHU Grenoble Alpes, Centre Régional de Pathologies Professionnelles et Environnementales, Grenoble, France; Univ. Grenoble Alpes, CNRS, UMR 5525, VetAgro Sup, Grenoble INP, CHU Grenoble Alpes, Grenoble, France

**Keywords:** Environmental epidemiology and health surveillance, agricultural exposome, digital environmental and public health

## Abstract

**Background and Aims:**

Epidemiological data regarding inflammatory bowel disease [IBD] are lacking, in particular for occupationally exposed populations. We investigated whether, among the entire French farm manager [FM] workforce, certain agricultural activities are more strongly associated with IBD than others.

**Methods:**

Nationwide, population-based, insurance claims and electronic health records from all FMs who worked at least once over the period 2002–2016 were used [*n* = 1 088 561, 69% males]. The outcome measure was the association between 26 farming activities and the risk of IBD, Crohn’s disease [CD], and ulcerative colitis [UC], measured as hazard ratios [HRs], after adjusting for age, sex, pre-existing medical comorbidities, and farm location. The time to first chronic disease declaration was used as the underlying time scale. A model was generated for every activity and disease, using a reference group comprising all FMs who abstained from the specified activity from 2002 to 2016.

**Results:**

There were 1752 IBD cases, with 704 CD [40.2%] and 1048 UC [59.8%] cases, respectively. Elevated HRs were observed for fruit arboriculture [HR from 1.17 to 1.52] and dairy farming [HR from 1.22 to 1.46] for all IBD, in crop farming for CD only (HR = 1.26, 95% confidence interval [CI]: 1.06–1.49), and in shellfish farming [HR from 2.12 to 2.51] for both CD and IBD.

**Conclusions:**

Further research regarding specific farming activities and exposures likely to modify the microbiota [eg, pesticides, pathogens] is required to identify potential occupational risk factors [agricultural exposome] for IBD. Exposure to *Mycobacterium avium subspecies paratuberculosis*, Cryptosporidium, environmental toxins, micro/nanoplastics, and pesticides represents promising research avenues.

## Graphical Abstract

**Graphical Abstract:**
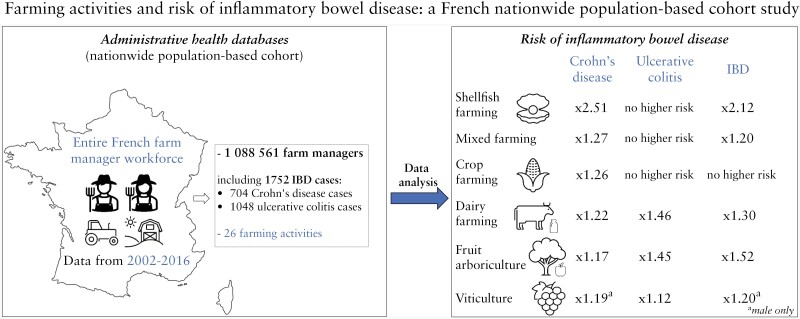

## 1. Introduction

Inflammatory bowel diseases [IBD] are multifactorial, idiopathic, chronic, immune-mediated diseases affecting the gastrointestinal tract.^1–4^ There are two main IBDs, namely Crohn’s disease [CD] and ulcerative colitis [UC], which have different phenotypic presentations.^1,5–8^ The aetiology and pathogenesis of IBD are complex and remain unclear, with potential interplay between the immune system, gut microbiome, genetics, and environmental factors.^1,2,4–7,9–12^ Over 2.5 million people in Europe and 1.5 million in North America are estimated to be suffering from IBD.^9,13^ IBD is a major worldwide public health issue with a continually increasing incidence, and that exerts a major toll on patients [disability-adjusted life-years].

The aetiology of IBD is believed to be multifactorial, including both genetic and environmental factors [exposome].^9,14–16^ The increasing incidence of IBD in industrialised countries and the increased risk observed in migrants moving into high-prevalence areas advocate that the exposome is an important determinant of IBD susceptibility and severity. Suggested environmental factors include smoking,^5,6,10,11,14,16–19^ diet,^5,6,9–11,14–16,18–20^ hygiene,^9,10,17^ medications,^5,6,9,11,16,17,19,20^ psychosocial stress,^16,18–22^ depression,^16,21^ air pollution,^12,14,15,20,23^ physical activity,^5,6,9,11,14,17^ and exposure to pathogens^10,11,14,19,20,24–29^ and chemicals,^27,30–34^ but data are still inconclusive. With the exception of smoking, no well-established protective/risk factors have been identified. There is a need for population-based epidemiological cohort studies, in particular for occupational exposure, for which data are lacking.^15,27^ Indeed, there is increasing evidence pertaining to the association between autoimmune diseases and occupational exposure [eg, silica, solvents, and pesticides].^27,33,35^

Farmers are an understudied population that could be at risk of IBD because they are exposed to some of the aforementioned environmental stressors [agricultural exposome],^36^ in particular psychosocial stress,^37–40^ pathogens,^10,11,26–28,41^ and chemicals such as pesticides, metals, or micro- and nanoplastics.^30–32,34,42^ Given the heterogeneity and broad range of agricultural practices, farming activities differ from one another in terms of tasks performed and associated exposome. For instance, livestock farmers are more likely to be exposed to different factors [eg, pathogens and stress] than pesticide applicators. Hence, IBD risks could potentially differ between farming activities, in particular since IBD may most likely arise from the concurrent interaction of multiple causal agents and triggering factors [agricultural exposome].

To our knowledge, only one study focusing on male pesticide applicators and their spouses has investigated potential risk factors for IBD in the farming population.^31^ However, as of now, no study has explored IBD risks related to specific agricultural activities. To tackle this issue, we explore whether distinct farming activities within the entire population of French farm managers [FMs] exhibit a higher association with IBD compared with other activities. This study encompasses an analysis of the overall population as well as sex-specific analyses.

## 2. Methods

### 2.1. Data source and study population

This study is a component of the TRACTOR project, which aims to track and monitor occupational risks in agriculture.^36,43–46^ We relied on routinely collected digital administrative health data from the national agricultural insurance scheme, Mutualité Sociale Agricole [MSA], which covers all French farmers [around 5% of the French population]. From October 2018 to December 2020, fully anonymised MSA data were obtained and managed for the TRACTOR project.^43^

Our study encompassed the entire French farm manager workforce, excluding farm employees and seasonal workers, and data from overseas French territories. The term ‘FM’ refers to a broad category that includes farm/company managers, owners, and self-employed individuals who engage in various farming activities in the field as part of their occupation.^36,45,46^

For this work, we used both insurance claims and electronic health/medical records. Every year, the MSA routinely gathers administrative data, specifically insurance claims, through self-reported forms submitted by FMs as part of their mandatory annual insurance enrolment process. These claims include sociodemographics as well as the main FM’s activity in terms of effective working time during a given year. The farming activities are self-reported by each FM, selecting from 26 available options [eg, pig farming] that correspond to a national categorisation of different farming practices outlined in French legislation.^36,45,46^ The complete aforementioned MSA insurance claim data spanning 2002–2016 [ie, sociodemographics and farming activity] were used for this study.

Regarding electronic health/medical records, only data from 2012 to 2016 were available. These complete digital health records, specifically disease declarations from 2012 to 2016, were used for follow-up purposes. The baseline time point, denoted as time zero, was set on January 1, 2012, and the follow-up concluded on December 31, 2016. The median follow-up was estimated using the Kaplan–Meier reverse method. The prevalence proportion was defined as the proportion of IBD cases at the end of follow-up, and incident IBD cases were defined as cases occurring after baseline.

The subsequent analysis of these data was carried out between April 2023 and March 2024 and approved by the French independent administrative authority protecting privacy and personal data, which waived the need for informed consent. All procedures were conducted in compliance with the appropriate protocols and regulations. This investigation adhered to the Strengthening the Reporting of Observational Studies in Epidemiology [STROBE] reporting guideline, as detailed in the Supplementary material.

### 2.2. Case identification

Long-term illness [LTI] declarations from electronic health/medical records were used to identify IBD cases. LTIs, a specificity of the French insurance scheme, are declarations made by the physician to the insurance scheme. LTIs award FM fee exemptions and full coverage of health care expenditures related to the chronic illness declared. Each LTI is coded with an ICD-10 code [10th revision of the International Statistical Classification of Diseases and Related Health Problems]. IBD is among the illnesses covered by the LTI scheme. ICD-10 codes K50 (‘Crohn’s disease’ [regional enteritis]) and K51 [‘ulcerative colitis’] were used to identify CD and UC cases, respectively. FMs who had either K50 or K51 codes were considered IBD cases. Following previously published and validated algorithms,^47–50^ to be considered a case, a FM needed to have at least one LTI declaration for IBD between 2012 and 2016. Using the LTI declaration for case identification is a specific and sensitive approach because, in France, most patients [87%] diagnosed with an IBD are awarded an LTI.^47,51^ This LTI declaration is valid for 2 years and must be renewed when it expires.^52^ IBD patients with no LTI have benign forms.

### 2.3. Statistical analysis

To examine the potential disparity in the association [estimated as hazard ratio, HR] between 26 farming activities and IBD risk, the Cox proportional hazards model was carried out. Whenever there was a minimum of three exposed cases, a distinct model was generated for each farming activity, with the reference group being comprised of all FMs who did not partake in the given activity, whereas the exposed group included all FMs who engaged in the specific activity [eg, fruit arboriculture] at least once during the study period. In each model, the dependent variables consisted of the time scale, which was measured on a continuous scale, and the disease diagnosis [LTI declaration for IBD], which was categorised as either yes or no. The time to initial LTI declaration for IBD [disease diagnosis] was used as the underlying time scale. For FMs with IBD, the end of follow-up was defined as the earliest disease declaration date. Regarding FMs who did not experience the event of interest [IBD], survival was censored at the date of leaving the study due to either loss to follow-up [eg, FM no longer being affiliated with MSA] or end of follow-up time on December 31, 2016.

Based on published literature and expert knowledge, a directed acyclic graph was built to identify a minimally sufficient adjustment set and to guide covariate selection, which included both confounders [eg, age, sex, farm location] and predictors [eg, farming activity] of IBD [Supplementary Figure S1]. All analyses were adjusted for age [continuous], sex [categorical: female and male], first year of the farm’s establishment [continuous], number of years performing the studied activity [continuous], number of pre-existing medical comorbidities [continuous], and farm location [categorical: 96 French administrative geographical departments].

In order to evaluate potential disparities between sexes, interaction tests were conducted by incorporating interaction terms into the model. Additionally, sex-specific analyses, involving the creation of separate models for each sex, were carried out to ascertain sex-specific risk estimates.

Sensitivity analyses [SAs] were conducted to further refine the analyses and reduce potential sources of bias. Three SAs were carried out to assess the influence of pre-existing medical comorbidities on the risk of IBD, as multimorbidity and various comorbidities have been linked to IBD, in particular stress and depression.^5,6,9,19,21,22^ These analyses were adjusted for the same variables as the main analysis, with the exception of pre-existing medical comorbidities. The main analysis accounted for the number of pre-existing medical comorbidities, and SA1 was adjusted for the 30 LTIs specified by French laws. In SA2, no adjustment on comorbidity was performed, and SA3 was adjusted only for antidepressant drug reimbursement and LTIs pertaining to mental health issues such as depression.

To examine exposure-response trends and ascertain exposure-specific risk estimates, two additional sensitivity analyses were performed [SA4 and SA5]. For SA4, the exposed group was restricted to all FMs who engaged in the specific activity for less than the median number of years of exposure of all exposed FMs [eg, <5 years]. Regarding SA5, the exposed group was restricted to all FMs who engaged in the specific activity for at least the median number of years of exposure of all exposed FMs [eg, ≥5 years]. Both SA4 and SA5 were adjusted for the same variables as the main analysis. The HR of IBD associated with a 1-year increase in exposure [ie, performing a given farming activity] from the main analysis model was also provided.

To increase the likelihood that IBD cases identified using LTI declarations are incident cases, a sensitivity analysis [SA6] excluding IBD cases identified in 2012 was conducted. This analysis was adjusted for the same variables as the main analysis.

For every model, the validity of the assumption of proportional hazard rate was evaluated by examining the independence between scaled Schoenfeld’s residuals and time. When this assumption was not satisfied for a particular covariate, an interaction term [covariate*time] was included in the model. To tackle the problem of multiple testing, the Benjamini–Hochberg approach was applied. All statistical analyses were conducted using R software 4.3.1 [R Core Team, Vienna] for Windows 10.

## 3. Results

### 3.1. Population characteristics

The characteristics of the study population are presented in Supplementary Table S1. From 2002 to 2016, 1 088 561 FMs [69.0% males] performed one farming activity at least once. FMs with an IBD were younger, had a bigger farm surface, lived more often in the North of France, worked more often with work partners/associates, had more often no secondary activity [ie, did not engage in another farming activity in parallel/addition to their main activity], paid higher insurance premiums, had more employees, and had more pre-existing comorbidities than FMs without an IBD. No FM whose first exposure year occurred during the follow-up period [2012–2016] was diagnosed with IBD before engaging in a given farming activity.

Regarding CD, there were 704 cases [30% females] over the period 2012–2016, with a median follow-up of 1047 [95% CI: 28–1826] days. The prevalence proportion was 4.21 [95% CI: 4.06–4.38] per 1000 persons, with 4.96 [4.66–5.32] per 1000 persons for females and 3.94 [3.77–4.13] per 1000 persons for males. The incidence rate was 0.225 [95% CI: 0.208–0.241] cases per 1000 person-years, with 0.254 [0.220–0.288] cases per 1000 person-years for females and 0.214 [0.195–0.233] cases per 1000 person-years for males, respectively.

Regarding UC, there were 1048 cases [25% females] over the period 2012–2016, with a median follow-up of 1064 [64–1825] days. The prevalence proportion was 6.27 [6.10–6.48] per 1000 persons, with 6.13 [5.80–6.53] per 1000 persons for females and 6.33 [3.12–6.57] per 1000 persons for males. The incidence rate was 0.335 [0.314–0.355] cases per 1000 person-years, with 0.316 [0.278–0.354] cases per 1000 person-years for females and 0.341 [0.318–0.365] cases per 1000 person-years for males, respectively.

Regarding IBD, there were 1752 cases [27% females] over the period 2012–2016, with a median follow-up of 1046 [28–1826] days. The prevalence proportion was 12.2 [11.9–12.4] per 1000 persons, with 12.7 [12.2–13.3] per 1000 persons for females and 12.0 [11.7–12.3] per 1000 persons for males. The incidence rate was 0.559 [0.533–0.586] cases per 1000 person-years, with 0.570 [0.519–0.621] cases per 1000 person-years for females and 0.556 [0.525–0.586] cases per 1000 person-years for males, respectively.

### 3.2. CD risk associated with agricultural activities

Associations varied by sex and types of agricultural activities, with several positive associations observed [Supplementary Table S2]. An elevated HR was found for shellfish farming (HR = 2.51 [95% CI: 1.19–5.30]), and modestly elevated HRs were observed for unspecified and mixed farming (HR = 1.27 [1.02–1.58]), crop farming (HR = 1.26 [1.06–1.49]), dairy farming (HR = 1.22 [1.10–1.39]), and fruit arboriculture (HR = 1.17 [1.04–1.76]), compared with those that never engaged in these activities. Four activities exhibited sex differences [Table 1], with HRs always more elevated in females than in males, with the exception of viticulture (HR = 1.10 [0.73–1.65] vs HR = 1.19 [1.04–1.58]). HRs were 3.01, 2.39, and 1.49 times more elevated in females than in males for shellfish farming, ovine and caprine farming, and fruit arboriculture, respectively.

**Table 1 .** Results of the interaction tests regarding the sex.

Agricultural activity	Crohn’s disease		Ulcerative colitis		IBD	
	*p*	Difference direction	*p*	Difference direction	*p*	Difference direction
Truck farming, floriculture/flower-growing	0.75	M > F	0.22	M > F	0.28	M > F
Fruit arboriculture	**0.05**	**F > M**	0.60	F > M	0.30	F > M
Garden centre/tree nursery	0.94	M > F	0.09	M > F	0.27	M > F
Crop farming [eg, wheat, corn, and industrial grower]	0.13	M > F	0.30	M > F	0.09	M > F
Viticulture	**0.03**	M > F	**0.05**	M > F	**0.04**	M > F
Sylviculture/forestry [eg, thinning, pruning]	not calculated		not calculated		not calculated	
Unspecified specialised farming [eg, herbs, mushrooms]	0.45	M > F	0.41	F > M	0.99	F > M
Dairy farming	0.29	M > F	**0.04**	F > M	0.26	F > M
Cow farming	0.35	F > M	0.89	M > F	0.67	F > M
Mixed cattle farming	0.52	M > F	0.39	M > F	0.25	M > F
Ovine and caprine farming	**0.01**	F > M	0.32	F > M	**0.01**	F > M
Pig farming	0.36	F > M	0.18	F > M	0.10	F > M
Stud farming	0.91	M > F	0.87	M > F	0.86	M > F
Unspecified large animal farming [eg, ostrich, llama]	not calculated		not calculated		not calculated	
Poultry and rabbit farming	0.75	F > M	0.24	F > M	0.28	F > M
Unspecified small animal farming [eg, frogs, snails, bees]	0.87	M > F	0.64	M > F	0.66	M > F
Training, dressage, riding clubs	0.10	F > M	0.18	F > M	**0.03**	F > M
Shellfish farming [eg, oyster farming, scallop aquaculture]	**0.05**	**F > M**	0.98	F > M	0.20	F > M
Unspecified and mixed farming [eg, polyculture, mixed farming, diversified farming]	0.50	M > F	0.11	M > F	0.11	M > F
Salt works/salt evaporation pond	not calculated		not calculated		not calculated	
Wood production [eg, lopping]	0.68	M > F	0.56	M > F	0.47	M > F
Stationary sawmill [eg, edging, trimming, decking, debarking]	not calculated		not calculated		not calculated	
Agricultural work companies [eg, pesticide applications, harvest reaping]	0.99	M > F	0.89	F > M	0.95	F > M
Gardening, landscaping, and reforestation companies	0.10	M > F	0.13	F > M	0.80	F > M
Company representative/authorised representative	not calculated		0.85	M > F	0.76	M > F
Rural craftsperson [eg, mason, mechanics]	not calculated		not calculated		not calculated	

F > M, risk higher for females than males; M > F, risk higher for males than females; IBD, inflammatory bowel diseases. *p*-values ≤0.05 are represented in bold font.

All SAs carried out to assess the influence of pre-existing medical comorbidities on the risk of CD yielded similar results to those of the main analysis [Supplementary Figures S2–S4, Table S4]. There were two exceptions regarding SA1 [adjusted for 30 LTIs], with mixed cattle farming having a modestly elevated HR (HR = 1.26 [1.02–1.57]), whereas unspecified small animal farming had a reduced HR (HR = 0.65 [0.46–0.97]), contrary to the other analyses. There was also an exception for female crop farmers who had an elevated HR (HR = 1.66 [1.19–2.30]) with SA2 [no adjustment for comorbidities] but not with the other analyses.

Regarding exposure-response trends [SA4 and SA5], no positive [ie, elevated HR] or negative [ie, reduced HR] association was found when considering the least exposed group [<median number of years of exposure] [SA4]. By contrast, when considering the highest exposed group [≥median number of years of exposure] [SA5], an elevated HR was observed for shellfish farming (HR = 2.37 [1.06–5.29]). Modestly elevated HRs were also found with SA5 in fruit arboriculture (HR = 1.24 [1.16–1.97]), dairy farming (HR = 1.23 [1.13–1.43]), crop farming (HR = 1.23 [1.02–1.48]), and viticulture (HR = 1.13 [1.01–1.48]).

A 1-year increase in exposure resulted in modestly elevated HRs for several activities [Supplementary Table S7]. An increase of 1 year in engaging in crop farming translated to an increase of 0.7% in the risk of CD (HR = 1.007 [1.002–1.072]). A similar increase was observed for shellfish farming (HR = 1.007 [1.001–1.081]). A 1-year increase in performing dairy farming and fruit arboriculture resulted in an increase of 0.9% (HR = 1.009 [1.003–1.045]) and 0.6% (HR = 1.006 [1.001–1.053]), respectively. Regarding viticulture, a 1-year increase in exposure translated to an increase of 0.8% in the risk of CD, but only in males (HR = 1.008 [1.001–1.057]).

The sensitivity analysis restricted to CD cases identified from 2013 to 2016 [SA6] yielded similar results to the main analysis that considered cases identified from 2012 to 2016. There was only one exception, with ovine and caprine farming, that had an elevated HR in females only (HR = 2.18 [1.27–3.74]), contrary to the main analysis.

### 3.3. UC risk associated with agricultural activities

Compared with other activities, elevated HRs of UC were observed for dairy farming (HR = 1.46 [1.25–1.70]) and fruit arboriculture (HR = 1.45 [1.01–2.70]), and viticulture had a modestly elevated HR (HR = 1.12 [1.01–1.28]) [Supplementary Table S3]. No activity exhibited a negative association [ie, a reduced HR]. Two activities exhibited sex differences, with more elevated HRs in females than in males for dairy farming (HR = 1.74 [1.32–2.29] vs HR = 1.32 [1.09–1.58]) and a positive association in males engaged in viticulture but not in females (HR = 0.93 [0.63–1.35] vs HR = 1.20 [1.04–1.49]).

All SAs performed to assess the influence of pre-existing medical comorbidities on the risk of UC yielded results similar to those of the main analysis [Supplementary Figures S5–S7, Table S5]. There were a few exceptions regarding SA1 [adjusted for 30 LTIs], with elevated HRs for agricultural work companies (HR = 1.48 [1.05–2.07]), for female FMs engaged in fruit arboriculture (HR = 1.61 [1.01–2.57]), and for male crop farmers (HR = 1.35 [1.13–1.60]). There was also a modestly elevated HR for unspecified and mixed farming (HR = 1.16 [1.01–1.33]) and two activities with reduced HRs, namely unspecified small animal farming (HR = 0.63 [0.40–0.99]) and stud farming (HR = 0.51 [0.30–0.88]).

Regarding exposure-response trends [SA4 and SA5], no elevated or reduced HR was found when considering the least exposed group [<median number of years of exposure] [SA4]. By contrast, when considering the highest exposed group [≥median number of years of exposure] [SA5], elevated HRs were observed for fruit arboriculture (HR = 1.52 [1.03–1.83]), dairy farming (HR = 1.41 [1.19–1.66]), and male FMs engaged in agricultural work companies (HR = 2.55 [1.05–6.20]). A modestly elevated HR was also observed with SA5 in viticulture (HR = 1.22 [1.05–1.35]).

An increase of 1 year in engaging in dairy farming, fruit arboriculture, and viticulture translated to an increase of 0.5% (HR = 1.005 [1.001–1.032]), 0.5% (HR = 1.005 [1.002–1.028]), and 0.4% (HR = 1.004 [1.001–1.042]) in the risk of UC, respectively [Supplementary Table S7].

The sensitivity analysis restricted to UC cases identified from 2013 to 2016 [SA6] yielded similar results to the main analysis. There was only one exception, with shellfish farming, that had an elevated HR (,HR = 3.10 [1.47–6.53]) contrary to the main analysis.

### 3.4. IBD risk associated with agricultural activities

Compared with other activities, a negative association [ie, reduced HR] with IBD was observed for stud farming (HR = 0.59 [0.36–0.97]) [Table 2]. By contrast, elevated HRs were found for shellfish farming (HR = 2.12 [1.25–3.59]), fruit arboriculture (HR = 1.52 [1.13–2.52]), and dairy farming (1.30 [1.15–1.47]), and a modestly elevated HR was observed for unspecified and mixed farming (HR = 1.20 [1.04–1.38]). Three activities exhibited sex differences, with more elevated HRs in females than in males, with the exception of viticulture, for which there was only a positive association in males (HR = 1.00 [0.76–1.32] vs HR = 1.20 [1.02–1.42]). Females had HRs 1.72 and 1.64 times more elevated than males for ovine and caprine farming, and training, dressage and riding clubs, respectively.

**Table 2:** Risks of inflammatory bowel disease by agricultural activity, TRACTOR project, France, 2002–2016

Agricultural activity	Sex	*n*	m [%]	HR [95%CI]	*p*	*p*adj
Truck farming, floriculture/flower-growing	Both sexes	43 928	68 [0.16]	1.04 [0.81–1.32]	0.78	0.86
	Female	13 457	15 [0.11]	0.75 [0.45–1.26]	0.28	0.86
	Male	30 471	53 [0.17]	1.13 [0.86–1.49]	0.38	0.86
Fruit arboriculture	Both sexes	25 249	42 [0.17]	**1.52 [1.13–2.52]**	**0.01**	**0.02**
	Female	8033	15 [0.19]	1.42 [0.85–2.37]	0.18	0.85
	Male	17 216	27 [0.16]	1.00 [0.68–1.46]	0.98	0.98
Garden centre/tree nursery	Both sexes	5441	10 [0.18]	1.19 [0.64–2.21]	0.59	0.77
	Female	1443	1 [0.07]	not calculated		
	Male	3998	9 [0.23]	1.41 [0.73–2.71]	0.31	0.77
Crop farming [eg, wheat, corn, and industrial grower]	Both sexes	320 016	440 [0.14]	1.03 [0.93–1.16]	0.56	0.63
	Female	107 138	116 [0.11]	1.07 [0.86–1.33]	0.55	0.63
	Male	212 878	324 [0.15]	1.05 [0.92–1.20]	0.46	0.63
Viticulture	Both sexes	123 669	213 [0.17]	1.13 [0.98–1.30]	0.09	0.28
	Female	43 805	57 [0.13]	1.00 [0.76–1.32]	0.98	0.98
	Male	79 864	156 [0.20]	**1.20 [1.02–1.42]**	**0.03**	**0.05**
Sylviculture/forestry [eg, thinning, pruning]	Both sexes	2180	2 [0.09]	not calculated		
	Female	367	0 [0]	not calculated		
	Male	1813	2 [0.11]	not calculated		
Unspecified specialised farming [eg, herbs, mushrooms]	Both sexes	6688	12 [0.18]	1.19 [0.67–2.10]	0.55	0.70
	Female	2443	4 [0.16]	1.02 [0.38–2.72]	0.98	0.98
	Male	4245	8 [0.19]	1.21 [0.61–2.43]	0.59	0.70
Dairy farming	Both sexes	163 997	321 [0.20]	**1.30 [1.15–1.47]**	**2.2e-5**	**9.8e-5**
	Female	50 814	103 [0.20]	**1.37 [1.10–1.70]**	**5.1e-3**	**7.7e-3**
	Male	113 183	218 [0.19]	**1.25 [1.08–1.44]**	**3.5e-3**	**7.0e-3**
Cow farming	Both sexes	114 103	156 [0.14]	0.88 [0.74–1.03]	0.12	0.27
	Female	33 930	44 [0.13]	0.96 [0.71–1.31]	0.81	0.91
	Male	80 173	112 [0.14]	0.85 [0.70–1.03]	0.10	0.27
Mixed cattle farming	Both sexes	31 576	57 [0.18]	1.19 [0.91–1.55]	0.21	0.46
	Female	8311	11 [0.13]	0.90 [0.49–1.63]	0.73	0.97
	Male	23 265	46 [0.20]	1.27 [0.94–1.70]	0.12	0.35
Ovine and caprine farming	Both sexes	49 316	75 [0.15]	0.96 [0.76–1.21]	0.72	0.81
	Female	17 613	34 [0.19]	1.28 [0.90–1.82]	0.17	0.37
	Male	31 703	41 [0.13]	0.78 [0.57–1.06]	0.11	0.35
Pig farming	Both sexes	13 850	21 [0.15]	0.96 [0.63–1.48]	0.86	0.95
	Female	3981	9 [0.23]	1.53 [0.79–2.97]	0.20	0.73
	Male	9869	12 [0.12]	0.74 [0.42–1.31]	0.30	0.73
Stud farming	Both sexes	17 306	16 [0.09]	**0.59 [0.36–0.97]**	**0.04**	0.25
	Female	7493	6 [0.08]	0.47 [0.21–1.06]	0.07	0.25
	Male	9813	10 [0.10]	0.62 [0.33–1.15]	0.13	0.25
Unspecified large animal farming [eg, ostrich, llama]	Both sexes	3034	2 [0.07]	not calculated		
	Female	1463	1 [0.07]	not calculated		
	Male	1571	1 [0.06]	not calculated		
Poultry and rabbit farming	Both sexes	25 941	46 [0.18]	1.10 [0.82–1.47]	0.53	0.93
	Female	10 229	20 [0.20]	1.23 [0.78–1.92]	0.37	0.93
	Male	15 712	26 [0.17]	0.99 [0.67–1.45]	0.94	0.94
Unspecified small animal farming [eg, frogs, snails, bees]	Both sexes	20 014	24 [0.12]	0.72 [0.48–1.08]	0.12	0.42
	Female	8642	8 [0.09]	0.51 [0.25–1.03]	0.06	0.42
	Male	11 372	16 [0.14]	0.80 [0.49–1.32]	0.39	0.50
Training, dressage, riding clubs	Both sexes	14 988	21 [0.14]	0.76 [0.49–1.16]	0.20	0.45
	Female	6681	13 [0.20]	0.86 [0.49–1.50]	0.59	0.69
	Male	8307	8 [0.10]	**0.50 [0.25–1.00]**	**0.05**	0.44
Shellfish farming [eg, oyster farming, scallop aquaculture]	Both sexes	3823	14 [0.37]	**2.12 [1.25–3.59]**	**5.2e-3**	**0.02**
	Female	735	4 [0.54]	**3.46 [1.29–9.26]**	**0.01**	**0.03**
	Male	3088	10 [0.32]	**1.89 [1.01–3.52]**	**0.05**	0.07
Unspecified and mixed farming [eg, polyculture, mixed farming, diversified farming]	Both sexes	129 801	225 [0.17]	**1.20 [1.04–1.38]**	**0.01**	**0.05**
	Female	40 256	54 [0.13]	1.01 [0.76–1.34]	0.95	0.95
	Male	89 545	171 [0.19]	**1.27 [1.08–1.50]**	**3.5e-3**	**0.03**
Salt works/salt evaporation pond	Both sexes	981	2 [0.20]	not calculated		
	Female	221	0 [0]	not calculated		
	Male	760	2 [0.26]	not calculated		
Wood production [eg, lopping]	Both sexes	11 472	14 [0.12]	0.63 [0.37–1.07]	0.09	0.25
	Female	305	0 [0]	not calculated		
	Male	11 167	14 [0.13]	0.69 [0.41–1.17]	0.17	0.25
Stationary sawmill [eg, edging, trimming, decking, debarking]	Both sexes	796	1 [0.13]	not calculated		
	Female	55	0 [0]	not calculated		
	Male	741	1 [0.14]	not calculated		
Agricultural work companies [eg, pesticide applications, harvest reaping]	Both sexes	15 665	30 [0.19]	1.13 [0.78–1.62]	0.52	0.72
	Female	1891	3 [0.16]	0.92 [0.30–2.87]	0.89	0.89
	Male	13 774	27 [0.20]	1.19 [0.81–1.74]	0.38	0.72
Gardening, landscaping and reforestation companies	Both sexes	49 231	96 [0.20]	0.92 [0.75–1.14]	0.45	0.85
	Female	2568	4 [0.16]	0.79 [0.29–2.11]	0.64	0.85
	Male	46 663	92 [0.20]	1.01 [0.81–1.25]	0.93	0.93
Company representative/authorised representative	Both sexes	1941	3 [0.16]	1.24 [0.40–3.84]	0.72	0.72
	Female	1505	2 [0.13]	not calculated		
	Male	436	1 [0.23]	not calculated		
Rural craftsperson [eg, mason, mechanics]	Both sexes	7699	2 [0.03]	not calculated		
	Female	283	0 [0]	not calculated		
	Male	7416	2 [0.03]	not calculated		

Models are adjusted for sex [for ‘both sexes’ only], age, first year of the farm’s establishment, number of working years, geographical areas, and number of pre-existing medical comorbidities.

HR, hazard ratio; m, number of exposed cases; *n*, exposed population; *p*, *p*-value; *p*adj, *p*-value adjusted using the Benjamini–Hochberg approach. Bold values refer to hazard ratios that do not include unity (one) in their confidence intervals and *p*-values ≤0.05.

All SAs conducted to assess the influence of pre-existing medical comorbidities on the risk of IBD yielded results similar to those of the main analysis [Supplementary Figures S8–S10, Table S6]. There were a few exceptions, with an elevated HR for crop farming (HR = 1.48 [1.32–1.66]) with SA2 [no adjustment on comorbidities], modestly elevated HRs for mixed cattle farming (HR = 1.28 [1.05–1.57]) with SA1 [adjusted for 30 LTIs], and for viticulture (HR = 1.17 [1.01–1.35]) with both SA2 and SA3 [adjusted for mental health issues], as well as reduced HRs for unspecified small animal farming (HR = 0.67 [0.47–0.96]) with SA1 and SA3, and for wood production (HR = 0.58 [0.34–0.99]) with SA2.

Regarding exposure-response trends [SA4 and SA5], a negative association was found when considering the least exposed group [< median number of years of exposure] [SA4] for ovine and caprine farming [HR = 0.59 [0.36–0.99]). There was also an elevated HR for female dairy FMs [HR = 1.30 [1.02–2.40]). By contrast, when considering the highest exposed group [≥median number of years of exposure] [SA5], elevated HRs were found for shellfish farming (HR = 2.06 [1.16–2.34]) and fruit arboriculture (HR = 1.43 [1.11–2.55]). Modestly elevated HRs were also observed with SA5 in crop farming (HR = 1.26 [1.05–1.51]), dairy farming (HR = 1.24 [1.09–1.42]), and males engaged in viticulture (HR = 1.18 [1.03–1.42]), whereas reduced HRs were found for stud farming (HR = 0.54 [0.33–0.88]), wood production (HR = 0.52 [0.29–0.95]), and males engaged in training, dressage, and riding clubs (HR = 0.42 [0.19–0.93]).

An increase of 1 year in performing dairy farming and shellfish farming translated to an increase of 0.9% (HR = 1.009 [1.002–1.043]) and 0.4% (HR = 1.004 [1.002–1.099]) in the risk of IBD, respectively [Supplementary Table S7]. A 1-year increase in engaging in crop farming and fruit arboriculture resulted in an increase of 0.6% (HR = 1.006 [1.002–1.069]) and 0.4% (HR = 1.004 [1.001–1.041]), respectively. Regarding viticulture, a 1-year increase in exposure resulted in an increase of 0.3% in the risk of IBD, but only in males (HR = 1.003 [1.000–1.051]). By contrast, an increase of 1 year in engaging in stud farming translated to a decrease of 0.9% (HR = 0.991 [0.984–0.999]) in the risk of IBD.

The sensitivity analysis restricted to IBD cases identified from 2013 to 2016 [SA6] yielded results similar to those of the main analysis. There were a few exceptions, with a modestly elevated HR for viticulture (HR = 1.25 [1.06–1.47]) and no reduced HR for stud farming (HR = 0.67 [0.37–1.22]) for SA6. In addition, compared with the main analysis, elevated HRs were observed for pig farming (HR = 2.10 [1.04–4.25]), fruit arboriculture (HR = 1.88 [1.06–3.36]), and ovine and caprine farming (HR = 1.82 [1.24–2.68]) in females, and there was an elevated HR in males engaged in agricultural work companies (HR = 1.71 [1.14–2.57]).

## 4. Discussion

This nationwide, population-based, cohort study provides a detailed description of IBD risks among FMs. Some farming activities were found to be more strongly associated with IBD than other activities among the entire French FM workforce population. Elevated HRs were observed for dairy farming, viticulture, fruit arboriculture, shellfish farming, and crop farming. By contrast, reduced HRs were found for stud farming and small animal farming. The results were mostly similar for both UC and CD. The only exceptions were for crop farming and shellfish farming, which were found to have a higher risk of CD but not UC. The same findings were observed in several sensitivity analyses, in particular when examining 1-year increases in engaging in these activities, and also for the highest exposed group when exploring exposure-response trends, which strengthens the validity of our findings.

### 4.1. Prevalence and incidence

The prevalence proportions for IBD were similar to those reported in a German study that used a large, insurance-based cohort, and in a Swedish study that used a population-based register.^7,53^ Consistent with previous work, the prevalence proportions were higher than those reported in North America.^3,5,19,54^ By contrast, the incidence rates reported in the literature were often around four to 10 times smaller than those we found.^5,9,19,47,55^ Our results were also 10 times higher than the EPIMAD French population-based study that used an IBD registry pertaining to 9.1% of the general French population.^56^ The mean annual incidences of adult-onset IBD, CD, and UC from EPIMAD were 13.5, 7.5, and 5.4 per 100 000 inhabitants, respectively. However, our incidence rates were in the range of those previously reported in European countries, with 0.3–22.8 CD cases per 100 000 person-years, 0.6–57.9 UC cases per 100 000 person-years, and 7.7–84.2 IBD cases per 100 000 person-years, respectively.^9,19,47,54–58^

One possible explanation regarding the aforementioned differences in incidence rates could be that some of our identified IBD cases were not truly incidental because we did not have access to health data prior to 2012, nor to hospital discharge data, and because of the administrative nature of the data used. Indeed, LTI declarations are granted after the disease diagnosis, sometimes months or years later. When excluding IBD cases identified in 2012 from our analysis [SA6], the incidence rates were almost two times lower, with 13.4 [11.8–15.1] CD cases per 100 000 person-years, 21.7 [19.5–23.9] UC cases per 100 000 person-years, and 35.1 [32.4–38.1] IBD cases per 100 000 person-years, respectively [Supplementary Figure S11]. The incidence rates for SA6 are in the range of those previously reported in European countries.^9,19,47,54–58^ Comparisons of prevalence and incidence estimates across studies should, however, be interpreted cautiously because they are hampered by differences regarding the data sources, case ascertainment, diagnostic criteria used, study period, sample size, and geographical scope.^53^

### 4.2. Dysbiosis of the gut microbiome

The diversity of farming activities is characterised by a wide variety of exposures to biologic agents and chemicals [agricultural exposome], which in turn influence the microbiome. The gut microbiome is involved in IBD pathogenesis, as a modified microbiota composition is associated with impaired intestinal barrier function and dysregulation of the mucosal immune system.^5,15,33^

Contact with animals during breeding activities is associated with changes in the dermal, nasal, and gut microbiota, as the microbiome of the upper respiratory track is then swallowed.^59^ Most available studies pertain to pig and dairy farmers’ bacteriome. For instance, the same seasonal variability in pig barn bacteriome and in the nasal microbiota of pig breeders was demonstrated.^60^ Regarding gut microbiota, studies showed a lower microbiota species’ diversity and a greater similarity with swine’s gut profile among pig breeders compared with local controls.^61^

Farmers’ gut microbiota differ depending on the type of livestock. For instance, one study showed higher relative abundances of Prevotellaceae and fewer Bacteroidaceae among pig breeders than cattle farmers.^62^ Dairy farmers exhibited a higher richness in the nasal microbiome and a lower burden of *Staphylococcus spp*.^63^ Human faecal resistome is influenced by antibiotic resistome in livestock farms.^64–66^ With only a few studies on dairy farmers, grain industry workers, and sawmill workers, very little is known about the inhalable mycobiome.^67–69^ We did not find any studies regarding the virome.

Even though most studies relate to the pig breeders’ bacteriome, gut dysbiosis seems to be associated with IBD and to be particularly modified in some agricultural occupations, which represent a promising research avenue.

The impact of pesticide exposure on the gut microbiota also starts to be assessed.^70,71^ One study has shown that exposure to pesticides influences the composition of the gut microbiome and can lead to a decrease in gut microbiome alpha diversity as well as changes in microbial community structure.^32^ Another study found that several herbicides, including dicamba, 2,4-D, 2,4-dinitrofluorobenzene, and 2,4,5-trichlorophenoxyacetic acid, may influence gut microbiome homeostasis.^30^

### 4.3. Possible risk factors

The exposure to stressors encountered by individuals in the farming community, specifically those related to farming activities, is heavily influenced by the type of farming being conducted and the potential risk factors associated with it [agricultural exposome]. Despite the presence of common stressors in some farming activities, such as exposure to pesticides or pathogens, the specific nature of the stressor as well as its unique characteristics, such as application method or exposure pathway, can significantly differ.

#### 4.3.1. Sex

Sex differences in prevalence proportions were found, with a female/male ratio of 1.06 and 1.26 for IBD and CD, respectively. By contrast, a male/female ratio of 1.03 was observed for UC. Sex differences in incidence rates were also noted, with a female/male ratio of 1.03 and 1.19 for IBD and CD, respectively. By contrast, a male/female ratio of 1.08 was found for UC. These differences are consistent with the published literature.^72–74^

Sex differences were also observed for all IBD for a few activities [from two to four], in particular for shellfish farming, dairy farming, viticulture, and fruit arboriculture, which all exhibited elevated HRs of IBD. The disparities identified could stem from potential variations in the tasks executed and/or the degree of exposure to environmental factors between females and males, as suggested in other studies.^75,76^ For instance, fewer males tend to implement protective measures or behaviours when handling pesticides, and they tend to use a greater amount of pesticides than females.^76^

These differences could also be possibly associated with potential sex dimorphism [genetics, physiological, and psychological] in IBD, such as differences in the microbiota composition, implications of sex hormones that can influence epithelial and immune dysregulation, or differences in the prevalence of diseases associated with IBD, such as depression.^39,73,74,77–80^ Evidence also suggests that males may be more susceptible to neurotoxic effects from organophosphate pesticides than females,^81^ and a recent study suggests that female spouses of male applicators may be more susceptible to the effects of pesticides in relation to IBD risk.^31^ In addition, IBD phenotype and location seem to exhibit sex-specific differences.^77,78^

#### 4.3.2 Pathogen exposure

Exposure to pathobionts and pathogens [eg, viruses, bacteria, parasites], such as bovine zoonotic pathogens,^26,28^ has been associated with increased risk of IBD, in particular *Mycobacterium avium subspecies paratuberculosis* [MAP],^9–11,15,16,24,25,27,82–84^ and Cryptosporidium.^26,57^ Analogies between CD and Johne’s disease, a cattle and sheep granulomatous chronic enteritis caused by MAP, have been suggested.^16,24,27,83,84^ Studies examining the role of MAP in CD aetiology and pathogenesis yield conflicting evidence, attributed to methodological issues and sample variability.^10,24^ Whereas higher detection of MAP DNA in CD patients has been reported, demonstrating a causal relationship remains challenging since MAP infection could also result from the increased intestinal permeability associated with CD.^9,10,24,27^ Dairy farmers had elevated HRs, possibly due to exposure to MAP and other pathogens through cow manure used as fertiliser,^24–26,83^ inhalation of dust from inert material [pasture and fertiliser],^83^ contaminated water, or vectors.^25,26,28^

Regarding activities involving horses and small animal farming, reduced HRs were observed. One possible explanation may come from the hygiene hypothesis, which posits that childhood exposure to farm animals, which has been associated with lower IBD risk, could increase microbial stimuli and facilitate maturation of the immune system in early life.^9,10,17^

Shellfish farmers had elevated HRs, potentially associated with exposure to pathogens such as bacteria [eg, cyanobacteria] and toxins [eg, microcystins, cylindrospermopsin].^41,85^ Indeed, these pathogens can induce gastrointestinal symptoms, immune system effects, and gut microbiome dysbiosis.^86–89^ However, to our knowledge, no studies have explored this hypothesis so far.

#### 4.3.3. Chemical exposure

The elevated HRs we observed could potentially be related to chemical exposure. For instance, crop farming, viticulture, and fruit arboriculture are characterised by the intensive and frequent use of pesticides. Incidentally, one study found a cluster of higher CD prevalence in the Bordeaux area, which is an important wine-growing region.^90^ The nature, amount, frequency, and mode of application of the pesticides used differ between farming activities, which could potentially explain the difference observed. However, IBD risks related to specific pesticides and chemical compounds were not investigated because these data were not collected by MSA.

Several studies have reported associations between environmental pollutants and changes in the microbiome.^32^ Exposure to chemicals such as aluminum, perfluorooctanoic acid, iron, mercury, and zinc has been associated with a higher risk of IBD, but the levels of evidence are variable.^9,27,30^ Some of these compounds can be found in pesticides. For instance, ziram and zineb contain zinc, iron salt and iron phosphate contain iron, and aluminum monophosphide, aluminum sulphate, and fosetyl-aluminum contain aluminum. However, the exposure levels to metals are considerably lower than in metalworkers [eg, welders].

In human epidemiological studies, higher risks of several autoimmune diseases were associated with pesticide use, in particular rheumatoid arthritis,^35^ and systemic autoimmune diseases such as systemic lupus erythematosus.^33,91^ However, regarding IBD risk related to pesticide exposure, there is a paucity of information, in particular in humans. In a Danish nationwide cohort, early-life environmental exposure to agriculture land use was associated with increased CD risk but not with UC.^92^

A study using animal models reported that the herbicide propyzamide increases inflammation in the small and large intestines.^93^ Propyzamide is a broadly used herbicide for weed control during the production of vegetables, fruits, and ornamental plants.^93,94^ Ten other agricultural-related chemicals were found as potential IBD-promoting chemicals: azinphos-methyl [an organophosphate used in crops, viticulture, and fruit arboriculture], boscalid and tebuconazole [fungicides used in crops, viticulture, and fruit arboriculture], mepanipyrim [a fungicide used in crops and viticulture], oxamy and ethion [insecticides used on crops, fruits, and vegetables], phorate and fenamiphos [insecticides used in plants and soils of various crops], chloranocryl [a contact herbicide], and coumaphos [an organophosphate insecticide used to control livestock insects].^93,94^

In the Agricultural Health Study [AHS], exposure to specific pesticides among male applicators and their spouses was associated with elevated hazards of IBD.^31^ HRs were highest for insecticides solely used in crops [dieldrin, toxaphene, and terbufos], for insecticides used in crops, viticulture, and fruit arboriculture [parathion, DDT], as well as for a herbicide used solely on crops [2,4,5-T].^31,94^ Positive associations were also reported for phorate, terbufos, metolachlor [a herbicide used in crops and fruits], and atrazine [a herbicide used in crops and fruits] only in a subgroup with farm animal contact.^31,94^ By contrast, three insecticides [aluminum phosphide, dichlorvos, and heptachlor], one fungicide [maneb/mancozeb], and one fumigant [carbon disulphide/carbon tetrachloride] used on crops, fruits, vegetables, and viticulture were positively associated with IBD in a specific subgroup that did not have any contact with animals.^31,94^

Air pollution is also sometimes considered a risk factor, but mixed results were reported.^12,14,15,23^ A recent geospatial modelling study investigating paediatric IBD in Canada found that fine particulate matter pollution (incidence rate ratio, IRR = 1.29 [1.11–1.51]), and agricultural application of petroleum oil to orchards and grapes (IRR = 1.14 [1.01–1.27]) were novel risk factors.^95^

Shellfish farmers could be exposed to microplastics and nanoplastics, as reported in several works,^42,96,97^ including a recent French study.^34^ Incidentally, one study reported that farmed mussels had significantly higher microplastic concentrations than wild-caught mussels.^42^ In addition, micro- and nanoplastics provide a protective niche for adsorbed pathogens [eg, toxins, bacteria] and microorganisms.^97^ Micro- and nanoplastics are emerging environmental contaminants that can impair the gut epithelial barrier, induce histological changes, alter intestinal flora, and cause gut microbiota dysbiosis, ultimately leading to IBD.^30,98,99^ Because of their potential effects on human health, micro- and nanoplastics could also cause greater harm to individuals with pre-existing IBD.^98,100^

#### 4.3.4. Geographical factor

FMs with IBD lived more often in the north of France. This finding is consistent with existing literature, as Northern France appears to have one of the highest incidences of IBD in the world, in particular for CD, for which it is a high CD prevalence cluster.^8,90^ Some factors have been suggested to explain this observation, such as the high levels of social deprivation in Northern France,^90^ a North-South gradient,^10^ and low sunshine exposure.^101^ However, one study found that CD incidence rates were negatively related to the percentage of farmers in Northern France.^8^

### 4.4. Strengths and limitations

To our knowledge, this is the first work to comprehensively study IBD risk in the farming population. In addition, IBD risks were estimated for specific agricultural activities and by sex category using large-scale, nationwide, population-based data. These data sources provided a large sample size and the possibility to assess risk variations in farming activity. Findings from this study are important because few epidemiological studies have evaluated IBD and occupational risk factors.^15,27^ In addition, we provided sex-specific risk estimates of IBD, which are lacking for environmental exposures.^78^ This work represents an exploratory study that highlights how routinely collected digital administrative health data can be used as a complementary approach to traditional cohort studies to identify populations at risk, and also to inform health services and health policy.

Several limitations of the study warrant careful consideration and further research. Data from hospital discharges were not available. Because administrative health data were used, only a limited range of confounders was available, with no information on suspected risk factors such as cigarette smoking. Smoking was reported to be a protective factor for UC but a risk factor for CD.^1–3,5,10,12,15–17^ The prevalence of ever-smokers in French farmers is lower than in non-farmers, so the bias introduced by the unavailability of this factor should be limited.^102^ In addition, cattle farming and confined livestock farming have the lowest smoking prevalence among farmers,^102^ so the risk observed in dairy farming should be from origins other than smoking, particularly since the increased risk was observed for both UC and CD.

No information on education [eg, highest educational attainment] was available as this was not collected by MSA. Because education has been associated with some risk factors for IBD, such as cigarette smoking, diet, access to health care, or health-seeking behaviours,^103–106^ adjusting for education in models could help reduce residual confounding. Confounders not available to us, or imperfect measurement of existing covariates in the models, could bias the estimated risks, but the magnitude and direction of this bias cannot be known. To account for a potential confounding effect related to possible unmeasured and unequally geographically distributed risk factors [eg, regional differences], all analyses were adjusted to the farm location. We cannot rule out residual confounding, because the accuracy of some variables was limited. Due to the heterogeneous nature and broad range of tasks performed for a given agricultural practice, the same exposure [same stressors] was assigned to FMs performing the same farming activity [similar exposure group], which may have resulted in a non-differential misclassification of the estimated FM exposures.

The use of LTI declarations for case ascertainment is a limitation because it only allows us to identify FMs under medical care with a LTI, which does not fully represent the real disease incidence. Some FMs with an IBD may not have a LTI declaration if they do not have a treatment for IBD [benign form] or if they did not have surgery related to IBD. The number of FMs in that situation should, however, be limited. According to the National Fund for Health Insurance [CNAM] and a study using the French National Health Data System [SNDS], only 13–14% of IBD patients do not have LTI.^47,51^ These patients can be identified with hospital discharge data, which are not collected by MSA, thus preventing us from identifying them.

Misclassification of other diagnoses as IBD should not exist or be extremely limited. Indeed, LTI declarations are granted by an insurance physician after careful examination of medical documents signed by a general practitioner and/or gastroenterologist, which strengthen the validity of the IBD diagnosis.^47^ In addition, the identification of CD and UC cases was based on previously published and validated algorithms.^47–50^ Validation of all patients with IBD diagnoses through clinical examinations was not possible. It was not possible for us to identify any indeterminate IBD [IBD-U] cases from the study population because we only had access to three-digit ICD-10 codes [eg, K50]. Hence, although we could identify K52 cases [‘other and unspecified noninfective gastroenteritis and colitis’], it was not possible for us to know the K52 subtypes, in particular K52.3, which refer to IBD-U cases.^107^ However, only eight FMs had an LTI for K52 between 2012 and 2016.

Our incidence rates could be higher than they should be because, in a few cases, individuals may have changed their insurance scheme [eg, if someone became a farmer after working in another industrial sector]. In that case, these individuals are registered as new patients under their new insurance scheme [new LTI declaration date], even if they have been suffering from IBD for a long time.^8,108^ In addition, we did not have access to health data before 2012 or hospital data, precluding us from identifying potential prevalent IBD cases [2002–2011 and during follow-up] and excluding them from analysis. Because of the administrative nature of the available data, there was no way of knowing if a disease declaration [LTI] was new [ie, a true incident case occurring during the follow-up] or a renewal of a past disease declaration [ie, a false incident case occurring during the follow-up]. Hence, we cannot rule out the possibility that all identified IBD cases were truly incidental, in particular for the first year of follow-up [2012] because, before 2017, LTIs for IBD were renewed every 2 years.^52^ However, when excluding IBD cases identified in 2012 from the analysis [SA6], the results were similar, and the incidence rates were in the range of those previously reported in European countries.^9,19,47,54–58^ These above-mentioned reasons could potentially explain why our incidence rates were higher than those of the French general population.

Employees/farmworkers [including seasonal and migrant labourers] were not included in our study due to different coding systems and data structures.^43,109^ Employees refer to individuals hired by FMs to work on the farm, helping FMs with their farm work.^43^ The nature of the work carried out by farmworkers depends on the tasks they engage in on the farm, which may sometimes be very different from those of FMs, leading to potentially very disparate exposures compared with FMs. In addition, FMs and employees have different socioeconomic status, experiences, and behaviours, which could influence and bias risk estimation if both populations are not studied separately. Hence, the generalisability of our findings is limited to FMs and may not apply to the entire population at risk, which includes farmworkers, farm families [eg, spouses and children], but also residents living near farms and consumers of farming products [eg, products potentially containing pesticide residues or possibly contaminated with pathogens]. A perspective from this work could be to conduct the same analysis on farmworkers and other populations at risk, such as the AHS study has done with the spouses of male pesticide applicators.^31^ Finally, since agricultural activities and risk/protective factors can differ between countries, the generalisability of some of our findings may be limited. A more detailed discussion regarding the limitations of TRACTOR is described elsewhere.^43^

### 4.5. Public health implications

Findings from this study will be of interest to policy makers, public health practitioners, and IBD researchers. Our results could also have an impact on patients suffering from IBD. Discovering how environmental factors influence the risk of IBD and contribute to its pathogenesis could help IBD-free individuals determine how they might potentially reduce their risk, and it could also help IBD patients experience a milder clinical course, as many unmet therapeutic needs remain.

Indeed, IBD patients make up a high-risk group that could be more vulnerable to adverse health outcomes caused by environmental factors. IBD patients may be subjected to an excessive immune response that could induce or worsen intestinal injury as well as exacerbate IBD symptoms and pathogenesis [eg, disease recurrence] due to their higher gut permeability, which increases the probability of harmful xenobiotics crossing the intestinal barrier compared with the situation in healthy individuals.^98,100^ In addition, some environmental factors can play a role in modulating the clinical course [eg, severity, behaviour, and complications] of both UC and CD.^110–113^ For instance, smoking can act as an aggravating factor in CD patients,^9,10,20,111–114^ and consumption of ultra-processed foods may increase disease severity and recurrence in both UC and CD patients.^115,116^

Gaining a more comprehensive understanding of environmental risk factors, in particular modifiable ones, is therefore essential for prevention, as it may lead to potentially tailored interventions [eg, lowering exposure to risk factors] and treatments [eg, targeting specific pathogens] that address the unique needs of IBD patients. However, determining the prognostic or predictive potential of factors associated with IBD is a challenging endeavour.

Our findings can act as a starting point for identifying potential targets for additional work. Identifying vulnerable populations at risk of IBD and risk/protective factors is crucial for prevention. Further research regarding specific farming activities [eg, dairy farming, shellfish farming, fruit arboriculture, viticulture, and crop farming] and exposures [eg, pesticides, pathogens] is required to identify potential occupational risk factors for IBD and to determine whether additional resources should be allocated and where, and which preventive measures should be implemented.

In conclusion, our findings highlight that different farming activities might have different risks of IBD across the entire FM workforce. IBD may not have a single aetiology but probably results from the concomitant action of multiple causal agents and triggering factors [agricultural/farming exposome], as alluded to by the AHS study, which suggests that the combined exposure to bacterial antigens [from animal contact] and pesticides may result in a synergistic adverse effect on IBD risk.^31^ Our results advocate for additional research to identify and understand the workplace determinants of IBD and to establish and recommend prevention strategies in these settings. Confirmation of our findings in longitudinal studies and in other countries would be valuable. Exposure to MAP, Cryptosporidium, toxins, micro- and nanoplastics, and pesticides represent promising research hypotheses/avenues that deserve additional research in population-based studies, in particular to assess causal inferences.

## Supplementary Data

Supplementary data are available at *ECCO-JCC* online.

jjae050_suppl_Supplementary_Figures_S1-S11_Tables_S1-S7

## Data Availability

The data [participant data, data dictionary, and other related documents] that support the findings of this study are not publicly available. A reasonable request to the Mutualité Sociale Agricole [MSA] can be made, but restrictions apply to the availability of these data due to the individual and medical nature of the data, which requires approval from both the MSA and the French independent administrative authority protecting privacy and personal data [CNIL]. Further information is available from the corresponding author upon request.
