# Supplementary material for: Farming Activities and Risk of Inflammatory Bowel Disease: A French Nationwide Population-based Cohort Study
Source: J Crohns Colitis. 2024 Apr 12;18(9):1415–29. doi: 10.1093/ecco-jcc/jjae050 (PMC11369074; doi:10.1093/ecco-jcc/jjae050)
Supplement: jjae050_suppl_Supplementary_Figures_S1-S11_Tables_S1-S7 [file jjae050_suppl_supplementary_figures_s1-s11_tables_s1-s7.docx]

**Supplemental material**

**Title**

Farming activities and risk of inflammatory bowel disease: a French nationwide population-based cohort study

**Authors**

Pascal Petit, Ariane Leroyer, Sylvain Chamot, Mathurin Fumery, Vincent Bonneterre

**Table of contents**

[Table S1: Characteristics of the study population, TRACTOR project, France, 2002-2016 2](#_Toc161127090)

[Table S2: Risks of Crohn’s disease by agricultural activity, TRACTOR project, France, 2002-2016 4](#_Toc161127091)

[Table S3: Risks of ulcerative colitis by agricultural activity, TRACTOR project, France, 2002-2016 5](#_Toc161127092)

[Table S4: Farming activities and risk of Crohn’s - comparison of the main analysis with sensitivity analyses, TRACTOR project, France, 2002-2016 6](#_Toc161127093)

[Table S5: Farming activities and risk of ulcerative colitis - comparison of the main analysis with sensitivity analyses, TRACTOR project, France, 2002-2016 7](#_Toc161127094)

[Table S6: Farming activities and risk of inflammatory bowel disease - comparison of the main analysis with sensitivity analyses, TRACTOR project, France, 2002-2016 8](#_Toc161127095)

[Table S7: Hazard ratio for a one-year exposure increase by agricultural activity and IBD 9](#_Toc161127096)

[STROBE Statement Checklist 10](#_Toc161127097)

[Figure S1: Directed acyclic graph for variable selection 11](#_Toc161127098)

[Figure S2: Farming activities and risk of Crohn’s disease among all farm managers - comparison of the main analysis with sensitivity 13](#_Toc161127099)

[Figure S3: Farming activities and risk of Crohn’s disease among female farm managers - comparison of the main analysis with sensitivity analyses 14](#_Toc161127100)

[Figure S4: Farming activities and risk of Crohn’s disease among male farm managers - comparison of the main analysis with sensitivity analyses 15](#_Toc161127101)

[Figure S5: Farming activities and risk of ulcerative colitis among all farm managers - comparison of the main analysis with sensitivity 16](#_Toc161127102)

[Figure S6: Farming activities and risk of ulcerative colitis among female farm managers - comparison of the main analysis with sensitivity analyses 17](#_Toc161127103)

[Figure S7: Farming activities and risk of ulcerative colitis among male farm managers - comparison of the main analysis with sensitivity analyses 18](#_Toc161127104)

[Figure S8: Farming activities and risk of inflammatory bowel disease among all farm managers - comparison of the main analysis with sensitivity 19](#_Toc161127105)

[Figure S9: Farming activities and risk of inflammatory bowel disease among female farm managers - comparison of the main analysis with sensitivity analyses 20](#_Toc161127106)

[Figure S10: Farming activities and risk of inflammatory bowel disease among male farm managers - comparison of the main analysis with sensitivity analyses 21](#_Toc161127107)

[Figure S11: Incidence rate per year and time period for each IBD type 22](#_Toc161127108)

# **Table S1**: Characteristics of the study population, TRACTOR project, France, 2002-2016

|  | **Crohn’s disease** |  | **Ulcerative colitis** |  | **IBD** |  |
| --- | --- | --- | --- | --- | --- | --- |
|  | **FM without**  **(n = 1087857)** | **FM with**  **(n = 704)** | **FM without**  **(n = 1087513)** | **FM with**  **(n = 1048)** | **FM without**  **(n = 1086809)** | **FM with**  **(n = 1752)** |
| **Main characteristics** | n (%) | n (%) | n (%) | n (%) | n (%) | n (%) |
| **Sex** |  |  |  |  |  |  |
| Male | 750808 (69.0) | 490 (69.6) | 750517 (69.0) | 781 (74.5) | 750027 (69.0) | 1271 (72.5) |
| Female | 337049 (31.0) | 214 (30.4) | 336996 (31.0) | 267 (25.5) | 336782 (31.0) | 481 (27.5) |
| **Age (years)** |  |  |  |  |  |  |
| Mean (SD) | 46.6 (14.1) | 41.4 (11.6) | 46.6 (14.1) | 43.9 (11.4) | 46.6 (14.1) | 42.9 (11.6) |
| **Family status** |  |  |  |  |  |  |
| Single | 468705 (43.1) | 305 (43.3) | 468620 (43.1) | 390 (37.2) | 468315 (43.1) | 695 (39.7) |
| As a couple | 619152 (56.9) | 399 (56.7) | 618893 (56.9) | 658 (62.8) | 618494 (56.9) | 1057 (60.3) |
| **First year of the farm’s establishment** |  |  |  |  |  |  |
| Median (IQR) | 1994 (39.0) | 1995 (39.8) | 1994 (39.0) | 1993 (37.0) | 1994 (39.0) | 1993 (38.0) |
| **Farm surface (expressed in hectares)** |  |  |  |  |  |  |
| Median (IQR) | 1.64 (13.9) | 3.41 (16.3) | 1.64 (13.9) | 3.25 (16.2) | 1.64 (13.9) | 3.34 (16.4) |
| **Farm location (region)** |  |  |  |  |  |  |
| Auvergne-Rhône-Alpes | 117383 (10.8) | 45 (6.39) | 117348 (10.8) | 80 (7.63) | 117303 (10.8) | 125 (7.13) |
| Bourgogne-Franche-Comté | 65824 (6.05) | 40 (5.68) | 65798 (6.05) | 66 (6.30) | 65758 (6.05) | 106 (6.05) |
| Bretagne | 81247 (7.47) | 55 (7.81) | 81212 (7.47) | 90 (8.59) | 81157 (7.47) | 145 (8.28) |
| Centre - Val de Loire | 49792 (4.58) | 42 (5.97) | 49780 (4.58) | 54 (5.15) | 49738 (4.58) | 96 (5.48) |
| Corse | 5321 (0.49) | 5 (0.71) | 5322 (0.49) | 4 (0.38) | 5317 (0.49) | 9 (0.51) |
| Grand Est | 81677 (7.51) | 77 (10.9) | 81655 (7.51) | 99 (9.45) | 81578 (7.51) | 176 (10.0) |
| Hauts-de-France | 48312 (4.44) | 86 (12.2) | 48334 (4.44) | 64 (6.11) | 48248 (4.44) | 150 (8.56) |
| Île-de-France | 14073 (1.29) | 11 (1.56) | 14065 (1.29) | 19 (1.81) | 14054 (1.29) | 30 (1.71) |
| Normandie | 80587 (7.41) | 51 (7.24) | 80567 (7.41) | 71 (6.77) | 80516 (7.41) | 122 (6.96) |
| Nouvelle-Aquitaine | 180289 (16.6) | 94 (13.4) | 180236 (16.6) | 147 (14.0) | 180142 (16.6) | 241 (13.8) |
| Occitanie | 166747 (15.3) | 78 (11.1) | 166668 (15.3) | 157 (15.0) | 166590 (15.3) | 235 (13.4) |
| Provence-Alpes-Côte d'Azur | 113978 (10.5) | 75 (10.7) | 113944 (10.5) | 109 (10.4) | 113869 (10.5) | 184 (10.5) |
| Pays de la Loire | 82627 (7.60) | 45 (6.39) | 82584 (7.59) | 88 (8.40) | 82539 (7.59) | 133 (7.59) |
| **Number of farms** |  |  |  |  |  |  |
| 1 farm | 1061431 (97.6) | 679 (96.4) | 1061090 (97.6) | 1020 (97.3) | 1060411 (97.6) | 1699 (97) |
| >1 farm | 26426 (2.43) | 25 (3.55) | 26423 (2.43) | 28 (2.67) | 26398 (2.43) | 53 (3.03) |
| **Farm type (farm clustering)** |  |  |  |  |  |  |
| Individual farm | 756179 (69.5) | 409 (58.1) | 755948 (69.5) | 640 (61.1) | 755539 (69.5) | 1049 (59.9) |
| Farm with work partners | 331678 (30.5) | 295 (41.9) | 331565 (30.5) | 408 (38.9) | 331270 (30.5) | 703 (40.1) |
| **Partner work status** |  |  |  |  |  |  |
| Perform task to help farm manager | 128148 (11.8) | 103 (14.6) | 128074 (11.8) | 177 (16.9) | 127971 (11.8) | 280 (16.0) |
| Do not perform task to help farm manager | 959709 (88.2) | 601 (85.4) | 959439 (88.2) | 871 (83.1) | 958838 (88.2) | 1472 (84.0) |
| **Number of associates** |  |  |  |  |  |  |
| 0 | 835748 (76.8) | 484 (68.7) | 835495 (76.8) | 737 (70.3) | 835011 (76.8) | 1221 (69.7) |
| ≥ 1 | 252109 (23.2) | 220 (31.2) | 252018 (23.2) | 311 (29.7) | 251798 (23.2) | 531 (30.3) |
| **Secondary activity** |  |  |  |  |  |  |
| No secondary activity | 686061 (63.1) | 529 (75.1) | 685744 (63.1) | 846 (80.7) | 685215 (63.0) | 1375 (78.5) |
| At least one secondary activity | 401796 (36.9) | 175 (24.9) | 401769 (36.9) | 202 (19.3) | 401594 (37.0) | 377 (21.5) |
| **Lack of job security** |  |  |  |  |  |  |
| Has never been unemployed during the observation period | 1085742 (99.8) | 701 (99.6) | 1085397 (99.8) | 1046 (99.8) | 1084696 (99.8) | 1747 (99.7) |
| Has been unemployed during the observation period | 2115 (0.19) | 3 (0.43) | 2116 (0.20) | 2 (0.19) | 2113 (0.19) | 5 (0.29) |
| **Median yearly insurance premium (euros)** |  |  |  |  |  |  |
| Median (IQR) | 5158 (40811) | 7931 (48985) | 5156 (40811) | 8485 (47603) | 5153 (40804) | 8282 (48485) |
| **Employees** |  |  |  |  |  |  |
| No employee | 789721 (72.6) | 423 (60.1) | 789514 (72.6) | 630 (60.1) | 789091 (72.6) | 1053 (60.1) |
| At least one employee | 298136 (27.4) | 281 (39.9) | 297999 (27.4) | 418 (39.9) | 297718 (27.4) | 699 (39.9) |
| **Work status** |  |  |  |  |  |  |
| Working as a farm manager | 820095 (75.4) | 652 (92.6) | 819781 (75.4) | 966 (92.2) | 819129 (75.4) | 1618 (92.4) |
| Working as a solidarity contributor* | 267762 (24.6) | 52 (7.39) | 267732 (24.6) | 82 (7.82) | 267680 (24.6) | 134 (7.65) |
| **Pre-existing disability** |  |  |  |  |  |  |
| Did not become disabled before the first LTI declaration for IBD | 1083448 (99.6) | 694 (98.6) | 1083110 (99.6) | 1032 (98.5) | 1082416 (99.6) | 1726 (98.5) |
| Became disabled before the first LTI declaration for IBD | 4409 (0.41) | 10 (1.42) | 4403 (0.41) | 16 (1.53) | 4393 (0.40) | 26 (1.48) |
| **Number of pre-existing comorbidities** |  |  |  |  |  |  |
| 0 comorbidity before the first LTI declaration for IBD | 706019 (64.9) | 341 (48.6) | 705796 (64.9) | 684 (65.3) | 706426 (65.0) | 1190 (67.9) |
| 1 comorbidity before the first LTI declaration for IBD | 214308 (19.7) | 237 (33.7) | 214240 (19.7) | 256 (24.4) | 214101 (19.7) | 382 (21.7) |
| >1 comorbidity before the first LTI declaration for IBD | 167530 (15.4) | 125 (17.8) | 167477 (15.4) | 108 (10.3) | 166282 (15.3) | 180 (10.3) |

Note: FM: farm manager, IBD: inflammatory bowel diseases, IQR: interquartile range, SD: arithmetic standard deviation.

*FMs that farm on a small surface (<12.5 ha) or that works less than 1200 h/year.

# **Table S2**: Risks of Crohn’s disease by agricultural activity, TRACTOR project, France, 2002-2016

| **Agricultural activity** | **Sex** | **n** | **m (%)** | **HR [95%CI]** | **p** | **padj** |
| --- | --- | --- | --- | --- | --- | --- |
| Truck farming, floriculture/flower-growing | Both sexes | 43968 | 28 (0.06) | 1.03 [0.71-1.51] | 0.86 | 0.86 |
|  | Female | 13464 | 8 (0.06) | 0.87 [0.43-1.76] | 0.70 | 0.86 |
|  | Male | 30504 | 20 (0.07) | 1.09 [0.69-1.70] | 0.72 | 0.86 |
| Fruit arboriculture | Both sexes | 25275 | 16 (0.06) | **1.17 [1.04-1.76]** | **0.04** | **0.05** |
|  | Female | 8041 | 7 (0.09) | **1.49 [1.10-3.17]** | **0.01** | **0.03** |
|  | Male | 17234 | 9 (0.05) | 1.00 [0.45-1.68] | 0.68 | 0.88 |
| Garden center/tree nursery | Both sexes | 5447 | 4 (0.07) | 1.16 [0.44-3.10] | 0.77 | 0.77 |
|  | Female | 1443 | 1 (0.07) | not calculated |  |  |
|  | Male | 4004 | 3 (0.07) | 1.20 [0.38-3.72] | 0.76 | 0.77 |
| Crop farming  (e.g., wheat, corn, and industrial grower) | Both sexes | 320263 | 193 (0.06) | **1.26 [1.06-1.49]** | **9.0e-3** | **0.04** |
|  | Female | 107200 | 54 (0.05) | 1.22 [0.89-1.68] | 0.22 | 0.49 |
|  | Male | 213063 | 139 (0.07) | **1.31 [1.07-1.60]** | **9.9e-3** | **0.04** |
| Viticulture | Both sexes | 123796 | 86 (0.07) | **1.16 [1.00-1.45]** | 0.08 | 0.15 |
|  | Female | 43835 | 27 (0.06) | 1.10 [0.73-1.65] | 0.65 | 0.78 |
|  | Male | 79961 | 59 (0.07) | **1.19 [1.04-1.58]** | **0.01** | **0.04** |
| Sylviculture/forestry  (e.g., thinning, pruning) | Both sexes | 2180 | 2 (0.09) | not calculated |  |  |
|  | Female | 367 | 0 (0) | not calculated |  |  |
|  | Male | 1813 | 2 (0.11) | not calculated |  |  |
| Unspecified specialized farming  (e.g., herbs, mushrooms) | Both sexes | 6692 | 8 (0.12) | 1.87 [0.93-3.76] | 0.08 | 0.23 |
|  | Female | 2445 | 2 (0.08) | not calculated |  |  |
|  | Male | 4247 | 6 (0.14) | **2.30 [1.03-5.15]** | **0.04** | **0.23** |
| Dairy farming | Both sexes | 164204 | 114 (0.07) | **1.22 [1.10-1.39]** | **3.5e-6** | **2.4e-5** |
|  | Female | 50882 | 35 (0.07) | **1.34 [1.14-1.69]** | **6.1e-5** | **8.9e-5** |
|  | Male | 113322 | 79 (0.07) | **1.14 [1.04-1.28]** | **0.02** | **0.04** |
| Cow farming | Both sexes | 114202 | 57 (0.05) | 0.80 [0.61-1.05] | 0.11 | 0.27 |
|  | Female | 33954 | 20 (0.06) | 1.00 [0.63-1.58] | 0.99 | 0.99 |
|  | Male | 80248 | 37 (0.05) | 0.72 [0.52-1.01] | 0.06 | 0.27 |
| Mixed cattle farming | Both sexes | 31615 | 18 (0.06) | 0.94 [0.59-1.50] | 0.80 | 0.97 |
|  | Female | 8318 | 4 (0.05) | 0.74 [0.28-2.00] | 0.56 | 0.97 |
|  | Male | 23297 | 14 (0.06) | 1.01 [0.59-1.72] | 0.97 | 0.97 |
| Ovine and caprine farming | Both sexes | 49360 | 31 (0.06) | 0.95 [0.66-1.36] | 0.78 | 0.81 |
|  | Female | 17629 | 18 (0.10) | 1.48 [0.91-2.40] | 0.12 | 0.35 |
|  | Male | 31731 | 13 (0.04) | 0.62 [0.36-1.08] | 0.09 | 0.35 |
| Pig farming | Both sexes | 13862 | 9 (0.06) | 1.02 [0.53-1.97] | 0.95 | 0.95 |
|  | Female | 3986 | 4 (0.10) | 1.52 [0.57-4.09] | 0.41 | 0.73 |
|  | Male | 9876 | 5 (0.05) | 0.80 [0.33-1.94] | 0.62 | 0.94 |
| Stud farming | Both sexes | 17315 | 7 (0.04) | 0.61 [0.29-1.28] | 0.19 | 0.25 |
|  | Female | 7496 | 3 (0.04) | 0.49 [0.16-1.53] | 0.22 | 0.25 |
|  | Male | 9819 | 4 (0.04) | 0.64 [0.24-1.70] | 0.37 | 0.37 |
| Unspecified large animal farming  (e.g., ostrich, llama) | Both sexes | 3034 | 2 (0.07) | not calculated |  |  |
|  | Female | 1463 | 1 (0.07) | not calculated |  |  |
|  | Male | 1571 | 1 (0.06) | not calculated |  |  |
| Poultry and rabbit farming | Both sexes | 25971 | 16 (0.06) | 0.90 [0.55-1.48] | 0.68 | 0.93 |
|  | Female | 10242 | 7 (0.07) | 0.92 [0.43-1.95] | 0.82 | 0.93 |
|  | Male | 15729 | 9 (0.06) | 0.86 [0.44-1.65] | 0.64 | 0.93 |
| Unspecified small animal farming  (e.g., frogs, snails, bees) | Both sexes | 20028 | 10 (0.05) | 0.69 [0.37-1.29] | 0.25 | 0.42 |
|  | Female | 8646 | 4 (0.05) | 0.52 [0.19-1.41] | 0.20 | 0.42 |
|  | Male | 11382 | 6 (0.05) | 0.75 [0.33-1.67] | 0.48 | 0.54 |
| Training, dressage, riding clubs | Both sexes | 14999 | 10 (0.07) | 0.78 [0.42-1.46] | 0.44 | 0.66 |
|  | Female | 6687 | 7 (0.11) | 0.89 [0.42-1.92] | 0.77 | 0.77 |
|  | Male | 8312 | 3 (0.04) | 0.45 [0.14-1.39] | 0.16 | 0.45 |
| Shellfish farming  (e.g., oyster farming, scallop aquaculture) | Both sexes | 3830 | 7 (0.18) | **2.51 [1.19-5.30]** | **0.02** | **0.03** |
|  | Female | 736 | 3 (0.41) | **5.48 [1.75-17.2]** | **3.5e-3** | **0.02** |
|  | Male | 3094 | 4 (0.13) | 1.82 [0.68-4.89] | 0.23 | 0.23 |
| Unspecified and mixed farming  (e.g., polyculture, mixed farming, diversified farming) | Both sexes | 129931 | 95 (0.07) | **1.27 [1.02-1.58]** | **0.03** | 0.07 |
|  | Female | 40283 | 27 (0.07) | 1.16 [0.78-1.74] | 0.46 | 0.59 |
|  | Male | 89648 | 68 (0.08) | **1.33 [1.03-1.72]** | **0.03** | 0.07 |
| Salt works/salt evaporation pond | Both sexes | 983 | 0 (0) | not calculated |  |  |
|  | Female | 221 | 0 (0) | not calculated |  |  |
|  | Male | 762 | 0 (0) | not calculated |  |  |
| Wood production  (e.g., lopping) | Both sexes | 11482 | 4 (0.03) | 0.43 [0.16-1.15] | 0.09 | 0.25 |
|  | Female | 305 | 0 (0) | not calculated |  |  |
|  | Male | 11177 | 4 (0.04) | 0.47 [0.17-1.25] | 0.13 | 0.25 |
| Stationary sawmill  (e.g., edging, trimming, decking, debarking) | Both sexes | 797 | 0 (0) | not calculated |  |  |
|  | Female | 55 | 0 (0) | not calculated |  |  |
|  | Male | 742 | 0 (0) | not calculated |  |  |
| Agricultural work companies  (e.g., pesticide applications, harvest reaping) | Both sexes | 15686 | 9 (0.06) | 0.79 [0.41-1.52] | 0.47 | 0.72 |
|  | Female | 1893 | 1 (0.05) | not calculated |  |  |
|  | Male | 13793 | 8 (0.06) | 0.84 [0.42-1.68] | 0.62 | 0.72 |
| Gardening, landscaping and reforestation companies | Both sexes | 49283 | 44 (0.09) | 0.98 [0.71-1.34] | 0.88 | 0.93 |
|  | Female | 2572 | 0 (0) | not calculated |  |  |
|  | Male | 46711 | 44 (0.09) | 1.13 [0.82-1.56] | 0.44 | 0.85 |
| Company representative/authorized representative | Both sexes | 1944 | 0 (0) | not calculated |  |  |
|  | Female | 1507 | 0 (0) | not calculated |  |  |
|  | Male | 437 | 0 (0) | not calculated |  |  |
| Rural craftsperson  (e.g., mason, mechanics) | Both sexes | 7700 | 1 (0.01) | not calculated |  |  |
|  | Female | 283 | 0 (0) | not calculated |  |  |
|  | Male | 7417 | 1 (0.01) | not calculated |  |  |

*Note*: HR: hazard ratio, m: number of exposed cases, n: exposed population, p: p-value, padj: p-value adjusted using the Benjamini-Hochberg approach. Models are adjusted for sex (for “both sexes” only), age, first year of the farm’s establishment, number of working years, geographical areas, and number of pre-existing medical comorbidities.

# **Table S3**: Risks of ulcerative colitis by agricultural activity, TRACTOR project, France, 2002-2016

| **Agricultural activity** | **Sex** | **n** | **m (%)** | **HR [95%CI]** | **p** | **padj** |
| --- | --- | --- | --- | --- | --- | --- |
| Truck farming, floriculture/flower-growing | Both sexes | 43956 | 40 (0.09) | 1.04 [0.76-1.42] | 0.82 | 0.86 |
|  | Female | 13465 | 7 (0.05) | 0.65 [0.31-1.38] | 0.27 | 0.86 |
|  | Male | 30491 | 33 (0.11) | 1.16 [0.82-1.65] | 0.39 | 0.86 |
| Fruit arboriculture | Both sexes | 25265 | 26 (0.10) | **1.45 [1.01-2.70]** | **0.05** | 0.12 |
|  | Female | 8040 | 8 (0.10) | 1.36 [0.68-2.76] | 0.39 | 0.85 |
|  | Male | 17225 | 18 (0.10) | 1.07 [0.67-1.71] | 0.76 | 0.88 |
| Garden center/tree nursery | Both sexes | 5445 | 6 (0.11) | 1.21 [0.54-2.69] | 0.65 | 0.77 |
|  | Female | 1444 | 0 (0) | not calculated |  |  |
|  | Male | 4001 | 6 (0.15) | 1.55 [0.69-3.45] | 0.29 | 0.77 |
| Crop farming  (e.g., wheat, corn, and industrial grower) | Both sexes | 320209 | 247 (0.08) | 0.90 [0.78-1.05] | 0.17 | 0.49 |
|  | Female | 107192 | 62 (0.06) | 0.96 [0.71-1.29] | 0.77 | 0.77 |
|  | Male | 213017 | 185 (0.09) | 0.91 [0.77-1.08] | 0.27 | 0.49 |
| Viticulture | Both sexes | 123755 | 127 (0.10) | **1.12 [1.01-1.28]** | **0.04** | **0.05** |
|  | Female | 43832 | 30 (0.07) | 0.93 [0.63-1.35] | 0.69 | 0.78 |
|  | Male | 79923 | 97 (0.12) | **1.20 [1.04-1.49]** | **0.01** | **0.04** |
| Sylviculture/forestry  (e.g., thinning, pruning) | Both sexes | 2182 | 0 (0) | not calculated |  |  |
|  | Female | 367 | 0 (0) | not calculated |  |  |
|  | Male | 1815 | 0 (0) | not calculated |  |  |
| Unspecified specialized farming  (e.g., herbs, mushrooms) | Both sexes | 6696 | 4 (0.06) | 0.69 [0.26-1.83] | 0.45 | 0.70 |
|  | Female | 2445 | 2 (0.08) | not calculated |  |  |
|  | Male | 4251 | 2 (0.05) | not calculated |  |  |
| Dairy farming | Both sexes | 164111 | 207 (0.13) | **1.46 [1.25-1.70]** | **1.6e-6** | **1.5e-5** |
|  | Female | 50849 | 68 (0.13) | **1.74 [1.32-2.29]** | **8.9e-5** | **2.7e-4** |
|  | Male | 113262 | 139 (0.12) | **1.32 [1.09-1.58]** | **3.9e-3** | **7.0e-3** |
| Cow farming | Both sexes | 114160 | 99 (0.09) | 0.93 [0.76-1.15] | 0.50 | 0.81 |
|  | Female | 33950 | 24 (0.07) | 0.94 [0.62-1.43] | 0.77 | 0.91 |
|  | Male | 80210 | 75 (0.09) | 0.93 [0.73-1.18] | 0.54 | 0.81 |
| Mixed cattle farming | Both sexes | 31594 | 39 (0.12) | 1.35 [0.98-1.86] | 0.07 | 0.29 |
|  | Female | 8315 | 7 (0.08) | 1.03 [0.48-2.17] | 0.95 | 0.97 |
|  | Male | 23279 | 32 (0.14) | **1.43 [1.00-2.04]** | **0.05** | 0.29 |
| Ovine and caprine farming | Both sexes | 49347 | 44 (0.09) | 0.96 [0.71-1.30] | 0.81 | 0.81 |
|  | Female | 17631 | 16 (0.09) | 1.11 [0.67-1.85] | 0.68 | 0.81 |
|  | Male | 31716 | 28 (0.09) | 0.88 [0.60-1.28] | 0.50 | 0.81 |
| Pig farming | Both sexes | 13859 | 12 (0.09) | 0.92 [0.52-1.63] | 0.79 | 0.95 |
|  | Female | 3985 | 5 (0.13) | 1.56 [0.64-3.77] | 0.33 | 0.73 |
|  | Male | 9874 | 7 (0.07) | 0.70 [0.34-1.48] | 0.36 | 0.73 |
| Stud farming | Both sexes | 17313 | 9 (0.05) | 0.58 [0.30-1.11] | 0.10 | 0.25 |
|  | Female | 7496 | 3 (0.04) | 0.46 [0.15-1.43] | 0.18 | 0.25 |
|  | Male | 9817 | 6 (0.06) | 0.61 [0.27-1.36] | 0.23 | 0.25 |
| Unspecified large animal farming  (e.g., ostrich, llama) | Both sexes | 3036 | 0 (0) | not calculated |  |  |
|  | Female | 1464 | 0 (0) | not calculated |  |  |
|  | Male | 1572 | 0 (0) | not calculated |  |  |
| Poultry and rabbit farming | Both sexes | 25957 | 30 (0.12) | 1.25 [0.87-1.80] | 0.23 | 0.93 |
|  | Female | 10236 | 13 (0.13) | 1.50 [0.86-2.63] | 0.15 | 0.93 |
|  | Male | 15721 | 17 (0.11) | 1.07 [0.66-1.74] | 0.77 | 0.93 |
| Unspecified small animal farming  (e.g., frogs, snails, bees) | Both sexes | 20024 | 14 (0.07) | 0.75 [0.44-1.27] | 0.28 | 0.42 |
|  | Female | 8646 | 4 (0.05) | 0.50 [0.19-1.34] | 0.17 | 0.42 |
|  | Male | 11378 | 10 (0.09) | 0.85 [0.45-1.58] | 0.60 | 0.60 |
| Training, dressage, riding clubs | Both sexes | 14998 | 11 (0.07) | 0.72 [0.40-1.31] | 0.29 | 0.52 |
|  | Female | 6688 | 6 (0.09) | 0.81 [0.36-1.84] | 0.61 | 0.69 |
|  | Male | 8310 | 5 (0.06) | 0.54 [0.22-1.29] | 0.16 | 0.45 |
| Shellfish farming  (e.g., oyster farming, scallop aquaculture) | Both sexes | 3830 | 7 (0.18) | 1.84 [0.87-3.87] | 0.11 | 0.13 |
|  | Female | 738 | 1 (0.14) | not calculated |  |  |
|  | Male | 3092 | 6 (0.19) | 1.93 [0.87-4.33] | 0.11 | 0.13 |
| Unspecified and mixed farming  (e.g., polyculture, mixed farming, diversified farming) | Both sexes | 129896 | 130 (0.10) | 1.15 [0.96-1.38] | 0.14 | 0.21 |
|  | Female | 40283 | 27 (0.07) | 0.89 [0.60-1.33] | 0.57 | 0.65 |
|  | Male | 89613 | 103 (0.12) | **1.24 [1.01-1.52]** | **0.04** | 0.08 |
| Salt works/salt evaporation pond | Both sexes | 981 | 2 (0.20) | not calculated |  |  |
|  | Female | 221 | 0 (0) | not calculated |  |  |
|  | Male | 760 | 2 (0.26) | not calculated |  |  |
| Wood production  (e.g., lopping) | Both sexes | 11476 | 10 (0.09) | 0.79 [0.42-1.47] | 0.46 | 0.55 |
|  | Female | 305 | 0 (0) | not calculated |  |  |
|  | Male | 11171 | 10 (0.09) | 0.85 [0.46-1.59] | 0.61 | 0.61 |
| Stationary sawmill  (e.g., edging, trimming, decking, debarking) | Both sexes | 796 | 1 (0.13) | not calculated |  |  |
|  | Female | 55 | 0 (0) | not calculated |  |  |
|  | Male | 741 | 1 (0.14) | not calculated |  |  |
| Agricultural work companies  (e.g., pesticide applications, harvest reaping) | Both sexes | 15674 | 21 (0.13) | 1.38 [0.90-2.13] | 0.14 | 0.50 |
|  | Female | 1892 | 2 (0.11) | not calculated |  |  |
|  | Male | 13782 | 19 (0.14) | 1.44 [0.91-2.27] | 0.12 | 0.50 |
| Gardening, landscaping and reforestation companies | Both sexes | 49275 | 52 (0.11) | 0.88 [0.66-1.18] | 0.40 | 0.85 |
|  | Female | 2568 | 4 (0.16) | 1.58 [0.59-4.27] | 0.36 | 0.85 |
|  | Male | 46707 | 48 (0.10) | 0.92 [0.68-1.24] | 0.57 | 0.85 |
| Company representative/authorized representative | Both sexes | 1941 | 3 (0.16) | 2.30 [0.74-7.15] | 0.15 | 0.30 |
|  | Female | 1505 | 2 (0.13) | not calculated |  |  |
|  | Male | 436 | 1 (0.23) | not calculated |  |  |
| Rural craftsperson  (e.g., mason, mechanics) | Both sexes | 7700 | 1 (0.01) | not calculated |  |  |
|  | Female | 283 | 0 (0) | not calculated |  |  |
|  | Male | 7417 | 1 (0.01) | not calculated |  |  |

*Note*: HR: hazard ratio, m: number of exposed cases, n: exposed population, p: p-value, padj: p-value adjusted using the Benjamini-Hochberg approach. Models are adjusted for sex (for “both sexes” only), age, first year of the farm’s establishment, number of working years, geographical areas, and number of pre-existing medical comorbidities.

# **Table S4**: Farming activities and risk of Crohn’s - comparison of the main analysis with sensitivity analyses, TRACTOR project, France, 2002-2016

| **Agricultural activity** | **Sex** | **MA**  HR [95%CI] | **SA1**  HR [95%CI] | **SA2**  HR [95%CI] | **SA3**  HR [95%CI] | **SA4**  HR [95%CI] | **SA5**  HR [95%CI] | **SA6**  HR [95%CI] |
| --- | --- | --- | --- | --- | --- | --- | --- | --- |
| Truck farming, floriculture/flower-growing | Both sexes | 1.03 [0.71-1.51] | 0.95 [0.75-1.19] | 0.98 [0.67-1.43] | 1.02 [0.70-1.50] | 0.94 [0.30-2.97] | 0.96 [0.64-1.44] | 1.21 [0.77-1.89] |
|  | Female | 0.87 [0.43-1.76] | 0.77 [0.50-1.21] | 0.85 [0.42-1.73] | 0.85 [0.42-1.72] | NC | 0.88 [0.44-1.80] | 1.01 [0.41-2.46] |
|  | Male | 1.09 [0.69-1.70] | 1.02 [0.78-1.33] | 1.02 [0.65-1.59] | 1.08 [0.69-1.69] | 1.18 [0.37-3.72] | 0.98 [0.61-1.60] | 1.26 [0.75-2.11] |
| Fruit arboriculture | Both sexes | **1.17 [1.04-1.76]** | **1.35 [1.06-1.73]** | **1.12 [1.01-1.84]** | **1.15 [1.03-1.73]** | 0.96 [0.46-4.53] | **1.24 [1.16-1.97]** | **1.13 [1.03-1.94]** |
|  | Female | **1.49 [1.10-3.17]** | **1.85 [1.27-2.70]** | **1.57 [1.08-3.36]** | **1.44 [1.07-3.06]** | NC | **1.33 [1.09-3.01]** | **2.29 [1.04-5.19]** |
|  | Male | 1.00 [0.45-1.68] | 1.12 [0.81-1.55] | 0.90 [0.47-1.75] | 0.86 [0.45-1.67] | 0.78 [0.37-1.65] | NC | 1.03 [0.41-1.51] |
| Garden center/tree nursery | Both sexes | 1.16 [0.44-3.10] | 1.37 [0.81-2.32] | 1.03 [0.39-2.76] | 1.18 [0.44-3.17] | NC | 1.23 [0.46-3.28] | 1.36 [0.43-4.24] |
|  | Female | NC | NC | NC | NC | NC | NC | NC |
|  | Male | 1.20 [0.38-3.72] | 1.59 [0.90-2.81] | 1.04 [0.33-3.24] | 1.22 [0.39-3.80] | NC | 1.31 [0.42-4.09] | 1.79 [0.57-5.57] |
| Crop farming  (e.g., wheat, corn, and industrial grower) | Both sexes | **1.26 [1.06-1.49]** | **1.18 [1.04-1.33]** | **1.76 [1.48-2.09]** | **1.18 [1.00-1.40]** | 1.13 [0.73-1.77] | **1.23 [1.02-1.48]** | **1.19 [1.03-1.47]** |
|  | Female | 1.22 [0.89-1.68] | 1.00 [0.83-1.19] | **1.66 [1.19-2.30]** | 1.13 [0.82-1.56] | 1.11 [0.79-1.57] | 1.55 [0.90-4.00] | 1.08 [0.58-1.69] |
|  | Male | **1.31 [1.07-1.60]** | **1.27 [1.05-1.44]** | **1.85 [1.51-2.27]** | **1.24 [1.01-1.52]** | 1.05 [0.63-1.75] | **1.33 [1.08-1.66]** | **1.23 [1.04-1.57]** |
| Viticulture | Both sexes | **1.16 [1.00-1.45]** | **1.11 [1.01-1.27]** | **1.20 [1.04-1.52]** | **1.18 [1.01-1.48]** | 1.11 [0.77-2.22] | **1.13 [1.01-1.48]** | **1.24 [1.03-1.62]** |
|  | Female | 1.10 [0.73-1.65] | 0.95 [0.76-1.19] | 1.16 [0.76-1.75] | 1.09 [0.73-1.64] | 1.02 [0.31-3.43] | 1.11 [0.72-1.70] | 0.99 [0.58-1.69] |
|  | Male | **1.19 [1.04-1.58]** | **1.21 [1.05-1.40]** | **1.24 [1.06-1.64]** | **1.24 [1.03-1.63]** | 1.04 [0.78-2.51] | **1.26 [1.05-1.69]** | **1.36 [1.05-1.85]** |
| Sylviculture/forestry  (e.g., thinning, pruning) | Both sexes | NC | NC | NC | NC | NC | NC | NC |
|  | Female | NC | NC | NC | NC | NC | NC | NC |
|  | Male | NC | NC | NC | NC | NC | NC | NC |
| Unspecified specialized farming  (e.g., herbs, mushrooms) | Both sexes | 1.87 [0.93-3.76] | 1.25 [0.74-2.12] | 1.99 [0.99-3.99] | 1.78 [0.89-3.58] | NC | 1.85 [0.92-3.72] | 2.51 [0.94-5.56] |
|  | Female | NC | NC | NC | NC | NC | NC | NC |
|  | Male | **2.30 [1.03-5.15]** | 1.43 [0.97-4.66] | **2.35 [1.05-5.25]** | **2.23 [1.00-4.99]** | NC | **2.41 [1.08-5.40]** | **2.48 [1.11-6.72]** |
| Dairy farming | Both sexes | **1.22 [1.10-1.39]** | **1.28 [1.15-1.43]** | **1.33 [1.12-1.54]** | **1.16 [1.04-1.41]** | 0.94 [0.84-1.29] | **1.23 [1.13-1.43]** | **1.16 [1.04-1.51]** |
|  | Female | **1.34 [1.14-1.69]** | **1.46 [1.22-1.74]** | **1.39 [1.19-1.86]** | **1.70 [1.06-1.84]** | 1.09 [0.73-3.21] | **1.36 [1.04-1.75]** | **1.09 [1.01-1.83]** |
|  | Male | **1.14 [1.04-1.28]** | **1.16 [1.02-1.33]** | **1.09 [1.01-1.24]** | **1.18 [1.03-1.50]** | 0.87 [0.81-1.38] | **1.16 [1.09-1.42]** | **1.24 [1.05-1.75]** |
| Cow farming | Both sexes | 0.80 [0.61-1.05] | 0.98 [0.86-1.13] | 0.83 [0.56-1.07] | 0.82 [0.63-1.08] | 0.54 [0.25-1.17] | 0.73 [0.55-1.02] | 0.71 [0.51-1.00] |
|  | Female | 1.00 [0.63-1.58] | 1.11 [0.87-1.40] | 0.89 [0.56-1.42] | 1.05 [0.66-1.67] | NC | 1.07 [0.68-1.71] | 0.79 [0.41-1.50] |
|  | Male | 0.72 [0.52-1.01] | 0.93 [0.79-1.10] | 0.88 [0.69-1.05] | 0.74 [0.53-1.03] | 0.61 [0.42-1.18] | 0.78 [0.31-1.46] | 0.69 [0.46-1.03] |
| Mixed cattle farming | Both sexes | 0.94 [0.59-1.50] | **1.26 [1.02-1.57]** | 0.79 [0.49-1.26] | 1.00 [0.63-1.60] | 0.73 [0.42-1.27] | 1.25 [0.51-3.08] | 1.15 [0.70-1.89] |
|  | Female | 0.74 [0.28-2.00] | 1.10 [0.71-1.70] | 0.61 [0.23-1.65] | 0.83 [0.31-2.22] | NC | 0.61 [0.20-1.92] | 1.16 [0.43-3.13] |
|  | Male | 1.01 [0.59-1.72] | **1.30 [1.01-1.66]** | 0.86 [0.51-1.47] | 1.05 [0.62-1.79] | 0.77 [0.41-1.44] | 1.21 [0.74-3.29] | 1.14 [0.64-2.03] |
| Ovine and caprine farming | Both sexes | 0.95 [0.66-1.36] | 1.00 [0.82-1.23] | 0.96 [0.67-1.37] | 0.93 [0.65-1.33] | 0.84 [0.57-1.24] | 1.13 [0.46-2.76] | 1.05 [0.69-1.62] |
|  | Female | 1.48 [0.91-2.40] | 1.24 [0.92-1.68] | 1.43 [0.88-2.33] | 1.45 [0.89-2.35] | 1.27 [0.75-2.16] | 1.88 [0.85-9.69] | **2.18 [1.27-3.74]** |
|  | Male | 0.62 [0.36-1.08] | 0.86 [0.65-1.12] | 0.64 [0.37-1.11] | 0.61 [0.35-1.06] | NC | 0.57 [0.31-1.04] | 0.49 [0.23-1.04] |
| Pig farming | Both sexes | 1.02 [0.53-1.97] | 1.04 [0.73-1.48] | 0.86 [0.44-1.65] | 1.09 [0.56-2.10] | NC | 0.88 [0.42-1.85] | 1.29 [0.64-2.60] |
|  | Female | 1.52 [0.57-4.09] | 1.16 [0.62-2.16] | 1.28 [0.47-3.44] | 1.67 [0.62-4.49] | NC | 1.22 [0.39-3.81] | 2.47 [0.91-6.70] |
|  | Male | 0.80 [0.33-1.94] | 0.97 [0.63-1.49] | 0.68 [0.28-1.64] | 0.84 [0.35-2.03] | NC | 0.72 [0.27-1.94] | 0.87 [0.32-2.33] |
| Stud farming | Both sexes | 0.61 [0.29-1.28] | 0.63 [0.40-1.05] | 0.73 [0.35-1.54] | 0.55 [0.26-1.17] | NC | 0.55 [0.26-1.15] | 0.60 [0.23-1.62] |
|  | Female | 0.49 [0.16-1.53] | 0.54 [0.27-1.07] | 0.56 [0.18-1.75] | 0.45 [0.14-1.40] | NC | 0.45 [0.14-1.42] | NC |
|  | Male | 0.64 [0.24-1.70] | 0.68 [0.37-1.23] | 0.76 [0.28-2.04] | 0.59 [0.22-1.57] | NC | 0.61 [0.23-1.63] | NC |
| Unspecified large animal farming  (e.g., ostrich, llama) | Both sexes | NC | NC | NC | NC | NC | NC | NC |
|  | Female | NC | NC | NC | NC | NC | NC | NC |
|  | Male | NC | NC | NC | NC | NC | NC | NC |
| Poultry and rabbit farming | Both sexes | 0.90 [0.55-1.48] | 0.87 [0.45-1.46] | 0.81 [0.49-1.33] | 0.93 [0.57-1.53] | 0.77 [0.45-1.34] | 1.21 [0.38-3.82] | 0.88 [0.47-1.64] |
|  | Female | 0.92 [0.43-1.95] | 0.98 [0.41-2.02] | 0.84 [0.39-1.78] | 0.96 [0.45-2.03] | NC | 0.83 [0.37-1.88] | 0.88 [0.32-2.37] |
|  | Male | 0.86 [0.44-1.65] | 0.91 [0.63-1.32] | 0.76 [0.39-1.47] | 0.89 [0.46-1.71] | NC | 0.72 [0.34-1.53] | 0.85 [0.38-1.90] |
| Unspecified small animal farming  (e.g., frogs, snails, bees) | Both sexes | 0.69 [0.37-1.29] | **0.65 [0.43-0.97]** | 0.98 [0.52-1.84] | 0.62 [0.33-1.15] | NC | 0.59 [0.29-1.18] | 1.31 [0.70-2.45] |
|  | Female | 0.52 [0.19-1.41] | **0.41 [0.20-0.87]** | 0.82 [0.30-2.23] | 0.44 [0.16-1.20] | NC | 0.53 [0.20-1.42] | 1.11 [0.41-3.02] |
|  | Male | 0.75 [0.33-1.67] | 0.80 [0.49-1.30] | 0.96 [0.43-2.16] | 0.70 [0.31-1.57] | NC | 0.58 [0.22-1.55] | 1.29 [0.58-2.91] |
| Training, dressage, riding clubs | Both sexes | 0.78 [0.42-1.46] | 0.75 [0.49-1.14] | 0.75 [0.40-1.41] | 0.75 [0.40-1.41] | NC | 0.75 [0.40-1.41] | 0.70 [0.29-1.70] |
|  | Female | 0.89 [0.42-1.92] | 1.08 [0.65-1.79] | 0.86 [0.40-1.87] | 0.88 [0.41-1.88] | NC | 0.85 [0.39-1.83] | 0.71 [0.22-2.27] |
|  | Male | 0.45 [0.14-1.39] | 0.36 [0.16-1.11] | 0.40 [0.13-1.25] | 0.43 [0.14-1.34] | NC | 0.49 [0.16-1.51] | NC |
| Shellfish farming  (e.g., oyster farming, scallop aquaculture) | Both sexes | **2.51 [1.19-5.30]** | **1.75 [1.05-4.69]** | **2.42 [1.15-5.11]** | **2.43 [1.15-5.12]** | NC | **2.37 [1.06-5.29]** | **3.91 [1.75-8.77]** |
|  | Female | **5.48 [1.75-17.2]** | **5.13 [1.69-16.6]** | **5.18 [1.65-16.3]** | **5.30 [1.69-16.6]** | NC | NC | **10 [3.36-30]** |
|  | Male | 1.82 [0.68-4.89] | 1.73 [0.90-3.33] | 1.72 [0.64-4.61] | 1.77 [0.66-4.74] | NC | 2.06 [0.77-5.52] | 2.43 [0.78-7.58] |
| Unspecified and mixed farming  (e.g., polyculture, mixed farming, diversified farming) | Both sexes | **1.27 [1.02-1.58]** | **1.27 [1.13-1.43]** | **1.28 [1.01-1.47]** | **1.30 [1.04-1.61]** | 1.07 [0.84-2.45] | 1.47 [0.88-1.36] | **1.26 [1.00-1.64]** |
|  | Female | 1.16 [0.78-1.74] | 1.13 [0.91-1.41] | 1.07 [0.72-1.61] | 1.21 [0.81-1.81] | NC | 1.13 [0.74-1.73] | 0.84 [0.46-1.51] |
|  | Male | **1.33 [1.03-1.72]** | **1.32 [1.15-1.52]** | **1.25 [1.01-1.62]** | **1.34 [1.04-1.73]** | 1.04 [0.78-1.40] | 1.62 [0.93-2.81] | **1.43 [1.07-1.93]** |
| Salt works/salt evaporation pond | Both sexes | NC | NC | NC | NC | NC | NC | NC |
|  | Female | NC | NC | NC | NC | NC | NC | NC |
|  | Male | NC | NC | NC | NC | NC | NC | NC |
| Wood production  (e.g., lopping) | Both sexes | 0.43 [0.16-1.15] | 0.66 [0.40-1.10] | 0.40 [0.15-1.07] | 0.44 [0.17-1.19] | NC | 0.33 [0.11-1.03] | 0.55 [0.18-1.70] |
|  | Female | NC | NC | NC | NC | NC | NC | NC |
|  | Male | 0.47 [0.17-1.25] | 0.72 [0.43-1.20] | 0.42 [0.16-1.13] | 0.49 [0.18-1.30] | NC | 0.36 [0.11-1.11] | 0.59 [0.19-1.83] |
| Stationary sawmill  (e.g., edging, trimming, decking, debarking) | Both sexes | NC | NC | NC | NC | NC | NC | NC |
|  | Female | NC | NC | NC | NC | NC | NC | NC |
|  | Male | NC | NC | NC | NC | NC | NC | NC |
| Agricultural work companies  (e.g., pesticide applications, harvest reaping) | Both sexes | 0.79 [0.41-1.52] | 1.30 [0.93-1.81] | 0.74 [0.38-1.44] | 0.79 [0.41-1.54] | NC | 0.71 [0.35-1.42] | 1.06 [0.50-2.25] |
|  | Female | NC | NC | NC | NC | NC | NC | NC |
|  | Male | 0.84 [0.42-1.68] | 0.88 [0.38-1.73] | 0.77 [0.38-1.54] | 0.85 [0.42-1.71] | NC | 0.74 [0.35-1.56] | 1.24 [0.58-2.62] |
| Gardening, landscaping and reforestation companies | Both sexes | 0.98 [0.71-1.34] | 1.14 [0.94-1.39] | 0.92 [0.67-1.26] | 0.98 [0.72-1.35] | 0.92 [0.66-1.28] | 1.55 [0.62-3.85] | 1.20 [0.82-1.76] |
|  | Female | NC | NC | NC | NC | NC | NC | NC |
|  | Male | 1.13 [0.82-1.56] | 1.27 [0.94-1.55] | 1.03 [0.75-1.43] | 1.15 [0.83-1.58] | 1.63 [0.65-4.06] | 1.06 [0.75-1.49] | 1.38 [0.94-2.02] |
| Company representative/authorized representative | Both sexes | NC | NC | NC | NC | NC | NC | NC |
|  | Female | NC | NC | NC | NC | NC | NC | NC |
|  | Male | NC | NC | NC | NC | NC | NC | NC |
| Rural craftsperson  (e.g., mason, mechanics) | Both sexes | NC | NC | NC | NC | NC | NC | NC |
|  | Female | NC | NC | NC | NC | NC | NC | NC |
|  | Male | NC | NC | NC | NC | NC | NC | NC |

*Note*: HR: hazard ratio, MA: main analysis, NC: not calculated, SA: sensitivity analysis.

The main analysis (MA) was adjusted for sex (for both sexes only), age, first year of the farm’s establishment, farm location, number of pre-existing medical comorbidities, and the number of years performing the considered activity. SA1 was adjusted for sex (for both sexes only), age, first year of the farm’s establishment, farm location, pre-existing medical comorbidities (long-term illnesses), and the number of years performing the considered activity. SA2 was adjusted for sex (for both sexes only), age, first year of the farm’s establishment, farm location, and the number of years performing the considered activity. SA3 was adjusted for sex (for both sexes only), age, first year of the farm’s establishment, farm location, pre-existing mental health comorbidities (long-term illnesses for mental health issues such as depression and antidepressant drug reimbursement), and the number of years performing the considered activity. SA4 is an exposure-specific analysis for which the exposed group was restricted to all FMs who engaged in the specific activity for less than the median number of years of exposure of all exposed FMs (e.g., < 5 years). SA5 is an exposure-specific analysis for which the exposed group was restricted to all FMs who engaged in the specific activity for at least the median number of years of exposure of all exposed FMs (e.g., ≥ 5 years). Both SA4 and SA5 were adjusted for sex (for both sexes only), age, first year of the farm’s establishment, farm location, and number of pre-existing medical comorbidities. SA6 was restricted to cases identified from 2013-2016 and was adjusted for the same variables as the main analysis.

# **Table S5**: Farming activities and risk of ulcerative colitis - comparison of the main analysis with sensitivity analyses, TRACTOR project, France, 2002-2016

| **Agricultural activity** | **Sex** | **MA**  HR [95%CI] | **SA1**  HR [95%CI] | **SA2**  HR [95%CI] | **SA3**  HR [95%CI] | **SA4**  HR [95%CI] | **SA5**  HR [95%CI] | **SA6**  HR [95%CI] |
| --- | --- | --- | --- | --- | --- | --- | --- | --- |
| Truck farming, floriculture/flower-growing | Both sexes | 1.04 [0.76-1.42] | 1.09 [0.86-1.38] | 0.96 [0.70-1.32] | 1.04 [0.76-1.43] | 0.83 [0.34-2.01] | 0.98 [0.70-1.38] | 0.90 [0.59-1.36] |
|  | Female | 0.65 [0.31-1.38] | 0.89 [0.55-1.45] | 0.65 [0.30-1.37] | 0.64 [0.30-1.36] | NC | 0.49 [0.20-1.18] | 0.62 [0.23-1.67] |
|  | Male | 1.16 [0.82-1.65] | 1.16 [0.88-1.52] | 1.05 [0.74-1.49] | 1.17 [0.83-1.66] | 0.60 [0.19-1.88] | 1.17 [0.81-1.68] | 0.98 [0.62-1.55] |
| Fruit arboriculture | Both sexes | **1.45 [1.01-2.70]** | **1.37 [1.02-1.88]** | **1.42 [1.00-2.47]** | **1.41 [1.03-2.68]** | NC | **1.52 [1.03-1.83]** | **1.33 [1.00-2.99]** |
|  | Female | 1.36 [0.68-2.76] | **1.61 [1.01-2.57]** | 1.48 [0.73-3.00] | 1.32 [0.65-2.67] | NC | 1.49 [0.73-3.01] | 1.60 [0.71-3.62] |
|  | Male | 1.07 [0.67-1.71] | 1.13 [0.79-1.60] | 1.10 [0.69-1.76] | 1.07 [0.67-1.71] | NC | 1.13 [0.69-1.85] | 0.84 [0.45-1.58] |
| Garden center/tree nursery | Both sexes | 1.21 [0.54-2.69] | 1.54 [0.90-2.66] | 1.06 [0.47-2.36] | 1.23 [0.55-2.75] | NC | 1.32 [0.59-2.96] | 1.78 [0.80-3.97] |
|  | Female | NC | NC | NC | NC | NC | NC | NC |
|  | Male | 1.55 [0.69-3.45] | 1.43 [0.58-3.21] | 1.30 [0.58-2.91] | 1.58 [0.71-3.53] | NC | 1.75 [0.78-3.91] | 2.22 [0.99-4.96] |
| Crop farming  (e.g., wheat, corn, and industrial grower) | Both sexes | 0.90 [0.78-1.05] | 1.05 [0.94-1.17] | **1.31 [1.13-1.52]** | 1.05 [0.93-1.28] | 0.82 [0.70-1.07] | 1.24 [0.96-1.71] | 0.98 [0.83-1.17] |
|  | Female | 0.96 [0.71-1.29] | 1.14 [0.93-1.39] | 1.28 [0.94-1.74] | 0.88 [0.65-1.18] | 0.80 [0.40-2.08] | 0.91 [0.65-1.24] | 0.94 [0.65-1.36] |
|  | Male | 0.91 [0.77-1.08] | 1.04 [0.92-1.18] | **1.35 [1.13-1.60]** | 1.06 [0.72-1.22] | 0.83 [0.68-1.11] | 1.33 [0.94-1.88] | 1.02 [0.84-1.24] |
| Viticulture | Both sexes | **1.12 [1.01-1.28]** | **1.17 [1.03-1.34]** | **1.15 [1.02-1.39]** | **1.14 [1.03-1.37]** | 1.09 [0.82-1.81] | **1.22 [1.05-1.35]** | **1.25 [1.02-1.55]** |
|  | Female | 0.93 [0.63-1.35] | 1.02 [0.79-1.32] | 1.01 [0.68-1.49] | 0.92 [0.63-1.35] | 0.97 [0.38-2.48] | 0.91 [0.60-1.38] | 0.95 [0.60-1.50] |
|  | Male | **1.20 [1.04-1.49]** | **1.26 [1.07-1.47]** | **1.22 [1.05-1.51]** | **1.24 [1.01-1.54]** | 1.09 [0.83-2.00] | **1.18 [1.03-1.51]** | **1.38 [1.09-1.75]** |
| Sylviculture/forestry  (e.g., thinning, pruning) | Both sexes | NC | NC | NC | NC | NC | NC | NC |
|  | Female | NC | NC | NC | NC | NC | NC | NC |
|  | Male | NC | NC | NC | NC | NC | NC | NC |
| Unspecified specialized farming  (e.g., herbs, mushrooms) | Both sexes | 0.69 [0.26-1.83] | 1.06 [0.57-1.97] | 0.73 [0.27-1.94] | 0.66 [0.25-1.76] | NC | 0.52 [0.17-1.62] | 0.84 [0.27-2.60] |
|  | Female | NC | NC | NC | NC | NC | NC | NC |
|  | Male | NC | NC | NC | NC | NC | NC | NC |
| Dairy farming | Both sexes | **1.46 [1.25-1.70]** | **1.33 [1.18-1.50]** | **1.20 [1.02-1.41]** | **1.54 [1.32-1.79]** | 0.93 [0.64-1.36] | **1.41 [1.19-1.66]** | **1.31 [1.09-1.58]** |
|  | Female | **1.74 [1.32-2.29]** | **1.41 [1.14-1.74]** | **1.40 [1.04-1.87]** | **1.95 [1.48-2.57]** | 1.97 [0.98-3.96] | **1.65 [1.22-2.22]** | **1.72 [1.23-2.42]** |
|  | Male | **1.32 [1.09-1.58]** | **1.26 [1.10-1.46]** | **1.23 [1.03-1.46]** | **1.36 [1.13-1.64]** | 0.72 [0.46-1.14] | **1.28 [1.04-1.57]** | **1.16 [1.00-1.65]** |
| Cow farming | Both sexes | 0.93 [0.76-1.15] | 0.92 [0.79-1.08] | 0.86 [0.69-1.05] | 0.96 [0.78-1.18] | 0.84 [0.58-1.04] | 1.12 [0.74-1.70] | 0.94 [0.74-1.20] |
|  | Female | 0.94 [0.62-1.43] | 1.05 [0.79-1.40] | 0.83 [0.54-1.27] | 1.00 [0.66-1.52] | 1.34 [0.53-3.42] | 0.81 [0.51-1.30] | 0.84 [0.50-1.43] |
|  | Male | 0.93 [0.73-1.18] | 0.87 [0.73-1.05] | 0.88 [0.69-1.12] | 0.94 [0.74-1.20] | 0.71 [0.54-1.94] | 1.07 [0.67-1.71] | 0.97 [0.74-1.27] |
| Mixed cattle farming | Both sexes | 1.35 [0.98-1.86] | 1.06 [0.81-1.38] | 1.09 [0.79-1.51] | **1.43 [1.04-1.97]** | 0.67 [0.28-1.62] | 1.31 [0.93-1.85] | 1.22 [0.83-1.79] |
|  | Female | 1.03 [0.48-2.17] | 0.95 [0.55-1.64] | 0.80 [0.38-1.70] | 1.15 [0.54-2.44] | NC | 1.19 [0.56-2.52] | NC |
|  | Male | **1.43 [1.00-2.04]** | **1.86 [1.05-3.29]** | **1.52 [1.04-2.72]** | **1.49 [1.04-2.12]** | 0.80 [0.33-1.94] | 1.32 [0.89-1.94] | 1.43 [0.96-2.14] |
| Ovine and caprine farming | Both sexes | 0.96 [0.71-1.30] | 0.85 [0.67-1.09] | 0.98 [0.72-1.32] | 0.94 [0.70-1.28] | 0.72 [0.32-1.61] | 0.91 [0.65-1.25] | 1.05 [0.74-1.49] |
|  | Female | 1.11 [0.67-1.85] | 1.02 [0.69-1.50] | 1.07 [0.65-1.79] | 1.09 [0.66-1.81] | NC | 1.05 [0.61-1.81] | 1.55 [0.90-2.68] |
|  | Male | 0.88 [0.60-1.28] | 0.76 [0.56-1.04] | 0.91 [0.62-1.33] | 0.86 [0.59-1.26] | 0.62 [0.23-1.68] | 0.84 [0.56-1.26] | 0.84 [0.53-1.33] |
| Pig farming | Both sexes | 0.92 [0.52-1.63] | 0.98 [0.65-1.48] | 0.75 [0.43-1.33] | 0.98 [0.56-1.74] | NC | 0.89 [0.48-1.66] | 0.83 [0.41-1.66] |
|  | Female | 1.56 [0.64-3.77] | 1.29 [0.64-2.59] | 1.31 [0.54-3.17] | 1.73 [0.72-4.20] | NC | 1.74 [0.72-4.23] | 1.85 [0.69-4.98] |
|  | Male | 0.70 [0.34-1.48] | 0.85 [0.51-1.42] | 0.58 [0.27-1.21] | 0.74 [0.35-1.56] | NC | 0.59 [0.25-1.43] | 0.53 [0.20-1.41] |
| Stud farming | Both sexes | 0.58 [0.30-1.11] | **0.51 [0.30-0.88]** | 0.73 [0.38-1.42] | 0.52 [0.27-1.01] | NC | 0.52 [0.27-1.01] | 0.72 [0.34-1.51] |
|  | Female | 0.46 [0.15-1.43] | 0.45 [0.19-1.09] | 0.57 [0.18-1.78] | 0.44 [0.13-1.27] | NC | 0.43 [0.14-1.35] | NC |
|  | Male | 0.61 [0.27-1.36] | 0.52 [0.26-1.05] | 0.75 [0.34-1.68] | 0.56 [0.25-1.25] | NC | 0.57 [0.26-1.27] | 0.95 [0.43-2.14] |
| Unspecified large animal farming  (e.g., ostrich, llama) | Both sexes | NC | NC | NC | NC | NC | NC | NC |
|  | Female | NC | NC | NC | NC | NC | NC | NC |
|  | Male | NC | NC | NC | NC | NC | NC | NC |
| Poultry and rabbit farming | Both sexes | 1.25 [0.87-1.80] | 1.09 [0.82-1.47] | 1.09 [0.76-1.57] | 1.30 [0.90-1.87] | 1.11 [0.74-1.66] | 1.31 [0.88-2.96] | 1.21 [0.77-1.88] |
|  | Female | 1.50 [0.86-2.63] | 1.38 [0.90-2.12] | 1.37 [0.78-2.40] | 1.58 [0.90-2.77] | NC | 1.41 [0.77-2.59] | 1.80 [0.95-3.42] |
|  | Male | 1.07 [0.66-1.74] | 0.90 [0.60-1.34] | 0.91 [0.56-1.48] | 1.11 [0.69-1.80] | 0.93 [0.44-3.22] | 1.20 [0.54-1.61] | 0.89 [0.48-1.66] |
| Unspecified small animal farming  (e.g., frogs, snails, bees) | Both sexes | 0.75 [0.44-1.27] | **0.63 [0.40-0.99]** | 1.07 [0.63-1.82] | 0.67 [0.40-1.14] | NC | 0.72 [0.41-1.28] | 1.00 [0.55-1.81] |
|  | Female | 0.50 [0.19-1.34] | 0.47 [0.21-1.04] | 0.80 [0.29-2.17] | 0.41 [0.15-1.10] | NC | 0.52 [0.19-1.40] | 0.71 [0.23-2.23] |
|  | Male | 0.85 [0.45-1.58] | 0.71 [0.41-1.23] | 1.10 [0.59-2.07] | 0.80 [0.43-1.49] | NC | 0.83 [0.41-1.67] | 1.10 [0.55-2.22] |
| Training, dressage, riding clubs | Both sexes | 0.72 [0.40-1.31] | 0.75 [0.47-1.19] | 0.71 [0.39-1.29] | 0.70 [0.38-1.26] | 1.64 [0.52-5.12] | 0.54 [0.27-1.09] | 0.86 [0.43-1.73] |
|  | Female | 0.81 [0.36-1.84] | 0.90 [0.48-1.69] | 0.87 [0.38-1.99] | 0.78 [0.34-1.77] | NC | 0.66 [0.27-1.61] | 1.00 [0.37-2.72] |
|  | Male | 0.54 [0.22-1.29] | 0.55 [0.27-1.10] | 0.47 [0.19-1.13] | 0.52 [0.22-1.25] | NC | 0.37 [0.12-1.14] | 0.67 [0.25-1.78] |
| Shellfish farming  (e.g., oyster farming, scallop aquaculture) | Both sexes | 1.84 [0.87-3.87] | 1.51 [0.79-2.92] | 1.79 [0.85-3.77] | 1.78 [0.85-3.76] | NC | 1.82 [0.82-4.07] | **3.10 [1.47-6.53]** |
|  | Female | NC | NC | NC | NC | NC | NC | NC |
|  | Male | 1.93 [0.87-4.33] | 1.48 [0.70-3.10] | 1.81 [0.81-4.05] | 1.89 [0.84-4.22] | NC | 1.91 [0.79-4.60] | **3.23 [1.44-7.23]** |
| Unspecified and mixed farming  (e.g., polyculture, mixed farming, diversified farming) | Both sexes | 1.15 [0.96-1.38] | **1.16 [1.01-1.33]** | 1.06 [0.88-1.27] | 1.17 [0.97-1.40] | 0.97 [0.79-1.19] | 1.11 [0.93-1.68] | 1.10 [0.88-1.37] |
|  | Female | 0.89 [0.60-1.33] | 0.97 [0.74-1.28] | 0.82 [0.55-1.23] | 0.93 [0.62-1.38] | NC | 0.90 [0.59-1.36] | 0.89 [0.55-1.45] |
|  | Male | **1.24 [1.01-1.52]** | **1.22 [1.04-1.43]** | **1.26 [1.05-1.53]** | **1.25 [1.01-1.54]** | 0.99 [0.78-1.26] | 1.25 [0.81-1.94] | 1.16 [0.91-1.49] |
| Salt works/salt evaporation pond | Both sexes | NC | NC | NC | NC | NC | NC | NC |
|  | Female | NC | NC | NC | NC | NC | NC | NC |
|  | Male | NC | NC | NC | NC | NC | NC | NC |
| Wood production  (e.g., lopping) | Both sexes | 0.79 [0.42-1.47] | 0.76 [0.46-1.27] | 0.72 [0.38-1.34] | 0.82 [0.44-1.53] | NC | 0.67 [0.33-1.34] | 1.13 [0.58-2.18] |
|  | Female | NC | NC | NC | NC | NC | NC | NC |
|  | Male | 0.85 [0.46-1.59] | 0.82 [0.49-1.36] | 0.74 [0.39-1.38] | 0.89 [0.47-1.66] | NC | 0.71 [0.36-1.44] | 1.20 [0.62-2.32] |
| Stationary sawmill  (e.g., edging, trimming, decking, debarking) | Both sexes | NC | NC | NC | NC | NC | NC | NC |
|  | Female | NC | NC | NC | NC | NC | NC | NC |
|  | Male | NC | NC | NC | NC | NC | NC | NC |
| Agricultural work companies  (e.g., pesticide applications, harvest reaping) | Both sexes | 1.38 [0.90-2.13] | **1.48 [1.05-2.07]** | 1.30 [0.84-2.02] | 1.40 [0.91-2.17] | 1.10 [0.67-1.80] | 1.41 [0.99-5.86] | **1.80 [1.11-2.92]** |
|  | Female | NC | NC | NC | NC | NC | NC | NC |
|  | Male | 1.44 [0.91-2.27] | **1.55 [1.09-2.21]** | 1.30 [0.82-2.05] | 1.47 [0.93-2.32] | 1.10 [0.65-1.87] | **2.55 [1.05-6.20]** | **2.03 [1.25-3.30]** |
| Gardening, landscaping and reforestation companies | Both sexes | 0.88 [0.66-1.18] | 1.09 [0.88-1.35] | 0.82 [0.61-1.10] | 0.89 [0.67-1.19] | 1.05 [0.49-2.25] | 0.85 [0.62-1.16] | 1.11 [0.80-1.55] |
|  | Female | 1.58 [0.59-4.27] | 1.29 [0.54-3.13] | 1.66 [0.61-4.49] | 1.53 [0.57-4.12] | NC | 1.56 [0.58-4.21] | NC |
|  | Male | 0.92 [0.68-1.24] | 1.15 [0.93-1.44] | 0.80 [0.59-1.09] | 0.93 [0.69-1.25] | 0.87 [0.51-2.33] | 1.09 [0.63-1.20] | 1.17 [0.83-1.64] |
| Company representative/authorized representative | Both sexes | 2.30 [0.74-7.15] | 2.29 [0.76-6.93] | 2.80 [0.90-8.73] | 2.25 [0.72-7.00] | NC | 2.16 [0.69-6.72] | NC |
|  | Female | NC | NC | NC | NC | NC | NC | NC |
|  | Male | NC | NC | NC | NC | NC | NC | NC |
| Rural craftsperson  (e.g., mason, mechanics) | Both sexes | NC | NC | NC | NC | NC | NC | NC |
|  | Female | NC | NC | NC | NC | NC | NC | NC |
|  | Male | NC | NC | NC | NC | NC | NC | NC |

*Note*: HR: hazard ratio, MA: main analysis, NC: not calculated, SA: sensitivity analysis.

The main analysis (MA) was adjusted for sex (for both sexes only), age, first year of the farm’s establishment, farm location, number of pre-existing medical comorbidities, and the number of years performing the considered activity. SA1 was adjusted for sex (for both sexes only), age, first year of the farm’s establishment, farm location, pre-existing medical comorbidities (long-term illnesses), and the number of years performing the considered activity. SA2 was adjusted for sex (for both sexes only), age, first year of the farm’s establishment, farm location, and the number of years performing the considered activity. SA3 was adjusted for sex (for both sexes only), age, first year of the farm’s establishment, farm location, pre-existing mental health comorbidities (long-term illnesses for mental health issues such as depression and antidepressant drug reimbursement), and the number of years performing the considered activity. SA4 is an exposure-specific analysis for which the exposed group was restricted to all FMs who engaged in the specific activity for less than the median number of years of exposure of all exposed FMs (e.g., < 5 years). SA5 is an exposure-specific analysis for which the exposed group was restricted to all FMs who engaged in the specific activity for at least the median number of years of exposure of all exposed FMs (e.g., ≥ 5 years). Both SA4 and SA5 were adjusted for sex (for both sexes only), age, first year of the farm’s establishment, farm location, and number of pre-existing medical comorbidities. SA6 was restricted to cases identified from 2013-2016 and was adjusted for the same variables as the main analysis.

# **Table S6**: Farming activities and risk of inflammatory bowel disease - comparison of the main analysis with sensitivity analyses, TRACTOR project, France, 2002-2016

| **Agricultural activity** | **Sex** | **MA**  HR [95%CI] | **SA1**  HR [95%CI] | **SA2**  HR [95%CI] | **SA3**  HR [95%CI] | **SA4**  HR [95%CI] | **SA5**  HR [95%CI] | **SA6**  HR [95%CI] |
| --- | --- | --- | --- | --- | --- | --- | --- | --- |
| Truck farming, floriculture/flower-growing | Both sexes | 1.04 [0.81-1.32] | 0.96 [0.78-1.18] | 0.97 [0.76-1.23] | 1.03 [0.81-1.32] | 0.93 [0.58-1.51] | 0.97 [0.75-1.26] | 1.02 [0.75-1.38] |
|  | Female | 0.75 [0.45-1.26] | 0.78 [0.52-1.17] | 0.74 [0.44-1.24] | 0.74 [0.44-1.23] | NC | 0.67 [0.39-1.17] | 0.79 [0.41-1.53] |
|  | Male | 1.13 [0.86-1.49] | 1.03 [0.81-1.31] | 1.04 [0.79-1.36] | 1.14 [0.86-1.50] | 1.01 [0.60-1.68] | 1.09 [0.81-1.46] | 1.08 [0.77-1.53] |
| Fruit arboriculture | Both sexes | **1.52 [1.13-2.52]** | **1.25 [1.09-1.68]** | **1.69 [1.17-2.44]** | **1.11 [1.02-2.30]** | 0.84 [0.45-1.57] | **1.43 [1.11-2.55]** | **1.43 [1.03-3.04]** |
|  | Female | 1.42 [0.85-2.37] | 1.17 [0.86-1.58] | 1.52 [0.91-2.55] | 1.37 [0.82-2.30] | NC | 1.42 [0.83-2.41] | **1.88 [1.06-3.36]** |
|  | Male | 1.00 [0.68-1.46] | 1.05 [0.77-1.42] | 1.03 [0.70-1.50] | 0.99 [0.68-1.45] | 0.93 [0.48-1.79] | 0.99 [0.66-1.50] | 0.74 [0.44-1.25] |
| Garden center/tree nursery | Both sexes | 1.19 [0.64-2.21] | 1.37 [0.85-2.20] | 1.04 [0.56-1.95] | 1.21 [0.65-2.26] | 1.18 [0.38-3.67] | 1.28 [0.69-2.39] | 1.61 [0.84-3.11] |
|  | Female | NC | NC | NC | NC | NC | NC | NC |
|  | Male | 1.41 [0.73-2.71] | 1.63 [0.98-2.70] | 1.20 [0.62-2.31] | 1.44 [0.75-2.77] | 1.37 [0.44-4.28] | 1.57 [0.82-3.03] | 2.05 [0.99-3.96] |
| Crop farming  (e.g., wheat, corn, and industrial grower) | Both sexes | 1.03 [0.93-1.16] | 0.98 [0.90-1.08] | **1.48 [1.32-1.66]** | 0.97 [0.87-1.09] | 0.98 [0.86-1.11] | **1.26 [1.05-1.51]** | 1.06 [0.93-1.21] |
|  | Female | 1.07 [0.86-1.33] | 0.98 [0.83-1.16] | **1.44 [1.15-1.80]** | 0.99 [0.79-1.23] | 0.99 [0.63-1.61] | 1.01 [0.79-1.25] | 1.02 [0.78-1.35] |
|  | Male | 1.05 [0.92-1.20] | 1.01 [0.91-1.12] | **1.53 [1.34-1.75]** | 1.00 [0.87-1.13] | 1.01 [0.87-1.17] | **1.33 [1.09-1.62]** | 1.10 [0.94-1.28] |
| Viticulture | Both sexes | 1.13 [0.98-1.30] | 1.08 [0.97-1.22] | **1.17 [1.01-1.35]** | **1.16 [1.00-1.34]** | 1.04 [0.90-1.44] | 1.11 [0.94-1.30] | **1.25 [1.06-1.47]** |
|  | Female | 1.00 [0.76-1.32] | 0.92 [0.74-1.14] | 1.07 [0.81-1.42] | 1.00 [0.76-1.32] | 1.00 [0.74-2.06] | 1.27 [0.78-1.45] | 0.97 [0.68-1.37] |
|  | Male | **1.20 [1.02-1.42]** | **1.18 [1.03-1.35]** | **1.23 [1.03-1.45]** | **1.24 [1.05-1.47]** | 1.11 [0.85-1.45] | **1.18 [1.03-1.42]** | **1.37 [1.14-1.65]** |
| Sylviculture/forestry  (e.g., thinning, pruning) | Both sexes | NC | NC | NC | NC | NC | NC | NC |
|  | Female | NC | NC | NC | NC | NC | NC | NC |
|  | Male | NC | NC | NC | NC | NC | NC | NC |
| Unspecified specialized farming  (e.g., herbs, mushrooms) | Both sexes | 1.19 [0.67-2.10] | 1.12 [0.69-1.83] | 1.26 [0.71-2.22] | 1.14 [0.64-2.01] | NC | 1.09 [0.60-1.98] | 1.5 [0.78-2.89] |
|  | Female | 1.02 [0.38-2.72] | 0.89 [0.37-2.15] | 1.17 [0.44-3.13] | 0.93 [0.35-2.49] | NC | 0.98 [0.37-2.63] | 1.94 [0.72-5.20] |
|  | Male | 1.21 [0.61-2.43] | 1.22 [0.67-2.20] | 1.22 [0.61-2.45] | 1.18 [0.59-2.36] | NC | 1.12 [0.53-2.35] | 1.20 [0.50-2.90] |
| Dairy farming | Both sexes | **1.30 [1.15-1.47]** | **1.33 [1.21-1.47]** | **1.31 [1.18-1.45]** | **1.37 [1.22-1.55]** | 1.10 [0.89-1.35] | **1.24 [1.09-1.42]** | **1.19 [1.03-1.39]** |
|  | Female | **1.37 [1.10-1.70]** | **1.45 [1.23-1.71]** | **1.33 [1.10-1.62]** | **1.52 [1.22-1.90]** | **1.30 [1.02-2.40]** | **1.58 [1.03-1.65]** | **1.33 [1.04-1.75]** |
|  | Male | **1.25 [1.08-1.44]** | **1.24 [1.10-1.40]** | **1.30 [1.15-1.47]** | **1.29 [1.11-1.49]** | 0.99 [0.77-1.25] | **1.29 [1.09-1.40]** | **1.14 [1.01-1.34]** |
| Cow farming | Both sexes | 0.88 [0.74-1.03] | 0.97 [0.85-1.10] | 0.91 [0.68-1.05] | 0.90 [0.77-1.07] | 0.83 [0.61-1.15] | 0.88 [0.68-1.05] | 0.85 [0.70-1.03] |
|  | Female | 0.96 [0.71-1.31] | 1.10 [0.88-1.37] | 0.86 [0.63-1.17] | 1.02 [0.75-1.39] | 0.93 [0.56-1.84] | 1.01 [0.67-1.29] | 0.82 [0.54-1.23] |
|  | Male | 0.85 [0.70-1.03] | 0.91 [0.79-1.07] | 0.80 [0.66-1.07] | 0.86 [0.71-1.05] | 0.77 [0.54-1.16] | 0.86 [0.64-1.04] | 0.86 [0.69-1.08] |
| Mixed cattle farming | Both sexes | 1.19 [0.91-1.55] | **1.28 [1.05-1.57]** | 0.97 [0.75-1.27] | 1.26 [0.97-1.64] | 0.90 [0.58-1.41] | 1.07 [0.80-1.44] | 1.19 [0.88-1.61] |
|  | Female | 0.90 [0.49-1.63] | 1.16 [0.78-1.73] | 0.72 [0.39-1.31] | 1.00 [0.55-1.83] | NC | 0.92 [0.49-1.73] | 0.71 [0.32-1.60] |
|  | Male | 1.27 [0.94-1.70] | **1.30 [1.03-1.65]** | 1.07 [0.80-1.44] | 1.32 [0.98-1.77] | 0.98 [0.61-1.56] | 1.10 [0.79-1.53] | 1.32 [0.95-1.84] |
| Ovine and caprine farming | Both sexes | 0.96 [0.76-1.21] | 1.01 [0.84-1.21] | 0.97 [0.77-1.22] | 0.94 [0.75-1.18] | **0.59 [0.36-0.99]** | 0.88 [0.68-1.13] | 1.05 [0.80-1.38] |
|  | Female | 1.28 [0.90-1.82] | 1.23 [0.93-1.63] | 1.24 [0.87-1.76] | 1.26 [0.89-1.78] | 0.98 [0.43-2.23] | 1.15 [0.79-1.68] | **1.82 [1.24-2.68]** |
|  | Male | 0.78 [0.57-1.06] | 0.88 [0.69-1.12] | 0.80 [0.59-1.09] | 0.76 [0.56-1.04] | **0.47 [0.24-0.90]** | 0.73 [0.52-1.02] | 0.71 [0.48-1.04] |
| Pig farming | Both sexes | 0.96 [0.63-1.48] | 1.02 [0.73-1.43] | 0.79 [0.52-1.22] | 1.02 [0.67-1.57] | 0.74 [0.35-1.55] | 0.88 [0.55-1.42] | 1.01 [0.62-1.65] |
|  | Female | 1.53 [0.79-2.97] | 1.21 [0.69-2.14] | 1.29 [0.67-2.50] | 1.70 [0.88-3.28] | NC | 1.49 [0.74-3.01] | **2.10 [1.04-4.25]** |
|  | Male | 0.74 [0.42-1.31] | 0.93 [0.61-1.40] | 0.61 [0.35-1.08] | 0.78 [0.44-1.37] | 0.63 [0.26-1.53] | 0.64 [0.33-1.24] | 0.65 [0.33-1.31] |
| Stud farming | Both sexes | **0.59 [0.36-0.97]** | **0.62 [0.41-0.93]** | 0.74 [0.45-1.20] | **0.54 [0.33-0.88]** | NC | **0.54 [0.33-0.88]** | 0.67 [0.37-1.22] |
|  | Female | 0.47 [0.21-1.06] | 0.55 [0.29-1.02] | 0.56 [0.25-1.26] | **0.43 [0.19-0.96]** | NC | **0.44 [0.20-0.99]** | 0.42 [0.13-1.30] |
|  | Male | 0.62 [0.33-1.15] | 0.64 [0.38-1.08] | 0.75 [0.40-1.40] | 0.57 [0.31-1.06] | NC | 0.58 [0.31-1.09] | 0.79 [0.39-1.58] |
| Unspecified large animal farming  (e.g., ostrich, llama) | Both sexes | NC | NC | NC | NC | NC | NC | NC |
|  | Female | NC | NC | NC | NC | NC | NC | NC |
|  | Male | NC | NC | NC | NC | NC | NC | NC |
| Poultry and rabbit farming | Both sexes | 1.10 [0.82-1.47] | 1.06 [0.83-1.35] | 0.97 [0.72-1.30] | 1.14 [0.85-1.53] | 0.96 [0.69-1.92] | 1.17 [0.71-1.33] | 1.07 [0.75-1.54] |
|  | Female | 1.23 [0.78-1.92] | 1.28 [0.91-1.82] | 1.12 [0.72-1.76] | 1.29 [0.82-2.01] | 1.13 [0.51-3.06] | 1.25 [0.70-1.84] | 1.38 [0.81-2.36] |
|  | Male | 0.99 [0.67-1.45] | 0.89 [0.63-1.25] | 0.85 [0.58-1.26] | 1.02 [0.69-1.51] | 0.84 [0.54-2.01] | 1.11 [0.61-1.31] | 0.87 [0.53-1.43] |
| Unspecified small animal farming  (e.g., frogs, snails, bees) | Both sexes | 0.72 [0.48-1.08] | **0.67 [0.47-0.96]** | 1.03 [0.69-1.55] | **0.65 [0.43-0.97]** | 1.35 [0.67-2.71] | 0.66 [0.43-1.03] | 1.12 [0.73-1.73] |
|  | Female | 0.51 [0.25-1.03] | **0.47 [0.25-0.88]** | 0.80 [0.39-1.62] | **0.43 [0.21-0.86]** | NC | 0.52 [0.26-1.05] | 0.90 [0.42-1.90] |
|  | Male | 0.80 [0.49-1.32] | 0.79 [0.51-1.21] | 1.04 [0.64-1.71] | 0.75 [0.46-1.24] | 1.61 [0.76-3.40] | 0.72 [0.41-1.28] | 1.17 [0.69-1.99] |
| Training, dressage, riding clubs | Both sexes | 0.76 [0.49-1.16] | 0.77 [0.53-1.11] | 0.74 [0.48-1.13] | 0.73 [0.47-1.12] | 0.54 [0.17-1.67] | 0.65 [0.41-1.03] | 0.80 [0.46-1.38] |
|  | Female | 0.86 [0.49-1.50] | 1.02 [0.64-1.62] | 0.87 [0.49-1.54] | 0.83 [0.48-1.46] | NC | 0.76 [0.43-1.36] | 0.86 [0.40-1.85] |
|  | Male | **0.50 [0.25-1.00]** | **0.47 [0.25-0.87]** | **0.44 [0.22-0.88]** | **0.48 [0.24-0.97]** | NC | **0.42 [0.19-0.93]** | 0.60 [0.27-1.33] |
| Shellfish farming  (e.g., oyster farming, scallop aquaculture) | Both sexes | **2.12 [1.25-3.59]** | **1.69 [1.05-2.80]** | **2.06 [1.21-3.49]** | **2.06 [1.21-3.48]** | 2.06 [0.93-7.05] | **2.06 [1.16-3.64]** | **3.43 [1.98-5.93]** |
|  | Female | **3.46 [1.29-9.26]** | **2.35 [1.08-6.29]** | **3.32 [1.24-8.89]** | **3.34 [1.25-8.95]** | NC | **2.72 [1.08-8.49]** | **6.28 [2.34-16.9]** |
|  | Male | **1.89 [1.01-3.52]** | **1.58 [1.00-2.86]** | **1.77 [1.01-3.30]** | **1.84 [1.00-3.43]** | NC | **1.97 [1.02-3.80]** | **2.91 [1.51-5.62]** |
| Unspecified and mixed farming  (e.g., polyculture, mixed farming, diversified farming) | Both sexes | **1.20 [1.04-1.38]** | **1.23 [1.10-1.38]** | **1.21 [1.06-1.37]** | **1.22 [1.06-1.40]** | 1.01 [0.86-1.18] | 1.15 [0.91-1.46] | **1.16 [1.00-1.37]** |
|  | Female | 1.01 [0.76-1.34] | 1.11 [0.90-1.37] | 0.93 [0.70-1.24] | 1.05 [0.79-1.39] | 1.00 [0.55-1.81] | 1.00 [0.75-1.35] | 0.87 [0.59-1.26] |
|  | Male | **1.27 [1.08-1.50]** | **1.28 [1.12-1.46]** | **1.20 [1.02-1.41]** | **1.28 [1.09-1.51]** | 1.01 [0.84-1.22] | 1.19 [0.92-1.55] | **1.26 [1.04-1.53]** |
| Salt works/salt evaporation pond | Both sexes | NC | NC | NC | NC | NC | NC | NC |
|  | Female | NC | NC | NC | NC | NC | NC | NC |
|  | Male | NC | NC | NC | NC | NC | NC | NC |
| Wood production  (e.g., lopping) | Both sexes | 0.63 [0.37-1.07] | 0.69 [0.44-1.07] | **0.58 [0.34-0.99]** | 0.66 [0.39-1.12] | 0.97 [0.40-2.33] | **0.52 [0.29-0.95]** | 0.89 [0.50-1.57] |
|  | Female | NC | NC | NC | NC | NC | NC | NC |
|  | Male | 0.69 [0.41-1.17] | 0.74 [0.48-1.15] | 0.61 [0.36-1.03] | 0.72 [0.42-1.22] | 0.99 [0.41-2.39] | 0.56 [0.31-1.02] | 0.95 [0.54-1.68] |
| Stationary sawmill  (e.g., edging, trimming, decking, debarking) | Both sexes | NC | NC | NC | NC | NC | NC | NC |
|  | Female | NC | NC | NC | NC | NC | NC | NC |
|  | Male | NC | NC | NC | NC | NC | NC | NC |
| Agricultural work companies  (e.g., pesticide applications, harvest reaping) | Both sexes | 1.13 [0.78-1.62] | 1.24 [0.92-1.67] | 1.06 [0.74-1.53] | 1.14 [0.80-1.64] | 0.93 [0.63-2.54] | 1.26 [0.82-1.39] | **1.50 [1.00-2.25]** |
|  | Female | 0.92 [0.30-2.87] | 0.92 [0.35-2.47] | 0.96 [0.31-3.01] | 0.91 [0.29-2.84] | NC | 0.91 [0.29-2.84] | NC |
|  | Male | 1.19 [0.81-1.74] | 1.32 [0.96-1.80] | 1.08 [0.73-1.58] | 1.21 [0.83-1.77] | 0.95 [0.61-2.70] | 1.34 [0.67-1.46] | **1.71 [1.14-2.57]** |
| Gardening, landscaping and reforestation companies | Both sexes | 0.92 [0.75-1.14] | 1.03 [0.86-1.23] | 0.86 [0.70-1.07] | 0.93 [0.75-1.15] | 1.22 [0.80-1.84] | 0.88 [0.70-1.10] | 1.15 [0.90-1.48] |
|  | Female | 0.79 [0.29-2.11] | 0.77 [0.32-1.85] | 0.79 [0.29-2.11] | 0.76 [0.28-2.04] | NC | 0.77 [0.29-2.06] | NC |
|  | Male | 1.01 [0.81-1.25] | 1.13 [0.94-1.35] | 0.90 [0.72-1.12] | 1.02 [0.82-1.27] | 1.27 [0.84-1.93] | 0.95 [0.75-1.20] | 1.25 [0.97-1.62] |
| Company representative/authorized representative | Both sexes | 1.24 [0.40-3.84] | 1.33 [0.56-4.26] | 1.52 [0.49-4.73] | 1.21 [0.39-3.75] | NC | 1.14 [0.37-3.53] | NC |
|  | Female | NC | NC | NC | NC | NC | NC | NC |
|  | Male | NC | NC | NC | NC | NC | NC | NC |
| Rural craftsperson  (e.g., mason, mechanics) | Both sexes | NC | NC | NC | NC | NC | NC | NC |
|  | Female | NC | NC | NC | NC | NC | NC | NC |
|  | Male | NC | NC | NC | NC | NC | NC | NC |

*Note*: HR: hazard ratio, MA: main analysis, NC: not calculated, SA: sensitivity analysis.

The main analysis (MA) was adjusted for sex (for both sexes only), age, first year of the farm’s establishment, farm location, number of pre-existing medical comorbidities, and the number of years performing the considered activity. SA1 was adjusted for sex (for both sexes only), age, first year of the farm’s establishment, farm location, pre-existing medical comorbidities (long-term illnesses), and the number of years performing the considered activity. SA2 was adjusted for sex (for both sexes only), age, first year of the farm’s establishment, farm location, and the number of years performing the considered activity. SA3 was adjusted for sex (for both sexes only), age, first year of the farm’s establishment, farm location, pre-existing mental health comorbidities (long-term illnesses for mental health issues such as depression and antidepressant drug reimbursement), and the number of years performing the considered activity. SA4 is an exposure-specific analysis for which the exposed group was restricted to all FMs who engaged in the specific activity for less than the median number of years of exposure of all exposed FMs (e.g., < 5 years). SA5 is an exposure-specific analysis for which the exposed group was restricted to all FMs who engaged in the specific activity for at least the median number of years of exposure of all exposed FMs (e.g., ≥ 5 years). Both SA4 and SA5 were adjusted for sex (for both sexes only), age, first year of the farm’s establishment, farm location, and number of pre-existing medical comorbidities. SA6 was restricted to cases identified from 2013-2016 and was adjusted for the same variables as the main analysis.

# **Table S7**: Hazard ratio for a one-year exposure increase by agricultural activity and IBD

| **Agricultural activity** | **Sex** | **CD**: HR [95%CI] | **UC**: HR [95%CI] | **IBD**: HR [95%CI] |
| --- | --- | --- | --- | --- |
| Truck farming, floriculture/flower-growing | Both sexes | 1.01 [0.97-1.05] | 1.00 [0.97-1.04] | 1.00 [0.98-1.03] |
|  | Female | 1.01 [0.88-1.23] | 1.01 [0.94-1.13] | 0.97 [0.87-1.14] |
|  | Male | 0.99 [0.94-1.03] | 1.00 [0.97-1.04] | 0.99 [0.97-1.03] |
| Fruit arboriculture | Both sexes | **1.006 [1.001-1.053]** | **1.004 [1.001-1.042]** | **1.004 [1.001-1.041]** |
|  | Female | 1.012 [0.946-1.042] | 0.998 [0.927-1.058] | 1.011 [0.954-1.067] |
|  | Male | 0.994 [0.935-1.045] | 0.992 [0.943-1.042] | 0.993 [0.952-1.038] |
| Garden center/tree nursery | Both sexes | 1.02 [0.88-1.27] | 0.97 [0.89-1.06] | 1.02 [0.95-1.09] |
|  | Female | not calculated | not calculated | not calculated |
|  | Male | 1.01 [0.78-1.35] | 0.98 [0.90-1.08] | 1.03 [0.96-1.11] |
| Crop farming (e.g., wheat, corn, and industrial grower) | Both sexes | **1.007 [1.002-1.072]** | 1.003 [0.998-1.021] | **1.006 [1.002-1.069]** |
|  | Female | 1.000 [0.981-1.022] | 0.994 [0.983-1.014] | 1.004 [0.992-1.021] |
|  | Male | **1.012 [1.002-1.087]** | 1.001 [0.991-1.016] | **1.013 [1.003-1.074]** |
| Viticulture | Both sexes | 1.003 [0.984-1.038] | **1.005 [1.002-1.028]** | 1.004 [0.988-1.041] |
|  | Female | 1.000 [0.954-1.040] | 0.994 [0.972-1.010] | 0.993 [0.974-1.012] |
|  | Male | **1.008 [1.001-1.057]** | **1.006 [1.001-1.041]** | **1.003 [1.000-1.051]** |
| Sylviculture/forestry (e.g., thinning, pruning) | Both sexes | not calculated | not calculated | not calculated |
|  | Female | not calculated | not calculated | not calculated |
|  | Male | not calculated | not calculated | not calculated |
| Unspecified specialized farming (e.g., herbs, mushrooms) | Both sexes | 0.99 [0.92-1.06] | 0.98 [0.89-1.08] | 0.99 [0.94-1.04] |
|  | Female | not calculated | not calculated | 0.98 [0.86-1.07] |
|  | Male | 1.01 [0.94-1.10] | not calculated | 1.00 [0.94-1.07] |
| Dairy farming | Both sexes | **1.009 [1.003-1.045]** | **1.005 [1.001-1.032]** | **1.009 [1.002-1.043]** |
|  | Female | **1.003 [1.001-1.072]** | **1.004 [1.001-1.072]** | **1.005 [1.001-1.057]** |
|  | Male | 1.004 [0.982-1.031] | **1.008 [1.001-1.042]** | 1.004 [0.995-1.026] |
| Cow farming | Both sexes | 1.01 [0.96-1.04] | 0.98 [0.88-1.14] | 0.99 [0.84-1.02] |
|  | Female | 1.01 [0.97-1.08] | 0.99 [0.96-1.02] | 1.01 [0.98-1.05] |
|  | Male | 0.98 [0.89-1.06] | 1.01 [0.96-1.06] | 0.99 [0.87-1.07] |
| Mixed cattle farming | Both sexes | 0.98 [0.92-1.03] | 0.98 [0.94-1.02] | 0.99 [0.95-1.04] |
|  | Female | 0.99 [0.87-1.13] | 0.98 [0.93-1.03] | 0.98 [0.94-1.03] |
|  | Male | 0.98 [0.91-1.04] | 1.01 [0.93-1.13] | 1.01 [0.94-1.09] |
| Ovine and caprine farming | Both sexes | 0.99 [0.86-1.09] | 0.98 [0.95-1.02] | 1.01 [0.98-1.03] |
|  | Female | 1.00 [0.92-1.06] | 1.00 [0.96-1.06] | 1.01 [0.95-1.08] |
|  | Male | 0.98 [0.86-1.02] | 0.97 [0.93-1.01] | 0.99 [0.96-1.02] |
| Pig farming | Both sexes | 0.99 [0.88-1.05] | 1.01 [0.96-1.09] | 1.01 [0.93-1.15] |
|  | Female | 1.02 [0.90-1.17] | 1.02 [0.92-1.17] | 1.02 [0.95-1.13] |
|  | Male | 0.99 [0.82-1.11] | 0.99 [0.90-1.09] | 0.99 [0.98-1.01] |
| Stud farming | Both sexes | 0.991 [0.982-1.045] | 0.998 [0.985-1.036] | **0.991 [0.984-0.999]** |
|  | Female | 0.993 [0.961-1.013] | 0.987 [0.975-1.010] | **0.989 [0.981-0.995]** |
|  | Male | 0.992 [0.981-1.036] | 0.999 [0.951-1.048] | 1.001 [0.959-1.042] |
| Unspecified large animal farming (e.g., ostrich, llama) | Both sexes | not calculated | not calculated | not calculated |
|  | Female | not calculated | not calculated | not calculated |
|  | Male | not calculated | not calculated | not calculated |
| Poultry and rabbit farming | Both sexes | 0.98 [0.87-1.04] | 1.02 [0.98-1.06] | 0.99 [0.96-1.03] |
|  | Female | 1.01 [0.92-1.10] | 1.02 [0.95-1.09] | 1.02 [0.96-1.07] |
|  | Male | 0.97 [0.89-1.11] | 1.02 [0.97-1.07] | 0.97 [0.92-1.01] |
| Unspecified small animal farming (e.g., frogs, snails, bees) | Both sexes | 0.99 [0.92-1.06] | 0.97 [0.89-1.11] | 0.98 [0.92-1.01] |
|  | Female | 1.01 [0.88-1.17] | 0.99 [0.86-1.13] | 1.00 [0.91-1.10] |
|  | Male | 0.99 [0.91-1.08] | 0.98 [0.87-1.10] | 0.97 [0.91-1.07] |
| Training, dressage, riding clubs | Both sexes | 0.978 [0.899-1.019] | 1.002 [0.981-1.016] | 1.001 [0.938-1.035] |
|  | Female | 0.984 [0.838-1.013] | 1.001 [0.946-1.025] | 1.002 [0.955-1.037] |
|  | Male | 0.989 [0.849-1.022] | 1.004 [0.939-1.015] | 0.992 [0.988-1.007] |
| Shellfish farming (e.g., oyster farming, scallop aquaculture) | Both sexes | 1.006 [0.996-1.042] | 1.005 [0.954-1.061] | **1.004 [1.002-1.099]** |
|  | Female | **1.007 [1.001-1.081]** | not calculated | **1.003 [1.001-1.123]** |
|  | Male | 0.976 [0.863-1.092] | 1.001 [0.917-1.102] | **1.005 [1.001-1.073]** |
| Unspecified and mixed farming (e.g., polyculture, mixed farming, diversified farming) | Both sexes | 1.00 [0.97-1.08] | 1.01 [0.97-1.04] | 1.01 [0.97-1.02] |
|  | Female | 1.01 [0.96-1.10] | 1.01 [0.94-1.08] | 1.01 [0.98-1.04] |
|  | Male | 1.00 [0.98-1.05] | 1.01 [0.96-1.05] | 1.00 [0.97-1.03] |
| Salt works/salt evaporation pond | Both sexes | not calculated | not calculated | not calculated |
|  | Female | not calculated | not calculated | not calculated |
|  | Male | not calculated | not calculated | not calculated |
| Wood production (e.g., lopping) | Both sexes | 0.98 [0.84-1.06] | 0.98 [0.78-1.04] | 0.96 [0.88-1.05] |
|  | Female | not calculated | not calculated | not calculated |
|  | Male | 0.99 [0.84-1.09] | 0.99 [0.87-1.07] | 0.98 [0.88-1.06] |
| Stationary sawmill (e.g., edging, trimming, decking, debarking) | Both sexes | not calculated | not calculated | not calculated |
|  | Female | not calculated | not calculated | not calculated |
|  | Male | not calculated | not calculated | not calculated |
| Agricultural work companies (e.g., pesticide applications, harvest reaping) | Both sexes | 1.02 [0.95-1.10] | 1.01 [0.92-1.05] | 1.01 [0.94-1.07] |
|  | Female | not calculated | not calculated | 0.99 [0.93-1.11] |
|  | Male | 1.01 [0.96-1.11] | 1.00 [0.90-1.13] | 1.01 [0.95-1.10] |
| Gardening, landscaping and reforestation companies | Both sexes | 1.01 [0.96-1.05] | 1.01 [0.97-1.05] | 1.01 [0.97-1.04] |
|  | Female | not calculated | 0.98 [0.78-1.02] | 0.97 [0.88-1.13] |
|  | Male | 1.02 [0.95-1.06] | 1.02 [0.96-1.06] | 1.02 [0.97-1.05] |
| Company representative/authorized representative | Both sexes | not calculated | 0.98 [0.80-1.08] | 0.99 [0.82-1.07] |
|  | Female | not calculated | not calculated | not calculated |
|  | Male | not calculated | not calculated | not calculated |
| Rural craftsperson (e.g., mason, mechanics) | Both sexes | not calculated | not calculated | not calculated |
|  | Female | not calculated | not calculated | not calculated |
|  | Male | not calculated | not calculated | not calculated |

*Note*: CD: Crohn’s disease, HR: hazard ratio, IBD: inflammatory bowel disease, UC: ulcerative colitis.

# **STROBE Statement Checklist**

|  | Item  No | Recommendation | Page |
| --- | --- | --- | --- |
| **Title and abstract** | 1 | (*a*) Indicate the study’s design with a commonly used term in the title or the abstract | 1, 3 |
|  |  | (*b*) Provide in the abstract an informative and balanced summary of what was done and what was found | 3 |
| Introduction | | |  |
| Background/rationale | 2 | Explain the scientific background and rationale for the investigation being reported | 4, 5 |
| Objectives | 3 | State specific objectives, including any prespecified hypotheses | 5 |
| Methods | | |  |
| Study design | 4 | Present key elements of study design early in the paper | 5-7 |
| Setting | 5 | Describe the setting, locations, and relevant dates, including periods of recruitment, exposure, follow-up, and data collection | 5-7 |
| Participants | 6 | (*a*) Give the eligibility criteria, and the sources and methods of selection of participants. Describe methods of follow-up | 5-7 |
|  |  | (*b*) For matched studies, give matching criteria and number of exposed and unexposed | DNA |
| Variables | 7 | Clearly define all outcomes, exposures, predictors, potential confounders, and effect modifiers. Give diagnostic criteria, if applicable | 7-9, Fig. S1 |
| Data sources/ measurement | 8* | For each variable of interest, give sources of data and details of methods of assessment (measurement). Describe comparability of assessment methods if there is more than one group | 7-9 |
| Bias | 9 | Describe any efforts to address potential sources of bias | 7-9 |
| Study size | 10 | Explain how the study size was arrived at | 5-9 |
| Quantitative variables | 11 | Explain how quantitative variables were handled in the analyses. If applicable, describe which groupings were chosen and why | 7-9 |
| Statistical methods | 12 | (*a*) Describe all statistical methods, including those used to control for confounding | 7-9 |
|  |  | (*b*) Describe any methods used to examine subgroups and interactions | 7-9 |
|  |  | (*c*) Explain how missing data were addressed | 5-7 |
|  |  | (*d*) If applicable, explain how loss to follow-up was addressed | 8 |
|  |  | (*e*) Describe any sensitivity analyses | 8, 9 |
| Results | | |  |
| Participants | 13* | (a) Report numbers of individuals at each stage of study-e.g., numbers potentially eligible, examined for eligibility, confirmed eligible, included in the study, and analyzed | 9, 10 |
|  |  | (b) Give reasons for non-participation at each stage | DNA |
|  |  | (c) Consider use of a flow diagram | DNA |
| Descriptive data | 14* | (a) Give characteristics of study participants (e.g., demographic, clinical, social) and information on exposures and potential confounders | 9, 10, Table S1 |
|  |  | (b) Indicate number of participants with missing data for each variable of interest | DNA |
|  |  | (c) Summarize follow-up time (e.g., average and total amount) | 10 |
| Outcome data | 15* | Report numbers of outcome events or summary measures over time | 10, Tables 1, 2, S2, S3 |
| Main results | 16 | (*a*) Give unadjusted estimates and, if applicable, confounder-adjusted estimates and their precision (e.g., 95% confidence interval). Make clear which confounders were adjusted for and why they were included | 11-15, Tables 1, 2, Suppl |
|  |  | (*b*) Report category boundaries when continuous variables were categorized | DNA |
|  |  | (*c*) If relevant, consider translating estimates of relative risk into absolute risk for a meaningful time period | DNA |
| Other analyses | 17 | Report other analyses done-e.g., analyses of subgroups and interactions, and sensitivity analyses | 11-15, Suppl |
| Discussion | | |  |
| Key results | 18 | Summarize key results with reference to study objectives | 15 |
| Limitations | 19 | Discuss limitations of the study, taking into account sources of potential bias or imprecision. Discuss both direction and magnitude of any potential bias | 16, 23-26 |
| Interpretation | 20 | Give a cautious overall interpretation of results considering objectives, limitations, multiplicity of analyses, results from similar studies, and other relevant evidence | 16-26 |
| Generalizability | 21 | Discuss the generalizability (external validity) of the study results | 26 |
| Other information | | |  |
| Funding | 22 | Give the source of funding and the role of the funders for the present study and, if applicable, for the original study on which the present article is based | 29 |

*Give information separately for exposed and unexposed groups.

DNA: does not applied, Suppl: supplementary materials.


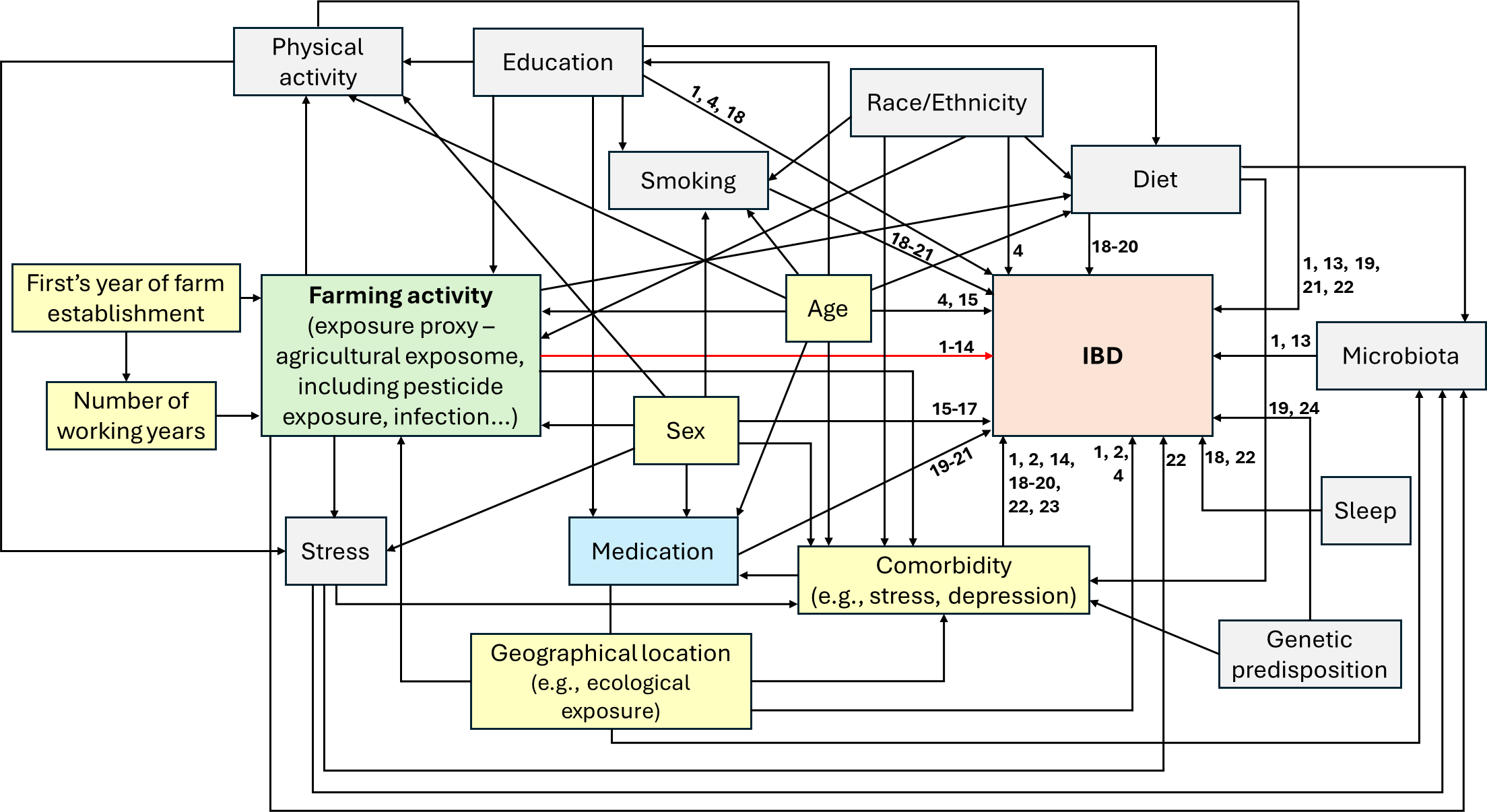


# **Figure S1**: Directed acyclic graph for variable selection

The directed acyclic graph (DAG) is based on published literature and expert knowledge. It consists of nodes (rectangles) and arrows representing variables and the causal associations between them, respectively. This DAG was used to guide covariate selection for assessing the association between farming activity and inflammatory bowel disease (IBD) among the entire French farm manager population. The green node labeled “Farming activity” indicates the exposure, and the orange one with “IBD” indicates the outcome. The red line pointing from the exposure to the outcome depicts the association of interest. Yellow nodes denote the minimally sufficient adjustment set, which takes into account all important confounders needed for obtaining unbiased estimates; blue nodes denote an ancestor (i.e., predictor) of the outcome that is not part of the minimally sufficient adjustment set; and gray nodes denote variables that were unmeasured or unavailable for the entire study population. The main associations (i.e., possible or established risk/protective factors) derived from the literature are marked by a number in the DAG and listed below:

1. Ananthakrishnan AN, Bernstein CN, Iliopoulos D, et al. Environmental triggers in IBD: a review of progress and evidence. *Nat Rev Gastroenterol Hepatol* 2018;15(1):39–49.
2. Carbonnel F, Jantchou P, Monnet E, et al. Environmental risk factors in Crohn's disease and ulcerative colitis: an update. *Gastroenterol Clin Biol* 2009;33Suppl3:S145–157.
3. Chen X, Wang S, Mao X, et al. Adverse health effects of emerging contaminants on inflammatory bowel disease. *Front Public Health* 2023;11:1140786.
4. Chen D, Parks CG, Hofmann JN, Beane Freeman LE, Sandler DP. Pesticide use and inflammatory bowel disease in licensed pesticide applicators and spouses in the Agricultural Health Study. *Environ Res* 2024. doi:10.1016/j.envres.2024.118464.
5. Dow CT, Sechi LA. Cows Get Crohn's Disease and They're Giving Us Diabetes. *Microorganisms* 2019;7(10):466.
6. Eslami M, Shafiei M, Ghasemian A, et al. Mycobacterium avium paratuberculosis and Mycobacterium avium complex and related subspecies as causative agents of zoonotic and occupational diseases. *J Cell Physiol* 2019;234(8):12415–12421.
7. Gois MFB, Fernández-Pato A, Huss A, et al. Impact of occupational pesticide exposure on the human gut microbiome. *Front Microbiol* 2023;14:1223120.
8. Golomazou E, Mamedova S, Eslahi AV, Karanis P. Cryptosporidium and agriculture: A review. *Sci Total Environ* 2024;916:170057.
9. Khan MF, Wang H. Environmental Exposures and Autoimmune Diseases: Contribution of Gut Microbiome. *Front Immunol* 2020;10:3094.
10. Lerebours A, Bathie M, Kazour M, Amara R, Huet V, Thomas H. Spatio-temporal contamination of microplastics in shellfish farming regions: A case study. *Mar Pollut Bull* 2022;181:113842.
11. Leso V, Ricciardi W, Iavicoli I. Occupational risk factors in inflammatory bowel disease*. Eur Rev Med Pharmacol Sci* 2015;19(15):2838–2851.
12. McDaniel CJ, Cardwell DM, et al. Humans and cattle: a review of bovine zoonoses. *Vector Borne Zoonotic Dis* 2014; 14(1):1–19.
13. Vieujean S, Caron B, Haghnejad V, et al. Impact of the Exposome on the Epigenome in Inflammatory Bowel Disease Patients and Animal Models. *Int J Mol Sci* 2022;23(14):7611.
14. Ye Y, Pang Z, Chen W, Ju S, Zhou C. The epidemiology and risk factors of inflammatory bowel disease. *Int J Clin Exp Med* 2015;8(12):22529-22542.
15. Greuter T, Manser C, Pittet V, Vavricka SR, Biedermann L; on behalf of Swiss IBDnet, an official working group of the Swiss Society of Gastroenterology. Gender Differences in Inflammatory Bowel Disease. *Digestion* 2020;101 Suppl 1:98-104.
16. Shah SC, Khalili H, Gower-Rousseau C, et al. Sex-Based Differences in Incidence of Inflammatory Bowel Diseases-Pooled Analysis of Population-Based Studies From Western Countries. *Gastroenterology* 2018;155(4):1079-1089.e3.
17. Xu L, Huang G, Cong Y, Yu Y, Li Y. Sex-related Differences in Inflammatory Bowel Diseases: The Potential Role of Sex Hormones. *Inflamm Bowel Dis* 2022;28(11):1766-1775.
18. van der Sloot KWJ, Weersma RK, Alizadeh BZ, Dijkstra G. Identification of Environmental Risk Factors Associated With the Development of Inflammatory Bowel Disease. *J Crohns Colitis* 2020;14(12):1662-1671.
19. Gajendran M, Loganathan P, Catinella AP, et al. A comprehensive review and update on Crohn's disease. *Dis Mon* 2018;64(2):20–57.
20. Maaser C, Langholz E, Gordon H, et al. European Crohn's and Colitis Organisation Topical Review on Environmental Factors in IBD. *J Crohns Colitis* 2017;11(8):905–920.
21. Piovani D, Danese S, Peyrin-Biroulet L, et al. Environmental Risk Factors for Inflammatory Bowel Diseases: An Umbrella Review of Meta-analyses. *Gastroenterology* 2019;157(3):647-659.e4.
22. Ananthakrishnan AN. Epidemiology and risk factors for IBD. *Nat Rev Gastroenterol Hepatol* 2015;12(4):205–217.
23. Bernstein CN, Nugent Z, Shaffer S, Singh H, Marrie RA. Comorbidity before and after a diagnosis of inflammatory bowel disease. *Aliment Pharmacol Ther* 2021;54(5):637-651.
24. Guan Q. A Comprehensive Review and Update on the Pathogenesis of Inflammatory Bowel Disease. *J Immunol Res* 2019;2019:7247238.


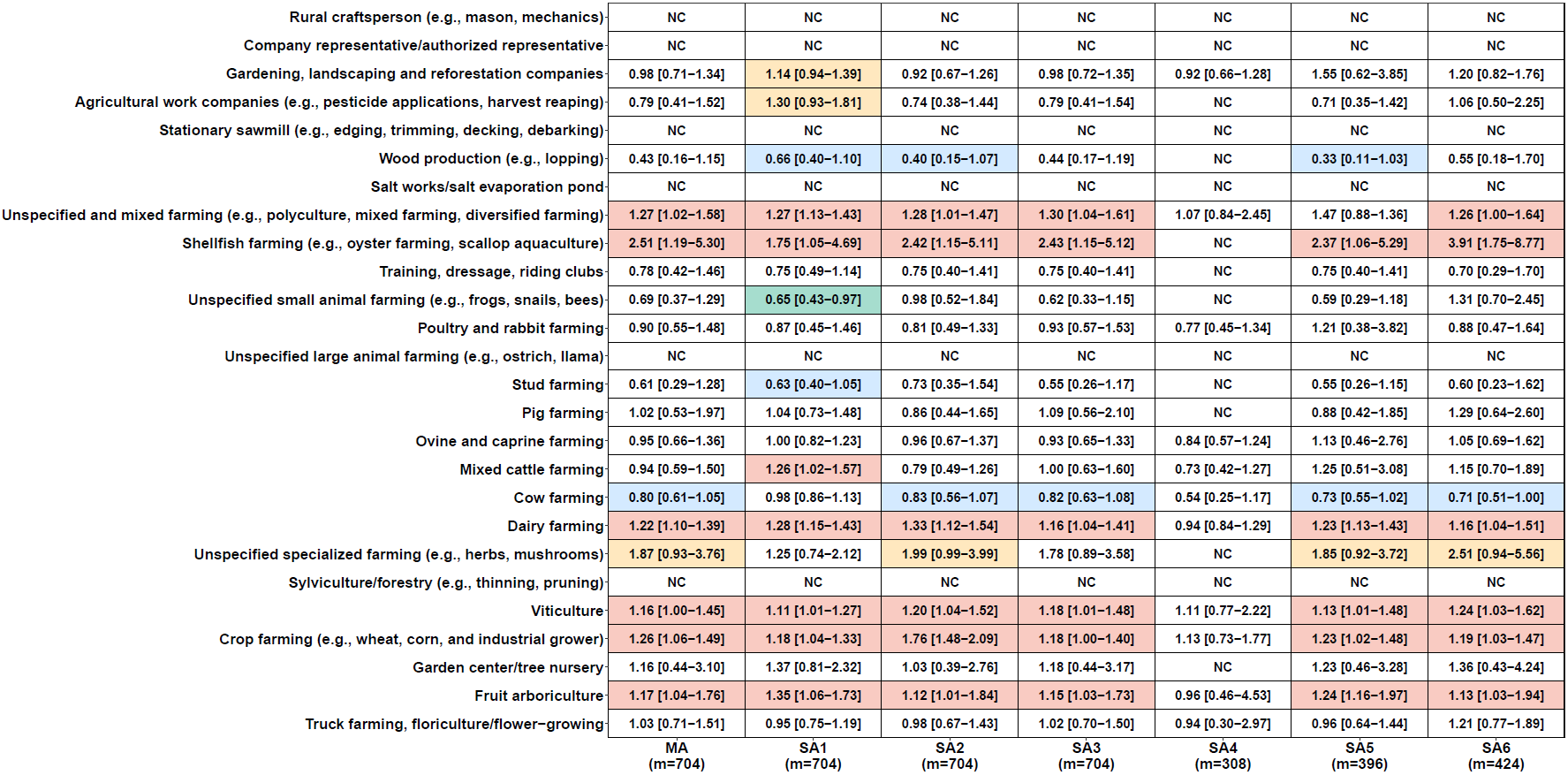


# **Figure S2**: Farming activities and risk of Crohn’s disease among all farm managers - comparison of the main analysis with sensitivity

m: number of exposed cases, NC: not calculated (<3 exposed cases), SA: sensitivity analysis. The white cells represent instances where there is no difference in the risk of CD between FMs engaged in the specified activity and those who are not, while the red and green cells indicate an elevated and reduced risk of CD, respectively. Orange and blue cells refer to positive and negative trends, respectively. The main analysis (MA) was adjusted for sex, age, first year of the farm’s establishment, farm location, number of pre-existing medical comorbidities, and the number of years performing the considered activity. SA1 was adjusted for sex, age, first year of the farm’s establishment, farm location, pre-existing medical comorbidities (long-term illnesses), and the number of years performing the considered activity. SA2 was adjusted for sex, age, first year of the farm’s establishment, farm location, and the number of years performing the considered activity. SA3 was adjusted for sex, age, first year of the farm’s establishment, farm location, pre-existing mental health comorbidities (long-term illnesses for mental health issues such as depression and antidepressant drug reimbursement), and the number of years performing the considered activity. SA4 is an exposure-specific analysis for which the exposed group was restricted to all FMs who engaged in the specific activity for less than the median number of years of exposure of all exposed FMs (e.g., < 5 years). SA5 is an exposure-specific analysis for which the exposed group was restricted to all FMs who engaged in the specific activity for at least the median number of years of exposure of all exposed FMs (e.g., ≥ 5 years). Both SA4 and SA5 were adjusted for sex, age, first year of the farm’s establishment, farm location, and number of pre-existing medical comorbidities. SA6 was restricted to cases identified from 2013-2016 and was adjusted for the same variables as the main analysis.


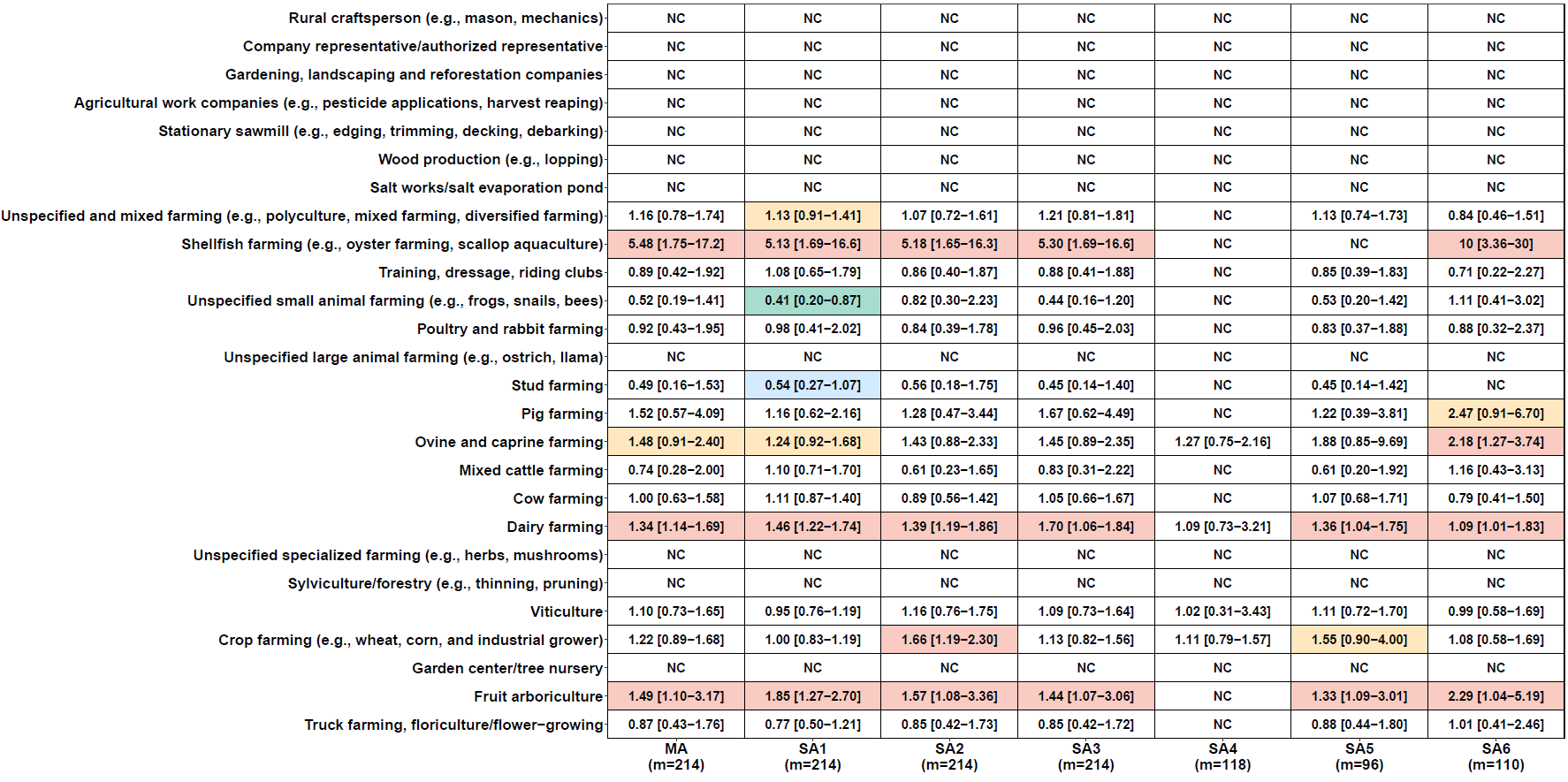


# **Figure S3**: Farming activities and risk of Crohn’s disease among female farm managers - comparison of the main analysis with sensitivity analyses

m: number of exposed cases, NC: not calculated (<3 exposed cases), SA: sensitivity analysis. The white cells represent instances where there is no difference in the risk of CD between FMs engaged in the specified activity and those who are not, while the red and green cells indicate an elevated and reduced risk of CD, respectively. Orange and blue cells refer to positive and negative trends, respectively. The main analysis was adjusted for age, first year of the farm’s establishment, farm location, number of pre-existing medical comorbidities, and the number of years performing the considered activity. SA1 was adjusted for age, first year of the farm’s establishment, farm location, pre-existing medical comorbidities (long-term illnesses), and the number of years performing the considered activity. SA2 was adjusted for age, first year of the farm’s establishment, farm location, and the number of years performing the considered activity. SA3 was adjusted for age, first year of the farm’s establishment, farm location, pre-existing mental health comorbidities (long-term illnesses for mental health issues such as depression and antidepressant drug reimbursement), and the number of years performing the considered activity. SA4 is an exposure-specific analysis for which the exposed group was restricted to all FMs who engaged in the specific activity for less than the median number of years of exposure of all exposed FMs (e.g., < 5 years). SA5 is an exposure-specific analysis for which the exposed group was restricted to all FMs who engaged in the specific activity for at least the median number of years of exposure of all exposed FMs (e.g., ≥ 5 years). Both SA4 and SA5 were adjusted for age, first year of the farm’s establishment, farm location, and number of pre-existing medical comorbidities. SA6 was restricted to cases identified from 2013-2016 and was adjusted for the same variables as the main analysis.


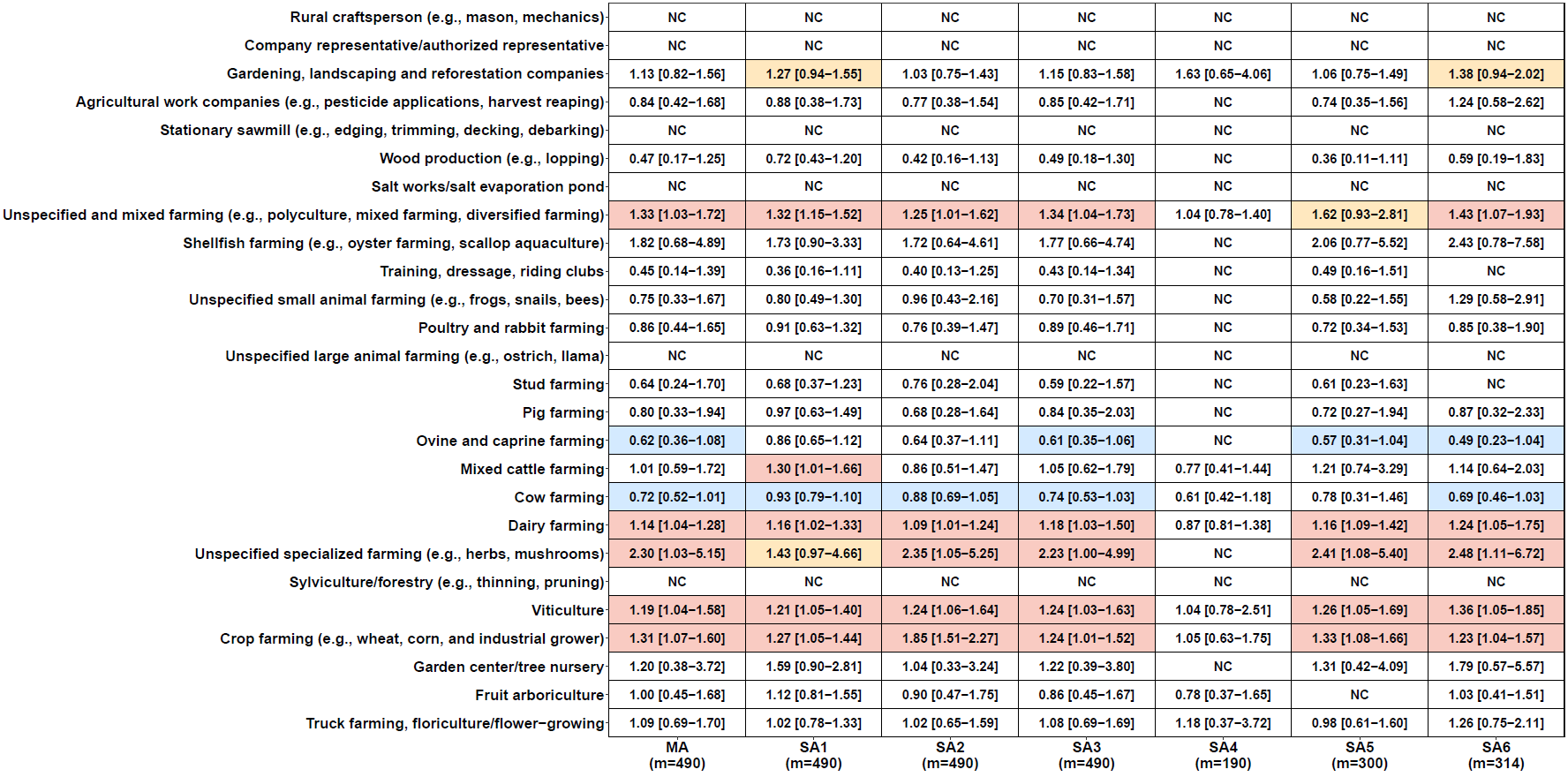


# **Figure S4**: Farming activities and risk of Crohn’s disease among male farm managers - comparison of the main analysis with sensitivity analyses

m: number of exposed cases, NC: not calculated (<3 exposed cases), SA: sensitivity analysis. The white cells represent instances where there is no difference in the risk of CD between FMs engaged in the specified activity and those who are not, while the red and green cells indicate an elevated and reduced risk of CD, respectively. Orange and blue cells refer to positive and negative trends, respectively. The main analysis was adjusted for age, first year of the farm’s establishment, farm location, number of pre-existing medical comorbidities, and the number of years performing the considered activity. SA1 was adjusted for age, first year of the farm’s establishment, farm location, pre-existing medical comorbidities (long-term illnesses), and the number of years performing the considered activity. SA2 was adjusted for age, first year of the farm’s establishment, farm location, and the number of years performing the considered activity. SA3 was adjusted for age, first year of the farm’s establishment, farm location, pre-existing mental health comorbidities (long-term illnesses for mental health issues such as depression and antidepressant drug reimbursement), and the number of years performing the considered activity. SA4 is an exposure-specific analysis for which the exposed group was restricted to all FMs who engaged in the specific activity for less than the median number of years of exposure of all exposed FMs (e.g., < 5 years). SA5 is an exposure-specific analysis for which the exposed group was restricted to all FMs who engaged in the specific activity for at least the median number of years of exposure of all exposed FMs (e.g., ≥ 5 years). Both SA4 and SA5 were adjusted for age, first year of the farm’s establishment, farm location, and number of pre-existing medical comorbidities. SA6 was restricted to cases identified from 2013-2016 and was adjusted for the same variables as the main analysis.


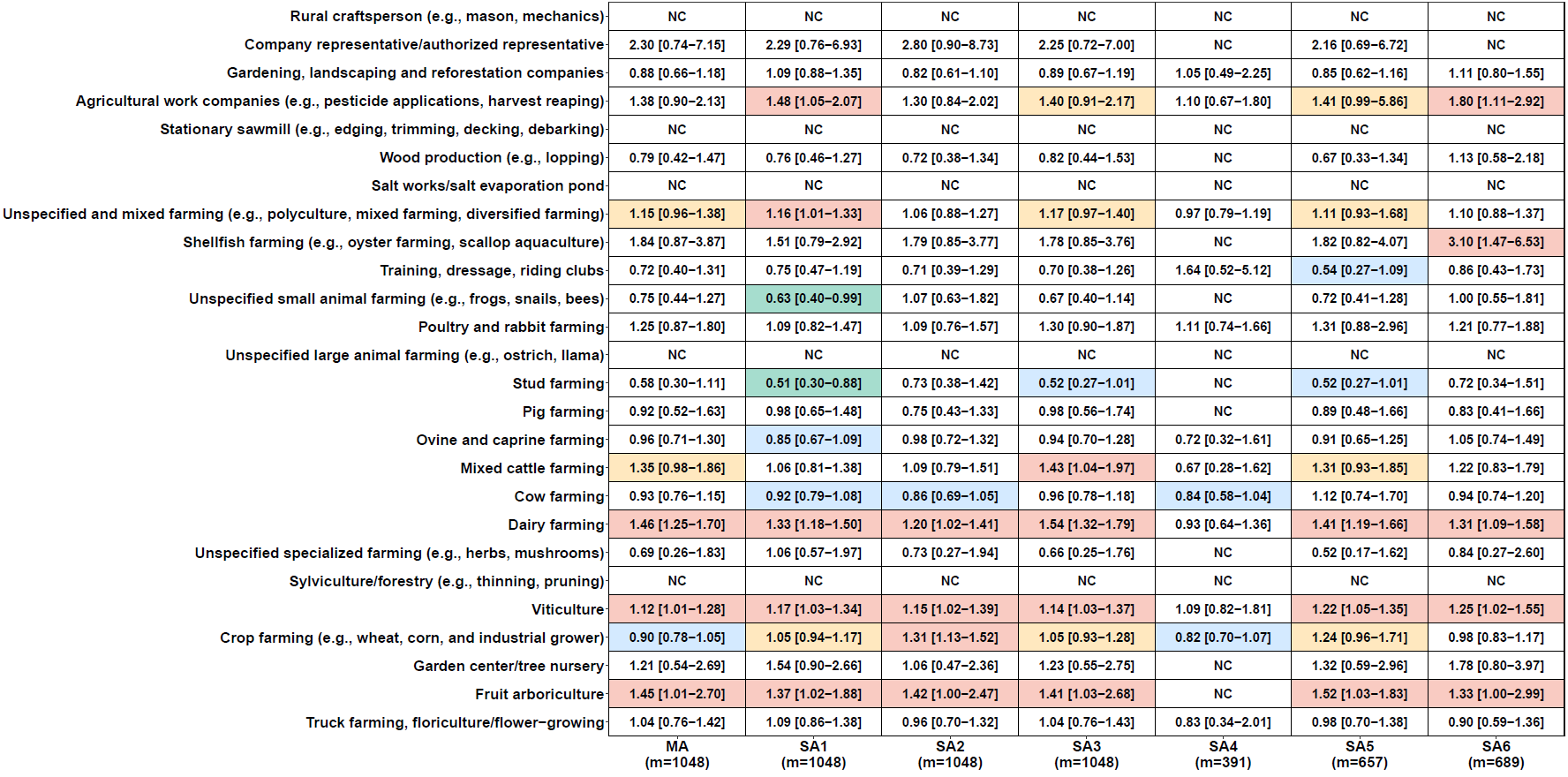


# **Figure S5**: Farming activities and risk of ulcerative colitis among all farm managers - comparison of the main analysis with sensitivity

m: number of exposed cases, NC: not calculated (<3 exposed cases), SA: sensitivity analysis. The white cells represent instances where there is no difference in the risk of UC between FMs engaged in the specified activity and those who are not, while the red and green cells indicate an elevated and reduced risk of UC, respectively. Orange and blue cells refer to positive and negative trends, respectively. The main analysis was adjusted for sex, age, first year of the farm’s establishment, farm location, number of pre-existing medical comorbidities, and the number of years performing the considered activity. SA1 was adjusted for sex, age, first year of the farm’s establishment, farm location, pre-existing medical comorbidities (long-term illnesses), and the number of years performing the considered activity. SA2 was adjusted for sex, age, first year of the farm’s establishment, farm location, and the number of years performing the considered activity. SA3 was adjusted for sex, age, first year of the farm’s establishment, farm location, pre-existing mental health comorbidities (long-term illnesses for mental health issues such as depression and antidepressant drug reimbursement), and the number of years performing the considered activity. SA4 is an exposure-specific analysis for which the exposed group was restricted to all FMs who engaged in the specific activity for less than the median number of years of exposure of all exposed FMs (e.g., < 5 years). SA5 is an exposure-specific analysis for which the exposed group was restricted to all FMs who engaged in the specific activity for at least the median number of years of exposure of all exposed FMs (e.g., ≥ 5 years). Both SA4 and SA5 were adjusted for sex, age, first year of the farm’s establishment, farm location, and number of pre-existing medical comorbidities. SA6 was restricted to cases identified from 2013-2016 and was adjusted for the same variables as the main analysis.


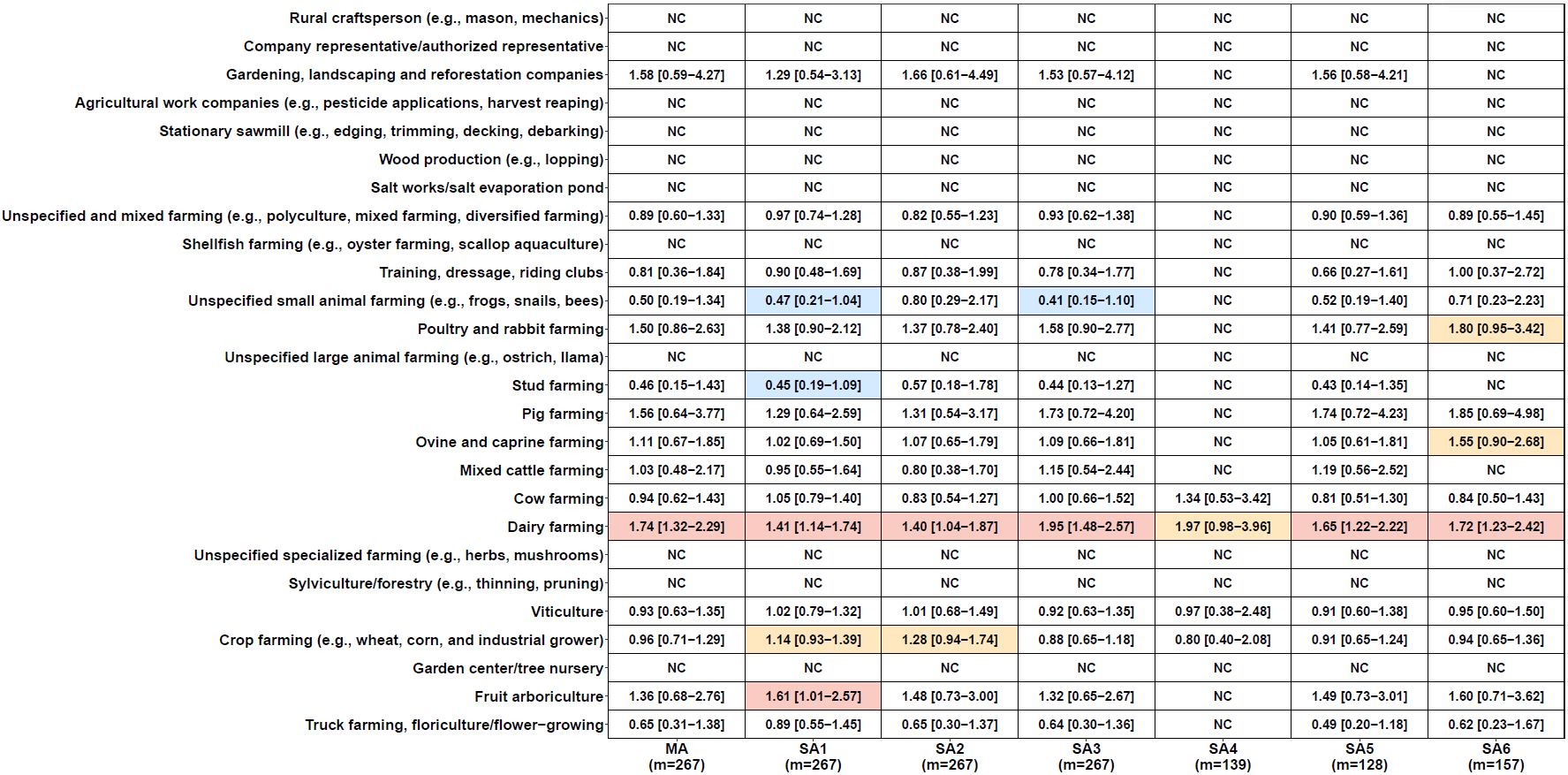


# **Figure S6**: Farming activities and risk of ulcerative colitis among female farm managers - comparison of the main analysis with sensitivity analyses

m: number of exposed cases, NC: not calculated (<3 exposed cases), SA: sensitivity analysis. The white cells represent instances where there is no difference in the risk of UC between FMs engaged in the specified activity and those who are not, while the red and green cells indicate an elevated and reduced risk of UC, respectively. Orange and blue cells refer to positive and negative trends, respectively. The main analysis was adjusted for age, first year of the farm’s establishment, farm location, number of pre-existing medical comorbidities, and the number of years performing the considered activity. SA1 was adjusted for age, first year of the farm’s establishment, farm location, pre-existing medical comorbidities (long-term illnesses), and the number of years performing the considered activity. SA2 was adjusted for age, first year of the farm’s establishment, farm location, and the number of years performing the considered activity. SA3 was adjusted for age, first year of the farm’s establishment, farm location, pre-existing mental health comorbidities (long-term illnesses for mental health issues such as depression and antidepressant drug reimbursement), and the number of years performing the considered activity. SA4 is an exposure-specific analysis for which the exposed group was restricted to all FMs who engaged in the specific activity for less than the median number of years of exposure of all exposed FMs (e.g., < 5 years). SA5 is an exposure-specific analysis for which the exposed group was restricted to all FMs who engaged in the specific activity for at least the median number of years of exposure of all exposed FMs (e.g., ≥ 5 years). Both SA4 and SA5 were adjusted for age, first year of the farm’s establishment, farm location, and number of pre-existing medical comorbidities. SA6 was restricted to cases identified from 2013-2016 and was adjusted for the same variables as the main analysis.


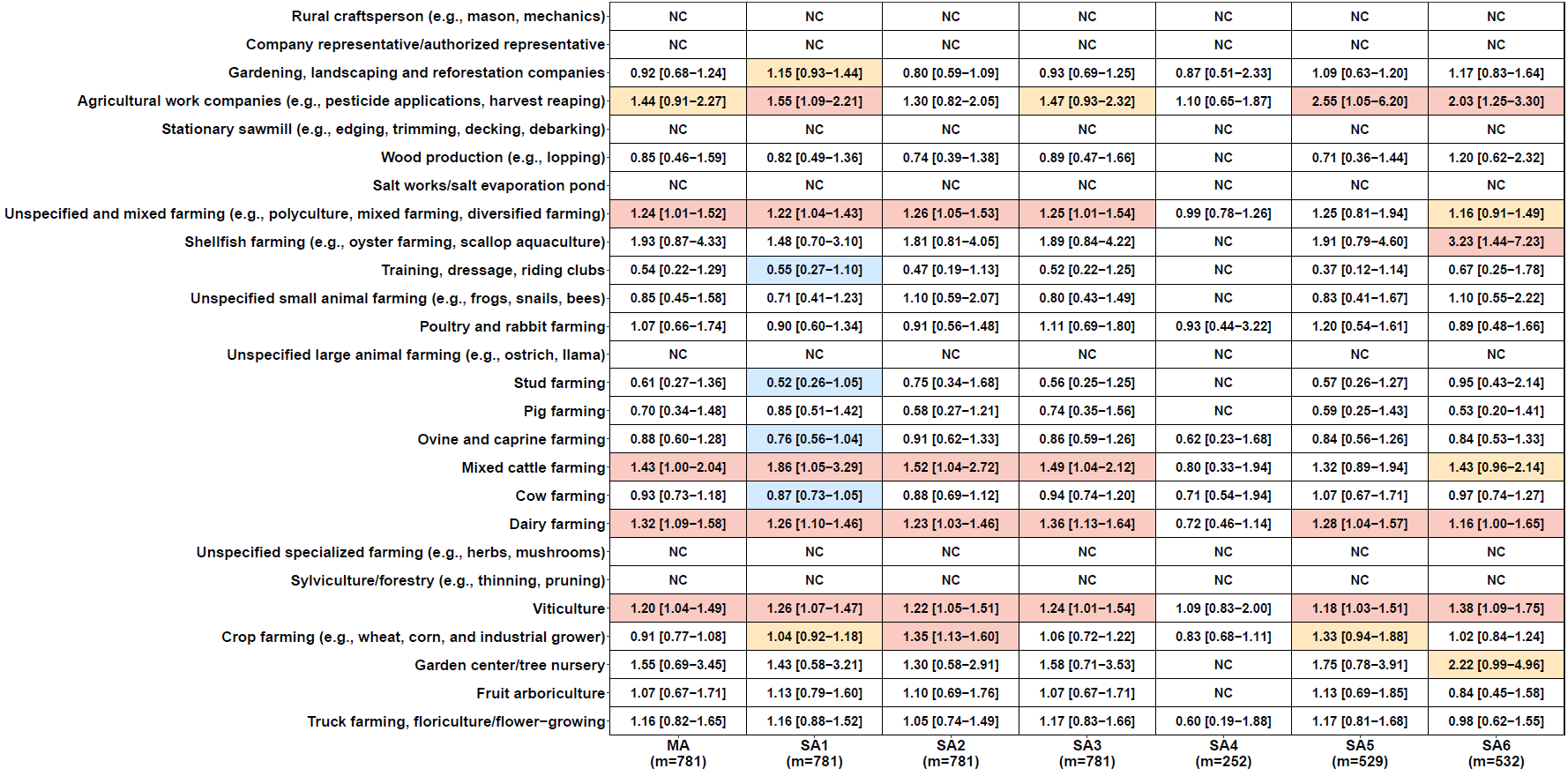


# **Figure S7**: Farming activities and risk of ulcerative colitis among male farm managers - comparison of the main analysis with sensitivity analyses

m: number of exposed cases, NC: not calculated (<3 exposed cases), SA: sensitivity analysis. The white cells represent instances where there is no difference in the risk of UC between FMs engaged in the specified activity and those who are not, while the red and green cells indicate an elevated and reduced risk of UC, respectively. Orange and blue cells refer to positive and negative trends, respectively. The main analysis was adjusted for age, first year of the farm’s establishment, farm location, number of pre-existing medical comorbidities, and the number of years performing the considered activity. SA1 was adjusted for age, first year of the farm’s establishment, farm location, pre-existing medical comorbidities (long-term illnesses), and the number of years performing the considered activity. SA2 was adjusted for age, first year of the farm’s establishment, farm location, and the number of years performing the considered activity. SA3 was adjusted for age, first year of the farm’s establishment, farm location, pre-existing mental health comorbidities (long-term illnesses for mental health issues such as depression and antidepressant drug reimbursement), and the number of years performing the considered activity. SA4 is an exposure-specific analysis for which the exposed group was restricted to all FMs who engaged in the specific activity for less than the median number of years of exposure of all exposed FMs (e.g., < 5 years). SA5 is an exposure-specific analysis for which the exposed group was restricted to all FMs who engaged in the specific activity for at least the median number of years of exposure of all exposed FMs (e.g., ≥ 5 years). Both SA4 and SA5 were adjusted for age, first year of the farm’s establishment, farm location, and number of pre-existing medical comorbidities. SA6 was restricted to cases identified from 2013-2016 and was adjusted for the same variables as the main analysis.


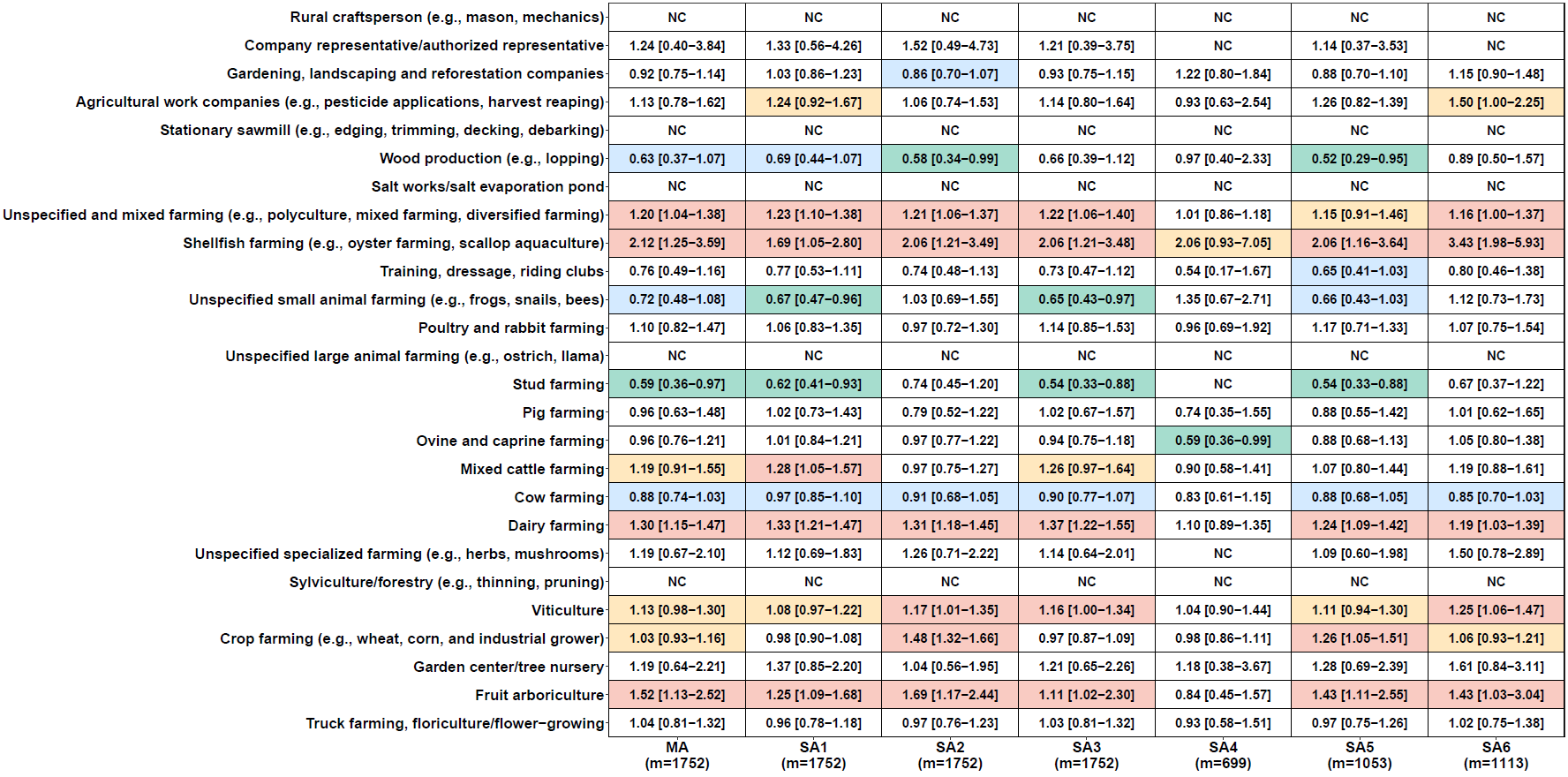


# **Figure S8**: Farming activities and risk of inflammatory bowel disease among all farm managers - comparison of the main analysis with sensitivity

m: number of exposed cases, NC: not calculated (<3 exposed cases), SA: sensitivity analysis. The white cells represent instances where there is no difference in the risk of IBD between FMs engaged in the specified activity and those who are not, while the red and green cells indicate an elevated and reduced risk of IBD, respectively. Orange and blue cells refer to positive and negative trends, respectively. The main analysis was adjusted for sex, age, first year of the farm’s establishment, farm location, number of pre-existing medical comorbidities, and the number of years performing the considered activity. SA1 was adjusted for sex, age, first year of the farm’s establishment, farm location, pre-existing medical comorbidities (long-term illnesses), and the number of years performing the considered activity. SA2 was adjusted for sex, age, first year of the farm’s establishment, farm location, and the number of years performing the considered activity. SA3 was adjusted for sex, age, first year of the farm’s establishment, farm location, pre-existing mental health comorbidities (long-term illnesses for mental health issues such as depression and antidepressant drug reimbursement), and the number of years performing the considered activity. SA4 is an exposure-specific analysis for which the exposed group was restricted to all FMs who engaged in the specific activity for less than the median number of years of exposure of all exposed FMs (e.g., < 5 years). SA5 is an exposure-specific analysis for which the exposed group was restricted to all FMs who engaged in the specific activity for at least the median number of years of exposure of all exposed FMs (e.g., ≥ 5 years). Both SA4 and SA5 were adjusted for sex, age, first year of the farm’s establishment, farm location, and number of pre-existing medical comorbidities. SA6 was restricted to cases identified from 2013-2016 and was adjusted for the same variables as the main analysis.


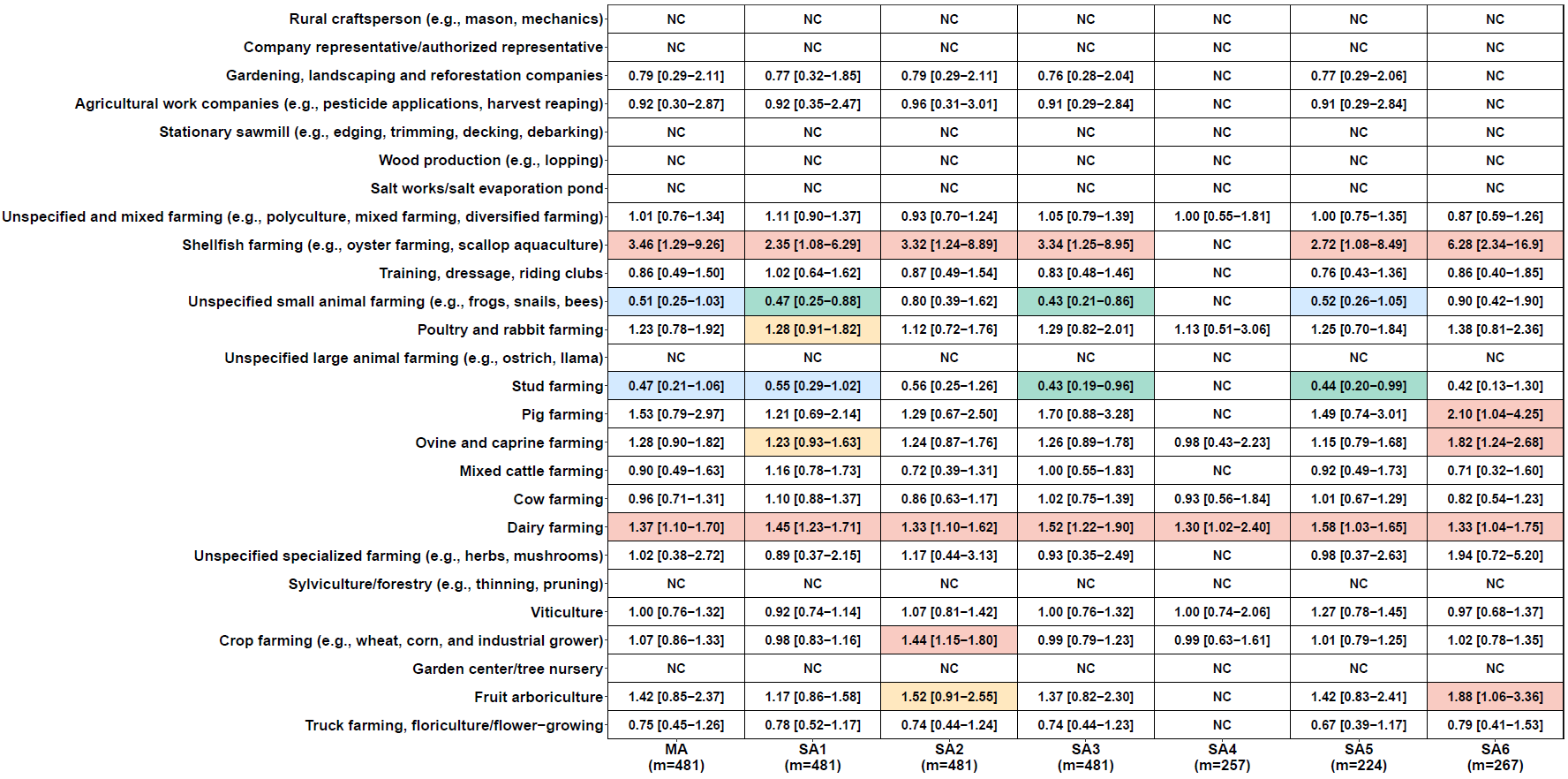


# **Figure S9**: Farming activities and risk of inflammatory bowel disease among female farm managers - comparison of the main analysis with sensitivity analyses

m: number of exposed cases, NC: not calculated (<3 exposed cases), SA: sensitivity analysis. The white cells represent instances where there is no difference in the risk of IBD between FMs engaged in the specified activity and those who are not, while the red and green cells indicate an elevated and reduced risk of IBD, respectively. Orange and blue cells refer to positive and negative trends, respectively. The main analysis was adjusted for age, first year of the farm’s establishment, farm location, number of pre-existing medical comorbidities, and the number of years performing the considered activity. SA1 was adjusted for age, first year of the farm’s establishment, farm location, pre-existing medical comorbidities (long-term illnesses), and the number of years performing the considered activity. SA2 was adjusted for age, first year of the farm’s establishment, farm location, and the number of years performing the considered activity. SA3 was adjusted for age, first year of the farm’s establishment, farm location, pre-existing mental health comorbidities (long-term illnesses for mental health issues such as depression and antidepressant drug reimbursement), and the number of years performing the considered activity. SA4 is an exposure-specific analysis for which the exposed group was restricted to all FMs who engaged in the specific activity for less than the median number of years of exposure of all exposed FMs (e.g., < 5 years). SA5 is an exposure-specific analysis for which the exposed group was restricted to all FMs who engaged in the specific activity for at least the median number of years of exposure of all exposed FMs (e.g., ≥ 5 years). Both SA4 and SA5 were adjusted for age, first year of the farm’s establishment, farm location, and number of pre-existing medical comorbidities. SA6 was restricted to cases identified from 2013-2016 and was adjusted for the same variables as the main analysis.


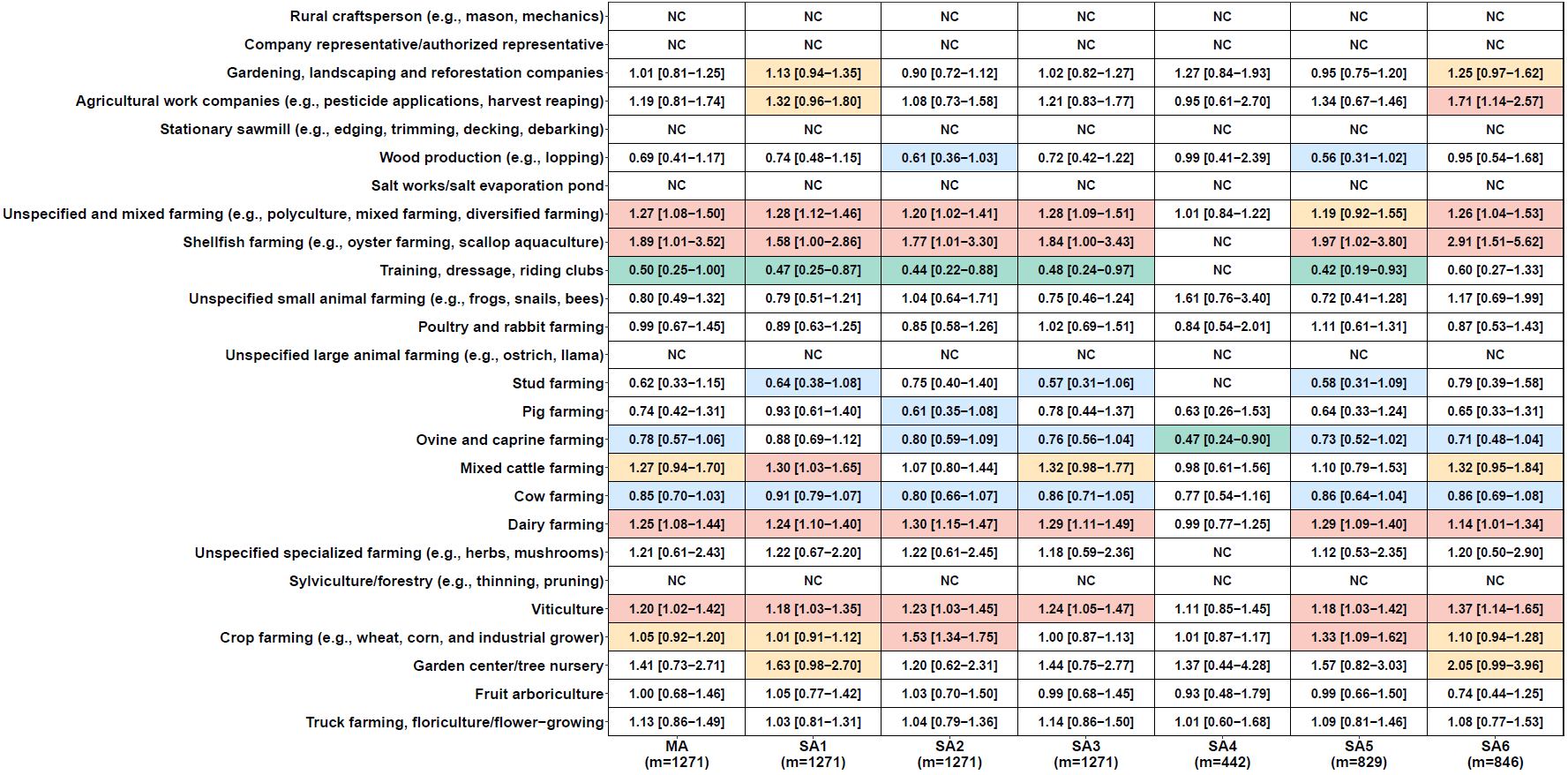


# **Figure S10**: Farming activities and risk of inflammatory bowel disease among male farm managers - comparison of the main analysis with sensitivity analyses

m: number of exposed cases, NC: not calculated (<3 exposed cases), SA: sensitivity analysis. The white cells represent instances where there is no difference in the risk of IBD between FMs engaged in the specified activity and those who are not, while the red and green cells indicate an elevated and reduced risk of IBD, respectively. Orange and blue cells refer to positive and negative trends, respectively. The main analysis was adjusted for age, first year of the farm’s establishment, farm location, number of pre-existing medical comorbidities, and the number of years performing the considered activity. SA1 was adjusted for age, first year of the farm’s establishment, farm location, pre-existing medical comorbidities (long-term illnesses), and the number of years performing the considered activity. SA2 was adjusted for age, first year of the farm’s establishment, farm location, and the number of years performing the considered activity. SA3 was adjusted for age, first year of the farm’s establishment, farm location, pre-existing mental health comorbidities (long-term illnesses for mental health issues such as depression and antidepressant drug reimbursement), and the number of years performing the considered activity. SA4 is an exposure-specific analysis for which the exposed group was restricted to all FMs who engaged in the specific activity for less than the median number of years of exposure of all exposed FMs (e.g., < 5 years). SA5 is an exposure-specific analysis for which the exposed group was restricted to all FMs who engaged in the specific activity for at least the median number of years of exposure of all exposed FMs (e.g., ≥ 5 years). Both SA4 and SA5 were adjusted for age, first year of the farm’s establishment, farm location, and number of pre-existing medical comorbidities. SA6 was restricted to cases identified from 2013-2016 and was adjusted for the same variables as the main analysis.


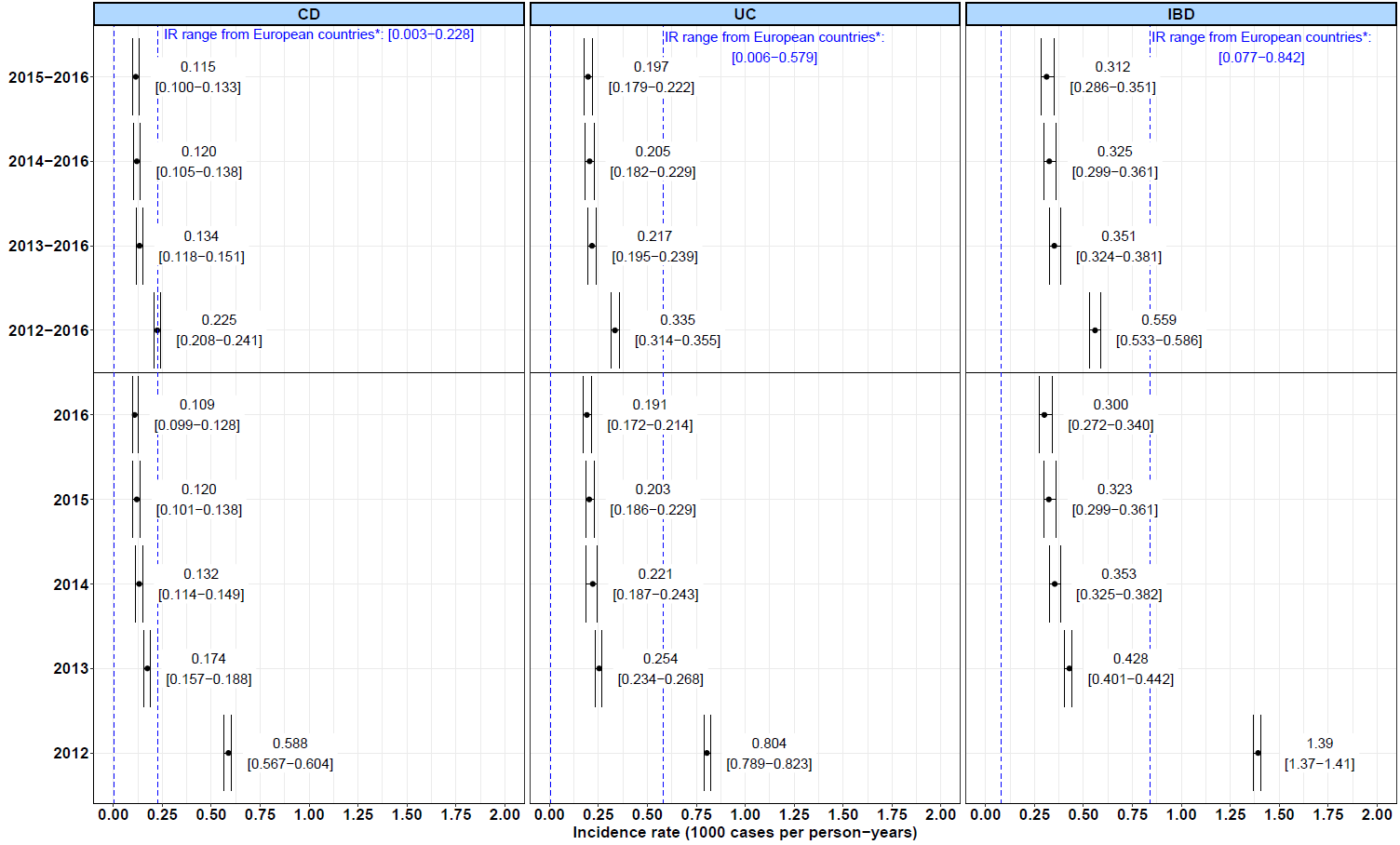


# **Figure S11**: Incidence rate per year and time period for each IBD type

CD: Crohn’s disease, IBD: inflammatory bowel disease, IR: incidence rate, UC: ulcerative colitis.

*The range of incidence rates from European countries was based on the following references:

1. Ananthakrishnan AN. Epidemiology and risk factors for IBD. *Nat Rev Gastroenterol Hepatol* 2015;12(4):205–217.

2. Boks M, Lilja M, Widerström M, et al. Increased incidence of late-onset inflammatory bowel disease and microscopic colitis after a Cryptosporidium hominis outbreak. *Scand J Gastroenterol* 2022;57(12):1443-1449.

3. Gower-Rousseau C, Leroyer A, Génin M, et al. Épidémiologie descriptive et évolution dans le temps et l'espace de l'incidence des maladies inflammatoires chroniques intestinales dans le nord-ouest de la France (1988-2014). *Bull Epidémiol Hebd* 2019;13:228–236. [in French]

4. Kaplan GG, Windsor JW. The four epidemiological stages in the global evolution of inflammatory bowel disease. *Nat Rev Gastroenterol Hepatol* 2021;18(1):56-66.

5. Kirchgesner J, Lemaitre M, Rudnichi A, et al. Therapeutic management of inflammatory bowel disease in real-life practice in the current era of anti-TNF agents: analysis of the French administrative health databases 2009-2014. *Aliment Pharmacol Ther* 2017;45(1):37–49.

6. Mak WY, Zhao M, Ng SC, et al. The epidemiology of inflammatory bowel disease: East meets west. *J Gastroenterol Hepatol* 2020;35(3):380–389.

7. Ng SC, Shi HY, Hamidi N, et al. Worldwide incidence and prevalence of inflammatory bowel disease in the 21st century: a systematic review of population-based studies. *Lancet* 2017;390(10114):2769-2778.

8. Ye Y, Pang Z, Chen W, Ju S, Zhou C. The epidemiology and risk factors of inflammatory bowel disease. *Int J Clin Exp Med* 2015;8(12):22529-22542.
